# A multi-label learning model for predicting drug-induced pathology in multi-organ based on toxicogenomics data

**DOI:** 10.1371/journal.pcbi.1010402

**Published:** 2022-09-07

**Authors:** Ran Su, Haitang Yang, Leyi Wei, Siqi Chen, Quan Zou

**Affiliations:** 1 School of Computer Science and Technology, College of Intelligence and Computing, Tianjin University, Tianjin, China; 2 School of Software, Shandong University, Jinan, Shandong, China; 3 Yangtze Delta Region Institute (Quzhou), University of Electronic Science and Technology of China, Quzhou, Zhejiang, China; University at Buffalo - The State University of New York, UNITED STATES

## Abstract

Drug-induced toxicity damages the health and is one of the key factors causing drug withdrawal from the market. It is of great significance to identify drug-induced target-organ toxicity, especially the detailed pathological findings, which are crucial for toxicity assessment, in the early stage of drug development process. A large variety of studies have devoted to identify drug toxicity. However, most of them are limited to single organ or only binary toxicity. Here we proposed a novel multi-label learning model named Att-RethinkNet, for predicting drug-induced pathological findings targeted on liver and kidney based on toxicogenomics data. The Att-RethinkNet is equipped with a memory structure and can effectively use the label association information. Besides, attention mechanism is embedded to focus on the important features and obtain better feature presentation. Our Att-RethinkNet is applicable in multiple organs and takes account the compound type, dose, and administration time, so it is more comprehensive and generalized. And more importantly, it predicts multiple pathological findings at the same time, instead of predicting each pathology separately as the previous model did. To demonstrate the effectiveness of the proposed model, we compared the proposed method with a series of state-of-the-arts methods. Our model shows competitive performance and can predict potential hepatotoxicity and nephrotoxicity in a more accurate and reliable way. The implementation of the proposed method is available at https://github.com/RanSuLab/Drug-Toxicity-Prediction-MultiLabel.

## Author summary

Drug-induced toxicity damages the health and is one of the key factors causing drug withdrawal from the market. Hence, to fully assess drug-induced toxicity, it is important to predict the detailed pathological findings, which are also crucial for toxicity mechanism understanding. However, most of the existing toxicity studies only predict binary toxicity (the toxicity or non-toxicity) or only predict the toxicity targeting single organ. The pathological findings of multiple organs are not well explored. Here we show, through the proposed Att-RethinkNet, it is possible to predict drug-induced pathological findings on both liver and kidney. Our results suggest that the Att-RethinkNet predicts potential hepatotoxicity and nephrotoxicity in a more accurate and reliable way, and it is applicable in multiple organs and takes account the compound type, dose, and administration time, so it is more comprehensive and generalized than the existing methods. The accurate prediction of pathological findings on multiple organs may benefit drug development.

This is a *PLOS Computational Biology* Methods paper.

## Introduction

Drug development consists of activities involving in bringing a new drug from laboratory to market. It is typically divided into four distinct and essential phases: drug discovery, preclinical research, clinical research, and approval and marketing, which is relatively expensive and time-consuming, and is filled with risk and uncertainty. Through systematic review of statistics concerning the cost of drug development, researchers find that companies spend 10 to 15 years and millions of dollars in obtaining a new drug into the market [1, 2]. However, failure rate of new drug candidates is still considerably high for many reasons, and drug-induced toxicity, including adverse reactions and toxic effects, is a common reason for drug withdrawal or discontinuation [3]. Drug-induced toxicity, which is assessed by the pathological findings with respect to the phenotypic end point, refers to the negative effects of medications, that is, dysfunctions and tissue lesions caused by the interaction of various chemical substances, which may cause adverse health issues. Since kidney and liver are filters for various regions of the body, they are the primary targets of toxins [4] and reports have shown that a great deal of drug failure is due to the hepatotoxicity and nephrotoxicity [5]. Thus, it is necessary to identify drug-induced hepatotoxicity/nephrotoxicity, especially the pathological findings caused by hepatotoxicity/nephrotoxicity in the early stage of drug development and eliminate toxic compounds as soon as possible so that the success rate of drug candidate trials can be greatly improved.

In the past, it is common to predict drug-induced toxicity through wet-lab experiments. Although such type of experiment is irreplaceable, it requires specially designed room, safety equipment, professional researchers, etc., which is a costly and inconvenient procedure. Therefore, a growing number of researchers are interested in in-silico techniques because computational approaches are usually cost-effective, which provides guidance for developing a new pharmaceutical drug and assists researchers assessing drug safety risks during drug development. In recent years, micro-array technology in toxicology, known as toxicogenomics, is becoming a broadly used method for determination of potential toxicity of a new chemical entity [6–8]. Toxicogenomics data plays an important role in understanding and predicting drug-induced toxicity, and the application of gene expression data prompts researchers to solve biological problems through data analysis methods. The analysis of gene expression profiles in target organs after drug treatment can be used to assist in detecting potential toxicity before the appearance of a toxic phenotype [9–13]. Presently, some databases such as Open TG-GATEs (Toxicogenomics Project-Genomics Assisted Toxicity Evaluation System), which is one of the largest public toxicogenomics databases have been built for toxicity research [14]. And an increasing number of studies have focused on using the gene expression profiles and toxicity information for the identification of the potential negative effects of drugs.

Many researchers have carried out a series of toxicity exploration on target organs. Zhu et al. constructed random forest models based on three types of descriptors to predict drug-induced liver injury (DILI), the models based on under-sampling and over-sampling to deal with unbalanced data sets both achieved promising performance [15]. Minowa et al. proposed a prediction model based on gene expression profiles for predicting drug-induced proximal tubular injury in rats, and found that there were differentially expressed in a number of genes at 24h after a single dose administration, which improved the predictive powder of the model to a certain extent [16]. On this basis, An et al. tried to consider the toxicity of two organs at the same time, and developed four computational models to classify whether a drug is liver toxic or liver-kidney toxic. The models used artificial neural network (ANN), k-nearest neighbor (kNN), linear discriminant analysis (LDA), and support vector machine (SVM) respectively, and all prediction accuracy of them were more than 90% [17]. Zhang et al. mapped thousands of drug side effects to multiple labels, integrated the base predictors according to the weighted scoring ensemble strategy, and finally obtained a high-precision ensemble model [18]. Raies et al. used binary relevance and classifier chains methods to predict multiple toxicity endpoints for the same compound, and the comparative calculation results showed that the classifier chain algorithm achieved better performance [19]. Su et al. developed a series of models for hepatotoxicity prediction, where dose information and biological context were sufficiently explored, and provided a fitting method of dose-response curve [20, 21]. Jinwoo et al. employed gene-expression data, explored co-occurrences of pathologies, and proposed an integrative model to predict multiple organ pathologies, which is an advanced method to predict multiple pathology to the best of our knowledge [22]. The integrative model built a KNN classifier for each pathology and extracted the pathology associations to calculate the final scores. The accuracy of the prediction model ranges from 80% to 97% in both liver and kidney.

After review of recent research, we conclude that despite high accuracy performances achieved by several studies, existing works still have limitations. Firstly, most of prior studies focused on the prediction of toxicity (toxic or non-toxic) in a certain organ. Pathological finding prediction has not been explored much considering its importance for toxicity assessment. The handful existing pathology predictive models developed individual model for each label [19], which neglected the fact that compounds might cause several toxic effects simultaneously. Secondly, the pathological finding prediction is a multi-label classification task. In recent years, many multi-label classification methods have been proposed in the field of biological information [23, 24]. Nevertheless, most of the existing multi-label classification models in toxicogenomics area still used traditional machine learning models such as binary relevancy (BR) or classier chain (CC). The advanced deep learning technology has not been tested and employed. Directly applying existing deep learning network often obtains unsatisfactory results due to different characteristics of the toxicogenomics data, so it is required to build proper deep architecture with careful design. Lastly, some studies show very limited applicability for toxicity identification due to the adoption of small-scale, single-dose and single-time point data, thus data considering various factors should be fully adopted.

In this paper, we proposed a novel multi-label learning model, named Att-RethinkNet, for predicting drug-induced toxicity in multi-organ based on toxicogenomics data. Instead of handling the binary classification problem that differentiating whether a compound is toxic or non-toxic, we identified the specific pathological findings of liver and kidney, which is a multi-label learning task. To overcome the shortcomings such as ignoring label correlation in the traditional multi-label classification, inspired by Yang et al.’s work [25] which was evaluated on dozen multi-label data sets and justifies that RethinkNet obtains a better performance than state-of-the-art algorithms for multi-label classification tasks, we designed the deep framework Att-RethinkNet, which is equipped with a memory structure and can effectively utilize the label correlation information. Besides, attention mechanism is embedded to focus on the important features and obtain better feature presentation. The Att-RethinkNet, which is applicable in multiple organs and takes account the compound type, dose, and administration time, is considerably more comprehensive than existing models. And more importantly, it predicts multiple pathological findings simultaneously, instead of predicting each pathology separately as the previous model did. To the best of our knowledge, our Att-RethinkNet is the first to explore the multiple pathological findings based on deep architecture. Experiment results on Open TG-GATES show the efficacy and efficiency of the proposed method.

**Fig 1:**
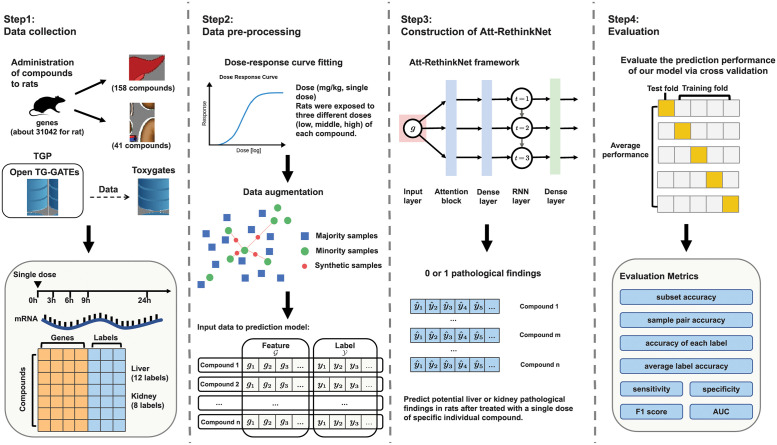
The work flow of the proposed method. We have four steps for the proposed method, data collection, data pre-processing, construction of the proposed Att-RethinkNet model and evaluation of the Att-RethinkNet.

Fig 1 shows an overview of predicting pathological findings in this study. There are mainly four steps, including data collection, data pre-processing, construction of the proposed Att-RethinkNet model and evaluation of the Att-RethinkNet. The details of each step will be introduced in the following materials and methods section. The implementation of the Att-RethinkNet can be found at https://github.com/RanSuLab/Drug-Toxicity-Prediction-MultiLabel.

## Materials and methods

### Step 1 & Step 2: Data collection and pre-processing

We used the Open TG-GATEs to train and validate our model. TG-GATEs is a large-scale toxicogenomics database developed by the Japanese Toxicogenomics Project (TGP) [14]. The database includes gene expression profiles and toxicological data of 170 compounds, derived from *in vitro* experiments using human primary hepatocytes and rat primary hepatocytes and *in vivo* experiments in rat at different dosages and time points [26, 27]. We also used data extracted from Toxygates which was released as an integrated, easily accessible and user-friendly platform for the Open TG-GATEs toxicogenomics data analysis [28]. Toxygates uses the Bioconductor *affy* package in R to carry out data normalization of each sample. Toxygates enables users to directly extract the correlation between gene expression and variables (such as dose level and exposure time) from the original microarray data of Open TG-GATEs, displays the gene expression data in human readable form, and convert the binary file in CEL format into CSV files.

In our studies, we used *in vivo* gene expression profiling of liver and kidney from rats at 24h of all three dose levels (low, middle, and high). For the liver data, rats were exposed to 158 compounds and expression levels of 31,042 mRNAs were collected. For the kidney data, rats were exposed to 41 compounds and also expression levels of 31,042 mRNAs were collected. The 41 compounds of kidney data were all included in the liver data so they were tested on both organs. However, for other compounds tested for liver, the potential pathological risk to kidney is unknown. The drugs or chemical compounds involved in the experimental data are shown in S1 Table. We next examined the *in vivo* hepatic and renal pathology taking place at four time points (3h, 6h, 9h, 24h) from TG-GATEs and focused only on the pathological findings that can be induced by larger than or equal to 5 compounds.

According to TG-GATEs, pathologists described drug-induced pathological symptoms obtained from *in vivo* tests using a controlled vocabulary. In our experiments, we targeted 20 pathological findings that comprise 12 liver pathological findings, including Cellular infiltration (CI), Eosinophilic change (EC), Hypertrophy (HY), Increased mitosis (IM), NOS lesion (NL), Microgranuloma (MI), Necrosis (NE), Hepatodiaphragmatic nodule (HN), Kupffer cell proliferation (KCP), Single cell necrosis (SCN), Swelling (SW), and Cytoplasmic vacuolization (CV) and 8 kidney pathological findings, including Hyaline cast (HC), Lymphocyte cellular infiltration (LCI), Basophilic change (BC), Cyst (CY), Dilatation (DI), Cystic dilatation (CD), Necrosis (NE), and Regeneration (RE). For multi-label problems, The label vector consists of 20 “1” or “0”, where the “1” represents a pathological finding exists, and “0” shows that the pathological findings does not exist.

We fitted a smooth sigmoid dose-response curve and extracted the maximum response (R_max_) from the curve, which contained comprehensive biological information and was proved a proper presentation of the curve in our previous study [20]. We removed the genes if the expression values at three doses could not form the dose-response curve and finally 6009 and 8485 genes were picked for liver and kidney. Then, according to the characteristics of the data we collected, we improved the MLSMOTE (Multilabel Synthetic Minority Over-sampling Technique) algorithm [29] to effectively handle imbalanced data set for multi label classification, which can overcome the issue of information loss in majority class samples, and avoid over-fitting caused by replication of the minority class samples. We added a judgment in the original method to avoid the rare case of generating samples with all labels being 0, which can enrich the information of samples to a greater extent. The steps, calculation formulas and advantages of improved MLSMOTE algorithm was summarized in S1 Text. We also presented the results of metrics that measure the imbalance ratio of data set before and after the MLSMOTE in S1 Text, and indicated the number of samples liver or kidney had in the majority and minority classes of original data. After data augmentation, we obtained 16,460 samples and 16,268 samples for liver and kidney respectively.

### Step 3: Construction of Att-RethinkNet

#### Review of traditional multi-label learning algorithms

Multi-label learning aims at training models to tackle problems where each sample is associated with multiple labels simultaneously. We here reviewed two commonly used multi-label classification methods, binary relevance (BR) and classifier chains (CC) in this section. Both BR and CC decompose the multi-label classification task into multiple binary classification problems. BR treats the prediction of each label as an independent binary classification problem, where each classifier is trained by all the features and only a single label needs to be predicted. Since each label is treated individually, this algorithm ignores possible correlations among the labels of the training data. CC is an extension of BR. In the CC approach, a series of binary classifiers are constructed according to label order and the binary assignments of preceding class labels are treated as the additional features [30]. CC adds labels into feature space, so the relationship among classified labels can be considered in the rest classifiers, which overcomes the weakness of BR and usually reports a better performance.

We compared the proposed method with BR and CC algorithms in our studies. For every binary classification task, we choose logistic regression (LR), random forest (RF), linear support vector machines (SVM) as base classifiers, and optimized the parameter *C* of LR, two hyper parameters *number of trees* and *maximum depth of trees* of RF, the parameter *C* of SVM using grid searching strategy. Finally, we evaluated the model performance via five-fold cross-validation.

#### RethinkNet

RethinkNet, a deep learning architecture for multi-label classification, is designed to mimic the “rethinking” process that human beings attempt to explore correlation between labels and solve multi-label problems more effectively through thinking the same issue over and over again until it is digestible. This process can be taken as a sequence prediction problem. The structure of RethinkNet is intuitive and understandable. It consists of two layers: recurrent neural network (RNN) layer and dense (fully connected) layer.

The RNN layer is used for a specific purpose: rethinking, an action that polishes the prediction result iteratively. RethinkNet adopts RNN to model the “rethinking” process and fully utilizes the RNN memory structure which stores temporary predictions on the labels from all classifiers. All classifiers receive the same information avoiding influence of the label order. Different from the CC which forms a chain of binary classifiers, a chain of multi-label classifiers as a sequence of rethinking is established [31]. On the dense layer, each neuron in the layer receives input from all the neurons present in its previous layer (the RNN layer) and transforms the output of previous RNN layer into the desired label vector, which generates the final prediction results. RethinkNet can well consider label correlation before the final prediction. Besides, the framework leverages cost-sensitive re-weighted loss function during learning phase and weights each label in the loss function according to the importance of the label.

#### The proposed method: Att-RethinkNet

Our proposed Att-RethinkNet, a novel deep learning architecture for multi-label classification, was designed based on the RethinkNet. To emphasize the more important genes, we embedded the attention mechanism in the model. The core idea of attention mechanism is to learn a weight distribution from existing data and then focus on the more important features, which enables the network to obtain better feature representation.

**Fig 2:**
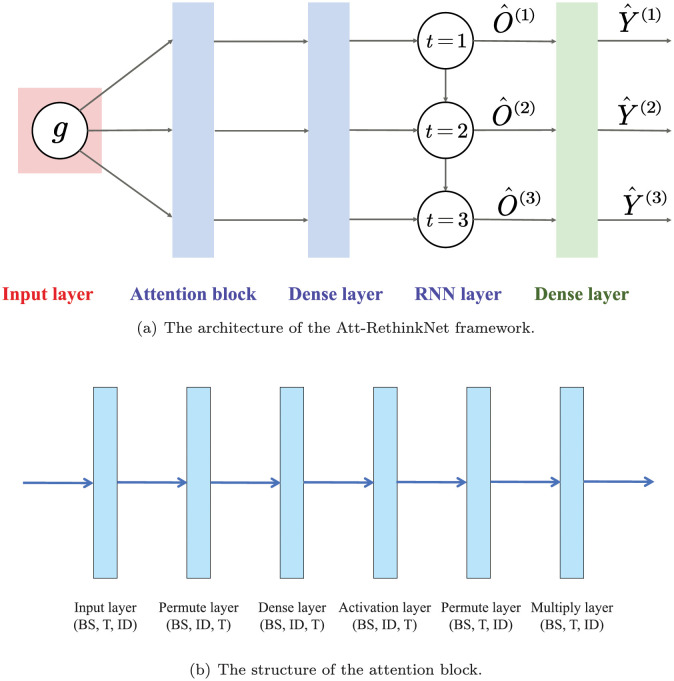
(a) is the architecture of the Att-RethinkNet framework, which is the specific description of our proposed deep neural network structure of Step3 in Fig 1. (b) is the structure of the attention block in Att-RethinkNet framework. BS means batch size. T means time step, depicting the number of iterations of each LSTM unit, without affecting the number of parameters. ID means input dimension. The first permute layer re-organizes the input layer, which permutes the T and ID dimensions of the input. The dense layer and the activation layer compute attention probabilities (the weight) for the input, that is, calculate the weight corresponding to each gene feature. The activation layer applies softmax function to the activated neurons. The second permute layer also re-organize the dimensions of the data whose weights have been calculated so that the multiplication can be operated in the multiply layer. The multiply layer is the last layer in attention block. In this layer, input of attention block times the probability vector of attention, achieving the weight allocation of feature vector. The attention mechanism assigns different importance to features which improves the result of classification greatly.

In our experiment, we implemented the attention mechanism between input layer and the RNN layer. To improve the performance, we used a modified version of RNN, Long Short-Term Memory (LSTM) networks, in the proposed Att-RethinkNet framework. The architecture of our proposed Att-RethinkNet includes the input layer, attention block, RNN layer, and dense layer as shown in Fig 2(a). The goal is to learn a function f:X→Y from a given training data set and realize the mapping from the feature vector $x\in X\subseteq R^{dim}$ to corresponding pathological findings label $Y\in Y\subseteq {\left\{0,1\right\}}^{L}$.

The input layer contains the gene feature. The RNN layer learns *T* iterations, and each iteration represents a thinking process. The output of RNN layer at t-th iteration is abbreviated as ${\hat{O}}^{\left(t\right)}$, which stands for the embedding of t-th prediction label vector ${\hat{Y}}^{\left(t\right)}$. By the same token, the information of ${\hat{O}}^{\left(t\right)}$ will be passed to (*t* + 1)-th iteration in the RNN layer, that is, Att-RethinkNet will use the temporary prediction results of the previous iteration to obtain better label predictions ${\hat{Y}}^{\left(t+1\right)}$. When *T* iterations are executed, ${\hat{Y}}^{\left(T\right)}$ is the final prediction. ${\hat{Y}}^{\left(T\right)}$ is an accurate set of labels that has been iteratively revised, which means labels that are difficult to predict will also have a greater probability of being classified into the correct category. In our experiments, we set *T* = 5 for liver data and *T* = 3 for kidney data, for the reason that the performance of our proposed model generally converges at the fixed *T*th iteration of rethinking. With the increase of *T*, the prediction accuracy of pathological findings basically did not change. We also show the structure of the attention block in Fig 2(b).

The pseudo-code of the proposed method to predict drug-induced pathological findings in multi-organ samples is shown in Algorithm 1.

**Algorithm 1** Drug-induced pathological finding prediction using K-fold cross validation.

**Input:**

1: Input: Gene expression data involving all compounds and all genes.

**Output:**

2: Output: Model to predict drug-induced pathological findings based on gene expression data.

3: Fit the dose-response curve based on three dose levels (low, middle and high), and select a proper measure to represent the full biological information of the curve.

4: Augment and balance the data.

5: Data normalization.

6: **for** *i* = 1; *i* < *K*; *i*++ **do**

7:  Divide the augmented data set *D* into test set *D*_*test*_ and training set *D*_*train*_.

8:  Feed *D*_*train*_ into Att-RethinkNet framework.

9:  Calculate attention probabilities and weight all genes

10:  Predict the potential pathological findings in multiple organs, and modify temporary prediction results iteratively.

11:  Test on *D*_*test*_ and record the results.

12: **end for**

13: Calculate the average of the *K* results, obtain the final evaluation results, and analyze the classification effect of our proposed model.

### Step 4: Evaluation

In this paper, all experiments were evaluated by five-fold cross-validation. In single-label classification, the traditional evaluation metrics can be used. In multi-label classification, a sample may have part of labels classified correctly, so evaluation measures are required to have an objective view of the performance of multi-label classifiers [32–34]. Therefore, we used two groups of evaluation metrics, one is sample-based metrics that compute the performance of each sample separately and then average it over all samples and the other is label-based metrics that conduct the evaluation in terms of each label and then take the macro/micro average over all labels [35]. Sample-based metrics including subset accuracy (ACC), sample pair accuracy (ACC_pair_) and accuracy of each label (ACC_lab_) and label-based metrics including average label accuracy (ACC_avelab_), macro sensitivity (SEN), macro specificity (SPE) and macro F1 score (F1) were used to evaluate our model. Assuming *x* is a sample, *n* is the number of test sample, and *Y*_*i*_ and $\hat{Y}\left(x_{i}\right)$ represent the true and predicted label vector for the *i*th sample, respectively, these metrics are defined as follows:

$$\begin{array}{c} \text{ACC}=\frac{1}{n}\sum_{i=1}^{n}\Phi \left(Y_{i}=\hat{Y}\left(x_{i}\right)\right) \end{array}$$ (1)

Where

$$\begin{array}{c} \Phi \left(\cdot \right)=\{\begin{array}{cc} 1, & \cdot \;\text{is}\;\text{true}\;\text{(if}\;\text{and}\;\text{only}\;\text{if}\;\hat{Y}\;\text{exactly}\;\text{matches}\;\hat{Y}\left(x_{i}\right)),\\ 0, & \text{otherwise.} \end{array} \end{array}$$ (2)

$$\begin{array}{c} {\text{ACC}}_{\text{pair}}=\frac{1}{n}\overset{n}{\underset{i=1}{\sum }}\frac{\left|Y_{i}\cap \hat{Y}\left(x_{i}\right)\right|}{\left|Y_{i}\cup \hat{Y}\left(x_{i}\right)\right|} \end{array}$$ (3)

$$\begin{array}{c} {\text{ACC}}_{\text{lab}(l)}=\frac{1}{n}\sum_{i=1}^{n}\left[\left[{\hat{y}}_{l}^{j}=y_{l}^{i}\right]\right] \end{array}$$ (4)

Subset accuracy is the fraction of samples whose predicted label vector is the same as the true label vector. For a predicted label vector of a test set, the classification result is considered to be correct if and only if the prediction value is exactly equal to the true value of label set. ACC_pair_ reflects the degree of partial correctness, which is more lenient than subset accuracy. ACC_lab_ represents the accuracy of each label, by which we can find which pathological finding is easy to identify [33]. When calculating label-based metrics, the basic statistics true positive (TP), false positive (FP), true negative (TN), and false negative (FN) for label *l* is defined as follow:

$$\begin{array}{c} {\text{TP}}_{l}=|\{x_{i}|y_{l}\in Y_{i}\wedge y_{l}\in \hat{Y}\left(x_{i}\right),1\leq i\leq n,1\leq l\leq L\}| \end{array}$$ (5)

$$\begin{array}{c} {\text{FP}}_{l}=|\{x_{i}|y_{l}\notin Y_{i}\wedge y_{l}\in \hat{Y}\left(x_{i}\right),1\leq i\leq n,1\leq l\leq L\}| \end{array}$$ (6)

$$\begin{array}{c} {\text{TN}}_{l}=|\{x_{i}|y_{l}\notin Y_{i}\wedge y_{l}\notin \hat{Y}\left(x_{i}\right),1\leq i\leq n,1\leq l\leq L\}| \end{array}$$ (7)

$$\begin{array}{c} {\text{FN}}_{l}=|\{x_{i}|y_{l}\in Y_{i}\wedge y_{l}\notin \hat{Y}\left(x_{i}\right),1\leq i\leq n,1\leq l\leq L\}| \end{array}$$ (8)

$$\begin{array}{c} {\text{ACC}}_{\text{avelab}}=\frac{1}{L}\sum_{l=1}^{L}\frac{{\text{TP}}_{l}+{\text{TN}}_{l}}{{\text{TP}}_{l}+{\text{TN}}_{l}+{\text{FP}}_{l}+{\text{FN}}_{l}} \end{array}$$ (9)

$$\begin{array}{c} \text{SEN}=\frac{1}{L}\sum_{l=1}^{L}\frac{{\text{TP}}_{l}}{{\text{TP}}_{l}+{\text{FN}}_{l}} \end{array}$$ (10)

$$\begin{array}{c} \text{SPE}=\frac{1}{L}\sum_{l=1}^{L}\frac{{\text{TN}}_{l}}{{\text{TN}}_{l}+{\text{FP}}_{l}} \end{array}$$ (11)

$$\begin{array}{c} F1=\frac{1}{L}\sum_{l=1}^{L}\frac{{2\text{TP}}_{l}}{{2\text{TP}}_{l}+{\text{FP}}_{l}+{\text{FN}}_{l}} \end{array}$$ (12)

Here *L* and *y*_*l*_ denote the number of labels and the *l*th true label, respectively. Additionally, we also adopted the receiver operating characteristics curve (ROC) and area under the curves (AUC) to get a multiple perspective on evaluation and assessment.

## Results

Here we firstly look into the features produced by the Att-RethinkNet based on LSTM and the outcome confusion matrix. Then we discussed the prediction performance of LSTM and SRN algorithms of the RNN layer. We compared the proposed method with the original RethinkNet and the traditional BR and CC. Additionally, we compared with the integrative model proposed by Kim et al. [22], which is the state-of-the-art work for drug-induced pathological finding prediction. We conducted all the experiments on Open TG-GATES *in vivo* liver and kidney data. In addition, in order to further verify the generalization of the model, we used an unseen and independent test set on the *in vitro* data set of rats.

### Visualization and confusion matrix of the prediction results

**Fig 3:**
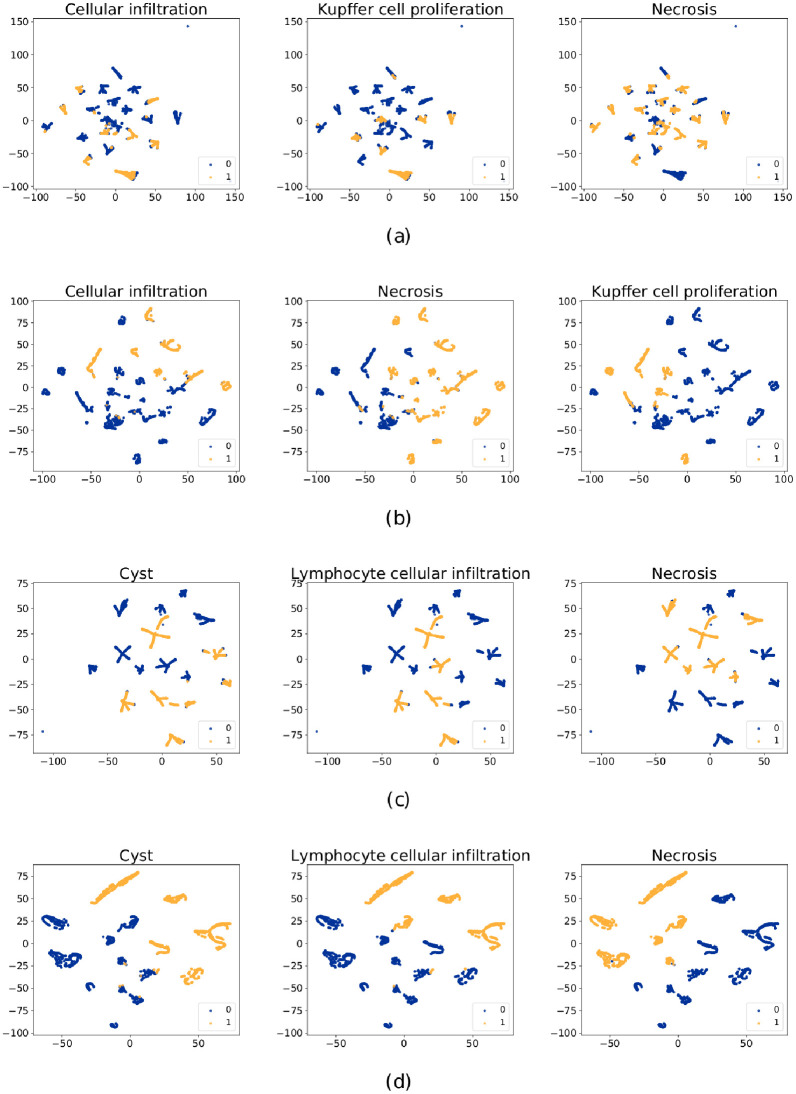
The t-SNE visualization of features for different pathological findings (from one fold). (a) and (b) shows the raw features and features generated after RNN layer, respectively. Three pathological findings cellular infiltration, necrosis and kupffer cell proliferation from liver data are involved. (c) and (d) shows the raw features and features generated after RNN layer, respectively and three pathological findings cyst, lymphocyte cellular infiltration and necrosis from kidney data are involved. The blue points represent 0 (no findings) and the yellow points represent 1 (with findings). The visualization of all targeted pathological findings using t-SNE can be found in S1 Fig for liver and S2 Fig for kidney.

Firstly, we performed t-distributed stochastic neighbor embedding (t-SNE) to visualize the data in a low dimension space. Raw features (genes) and features produced after RNN layer are shown in Fig 3. Here we show pathological findings cellular infiltration, necrosis and kupffer cell proliferation from liver data and cyst, lymphocyte cellular infiltration and necrosis from kidney data.

As can be seen from Fig 3, positive and negative samples with the raw features are mixed and have much overlapping. But after the Att-RethinkNet, the 0–1 classes can be better separated. This has indicated that the generated features are more distinctive and informative than the raw features.

**Fig 4:**
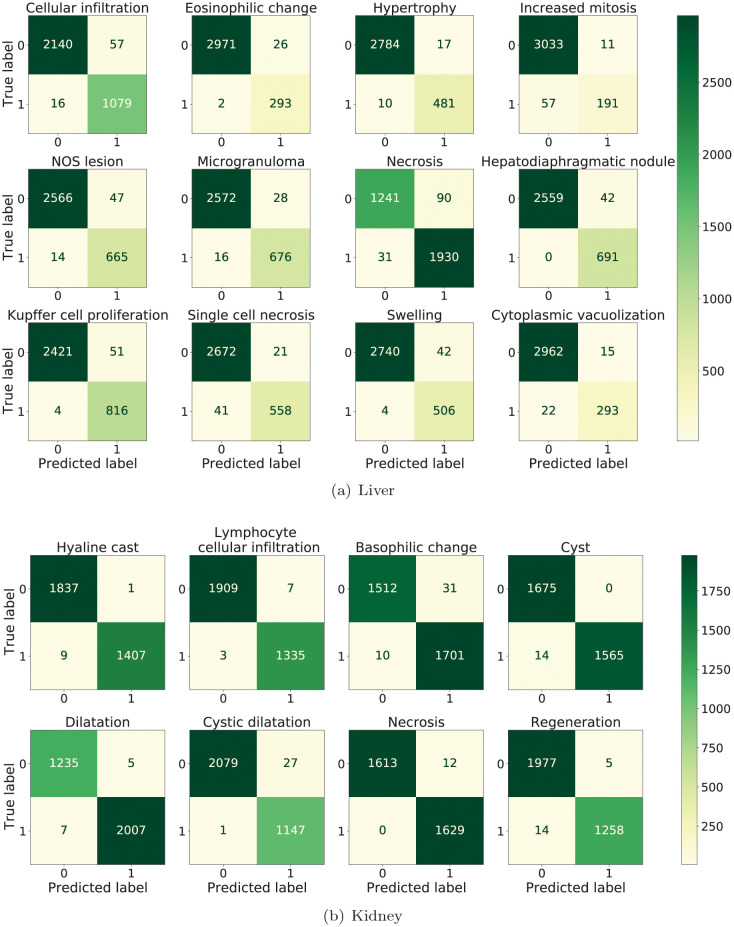
The confusion matrix of the pathology classification. The top-left represents the TN, the top-right represents FP, the bottom-left is FN and the bottom-right is TP. (a) shows the confusion matrix on liver data and (b) shows the confusion matrix on kidney data. The results were obtained from the first fold. The results of other folds are shown in S3 Fig.

To have a more granular understanding of the results of the proposed model, we show the confusion matrix of the pathology classification results in Fig 4. The values of the rows and columns represent the true and predicted labels on test data, respectively. From the confusion matrix, it is clear that the model has quite small values of FP and FN compared to TP and TN, and therefore low FP and FN rate. This has shown an impressive performance of the proposed model.

### Comparison between Att-RethinkNet with LSTM and SRN

In our experiment, the RNN layer of Att-RethinkNet adopts the LSTM network. Its advantage is that it not only attaches multiple relevant pathological findings to an input data and stores temporary predictions from earlier operations through memory mechanism, but also selectively forgets the prediction of previous labels through forget gate. In order to prove the effectiveness of applying LSTM algorithm in improving the classification effect, we compared and analyzed the methods of using LSTM and simple recurrent network (SRN) in RNN layer. Table 1 lists the classification results of the two algorithms on the liver data and kidney data. In the prediction of pathological findings of hepatotoxicity, the proposed Att-RethinkNet model implemented by LSTM algorithm obtained an ACC of 89.4%, which was 1.9% higher than that of the model using SRN, and obtained higher values in all evaluation metrics except SPE. The classification results of nephrotoxic pathological findings showed that the classification results of LSTM were also higher than SRN, and the improvement level of ACC exceeded 1.0%.

**Table 1:** Comparison between Att-RethinkNet based on LSTM and SRN algorithms.

ORG^1^	ALG^2^	ACC (%)	SEN (%)	SPE (%)	F1 (%)	AUC	ACC_pair_(%)	ACC_avelab_(%)
Liver	SRN	87.6	91.9	98.3	92.5	0.9921	90.2	97.4
	LSTM	89.4	94.2	98.2	93.8	0.9930	92.2	97.8
Kidney	SRN	96.4	98.7	99.4	98.9	0.9947	97.6	99.1
	LSTM	97.5	99.1	99.5	99.2	0.9949	98.1	99.3

^1^ ORG means the data set of target organ.

^2^ ALG represents the network structure actually adopted in RNN layer.

The reason for the promising classification accuracy is that the gate structure within LSTM and the internal complex training parameters improve the processing ability of the model for long sequence data and avoid the problem of vanishing gradients in RNN. More specifically, in the process of building Att-RethinkNet for predicting drug-induced pathological findings in multiple organ, LSTM algorithm provides a new improvement strategy for rethinking of RNN layer. When a group of pathological finding prediction labels are obtained through one iteration, one part is produced as the temporary result of the current iteration, and the other part of the information continues to be transmitted. And at the beginning of the next iteration, LSTM no longer directly uses the results of the previous iteration for better prediction, but determines the forgetting degree of the information through the forget gate. Finally, through selective *T* times iterative thinking, our Att-RethinkNet model based on LSTM can better analyze the implicit association between gene expression data and corresponding pathological findings, as well as the internal impact between different pathological findings, and then iteratively polish the multi-label prediction results, provide a more accurate label set and show more accurate classification results.

**Fig 5:**
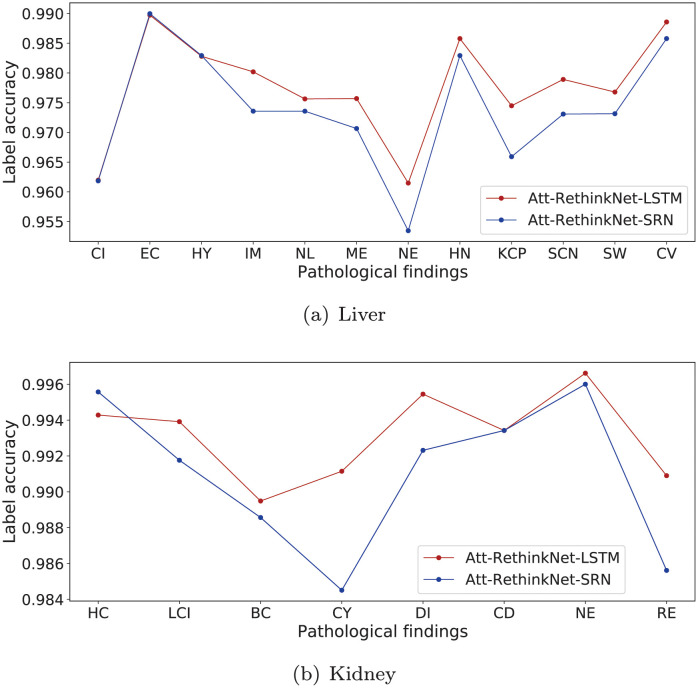
The ACC_lab_ of deep learning experiments in liver data (a) and kidney data (b).

Tables 2 and 3 show the ACC_lab_ values of the RNN layer of our proposed model in the liver and kidney data sets using two algorithms respectively. It can be seen that Att-RethinkNet based on LSTM has higher ACC_lab_ for the drug test set, and the prediction accuracy of 20 pathological labels is basically more than 97%, which shows that Att-RethinkNet based on LSTM can give reasonable prediction accuracy for each label. The more intuitive comparison of ACC_lab_ is shown in Fig 5. Although there is no significant difference in the prediction accuracy of each label between the proposed model implemented by LSTM and SRN, compared with the experiments based on SRN, we used LSTM algorithm as the core of classifier in RNN layer and still obtained a slightly higher accuracy in most tasks of drug pathological findings prediction.

**Table 2:** ACC_lab_ of all pathological findings for Att-RethinkNet based on LSTM and SRN for liver data set.

ALG/PF^1^	CI	EC	HY	IM	NL	MI	NE	HN	KCP	SCN	SW	CV
SRN	96.2	98.9	98.3	97.4	97.4	97.1	95.3	98.3	96.6	97.3	97.3	98.6
LSTM	96.2	99.0	98.3	98.0	97.6	97.6	96.1	98.6	97.4	97.9	97.7	98.9

^1^ ALG represents the network structure actually adopted in RNN layer. Values of columns 2 to 13 indicate the ACC_lab_ (%) of the corresponding pathological finding.

**Table 3:** ACC_lab_ of all pathological findings for Att-RethinkNet based on LSTM and SRN for kidney data set.

ALG/PF^1^	HC	LCI	BC	CY	DI	CD	NE	RE
SRN	99.6	99.2	98.8	98.5	99.2	99.3	99.6	98.6
LSTM	99.4	99.4	98.9	99.1	99.5	99.3	99.7	99.1

^1^ ALG represents the network structure actually adopted in RNN layer. Values of columns 2 to 9 indicate the ACC_lab_ (%) of the corresponding pathological finding.

**Fig 6:**
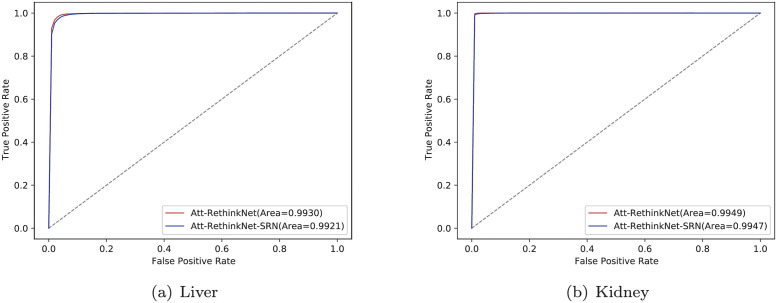
ROC curves of Att-RethinkNet using LSTM and SRN algorithms on liver data (a) and kidney data (b).

The ROCs obtained from the liver and kidney data sets are shown in Fig 6. The results show that the two algorithms have obtained high AUC values on different data sets, and the model based on LSTM is slightly higher than the model based on SRN. Therefore, the selective memory function of LSTM for historical information improves the ability of the model to identify whether a specific drug has potential pathological findings.

In a word, using LSTM neural network as the specific implementation algorithm of RNN layer to complete the early recognition and classification of drug-induced pathological findings obtains more satisfactory performance than the multi-label classification model based on SRN. Therefore, all Att-RethinkNet model mentioned in the follow-up experiments was implemented by using LSTM algorithm in RNN layer.

### Comparison between Att-RethinkNet and RethinkNet

This section aims to implement our proposed Att-RethinkNet and compare it with the baseline RethinkNet framework for drug-induced pathology classification based on gene expression data. For fair comparison, the two models share the same data splitting method and cross validation procedure. The results of the two methods in different organs are shown in Table 4. According to most of the evaluation metrics, it shows that the predictive power of Att-RethinkNet is stronger than that of the RethinkNet. For the rat liver data, the baseline model reached an ACC of 87.2% and an ACC_pair_ of 90.2%, while our proposed model achieved an ACC of 89.4%, an ACC_pair_ of 92.2%, a SEN of 94.2%, a SPE of 98.2% and an AUC of 0.99. In terms of kidney data, Att-RethinkNet has an ACC of 97.5%, an ACC_pair_ of 98.1%, a SEN of 99.1%, a SPE of 99.5% and an AUC of 0.99, which are all higher than the RethinkNet’s results. The reasons why the subset accuracy of kidney data is higher than that of liver data may be that subset accuracy is a rigid measurement, that is, if one element of one sample’s label vector is falsely predicted, the sample is considered falsely predicted. Therefore, high dimensional label vector may be more easily to be falsely predicted. The liver data set has a higher label dimension than that of the kidney, so it is more likely to be judged as a false prediction.

**Table 4:** Comparison between the proposed method and the baseline model.

ORG^1^	CLF^2^	ACC (%)	SEN (%)	SPE (%)	F1 (%)	AUC	ACC_pair_(%)	ACC_avelab_(%)
Liver	RethinkNet	87.2	91.1	98.5	92.3	0.99	90.2	97.4
	Att-RethinkNet	89.4	94.2	98.2	93.8	0.99	92.2	97.8
Kidney	RethinkNet	96.2	98.4	99.5	98.9	0.99	97.5	99.0
	Att-RethinkNet	97.5	99.1	99.5	99.2	0.99	98.1	99.3

^1^ ORG means the data set of target organ.

^2^ CLF means classifier.

**Fig 7:**
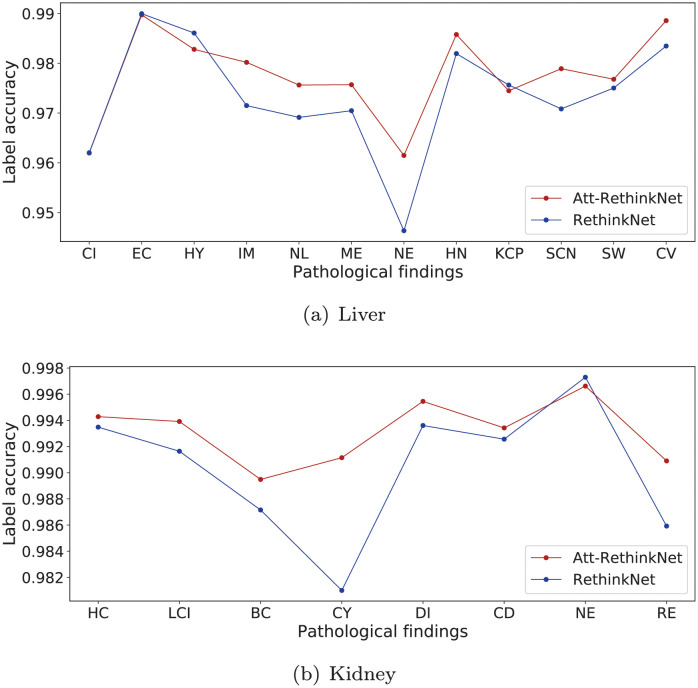
The ACC_lab_ of deep learning experiments in liver data (a) and kidney data (b).

To compare the classification performance on each label, the ACC_lab_ of RethinkNet and Att-RethinkNet is illustrated in Fig 7. The detailed values of ACC_lab_ are summarized in Tables 5 and 6. As expected, obvious improvement of each label’s prediction can be seen for most of the labels with our proposed model, except four findings, cellular infiltration, eosinophilic change, hypertrophy and kupffer cell proliferation in liver, and one finding, necrosis, in kidney. It also shows that eosinophilic change in liver is easier to be recognized compared with other findings for liver and necrosis in kidney is easier to be identified compared with other findings in kidney.

**Table 5:** ACC_lab_ of all pathological findings for RethinkNet and Att-RethinkNet in liver data.

CLF/PF^1^	CI	EC	HY	IM	NL	MI	NE	HN	KCP	SCN	SW	CV
RethinkNet	96.2	99.0	98.6	97.2	96.9	97.0	94.6	98.2	97.6	97.1	97.5	98.3
Att-RethinkNet	96.2	99.0	98.3	98.0	97.6	97.6	96.1	98.6	97.4	97.9	97.7	98.9

^1^ CLF means classifier and PF means pathological finding. Values of columns 2 to 13 indicate the ACC_lab_ (%) of the corresponding pathological finding.

**Table 6:** ACC_lab_ of all pathological findings for RethinkNet and Att-RethinkNet in kidney data.

CLF/PF^1^	HC	LCI	BC	CY	DI	CD	NE	RE
RethinkNet	99.3	99.2	98.7	98.1	99.4	99.3	99.7	98.6
Att-RethinkNet	99.4	99.4	98.9	99.1	99.5	99.3	99.7	99.1

^1^ CLF represents classifier and PF means pathological finding. Values of columns 2 to 9 indicate the ACC_lab_ (%) of the corresponding pathological finding.

**Fig 8:**
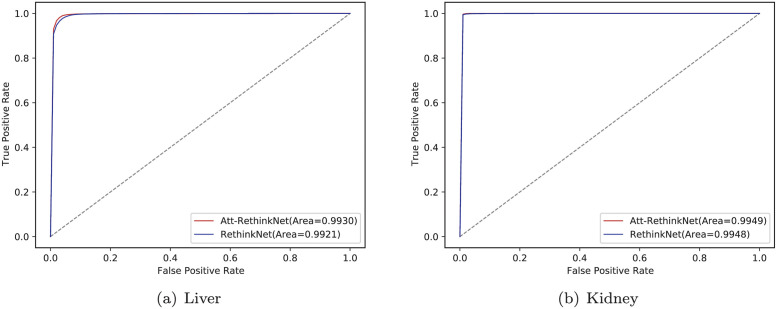
ROC curves of RethinkNet and Att-RethinkNet on liver data (a) and kidney data (b).

The ROCs of both methods are shown in Fig 8. According to the ROCs, Att-RethinkNet has a slightly larger AUC than that of RethinkNet and lies in the left-top of RethinkNet, meaning that our proposed model has a better classification performance than the baseline model.

### Comparison between Att-RethinkNet and the traditional method

Traditional classification algorithms normally reduce the feature dimension and eliminate irrelevant information to optimize the results at the beginning. We applied some feature ranking techniques and found that multi-label F-statistic algorithm show better and more stable accuracy in feature subset. So we calculated the F-statistic score of each gene and picked the *TOP*_*N*_-best performed genes by deleting ranked features gradually. We used a fitness function to evaluate the performance of each feature subset [36]. Since the number of features in the selected subset is significantly smaller than the number of all features, we improved the fitness function by adding an amplification factor λ in order to maximizes the accuracy of classification and minimizes the number of selected genes. In the fitness function, we increased the selected feature subset to λ times to make the fitness value meaningful. The improved fitness function is defined as:

$$\begin{array}{c} \text{Fitness}=\alpha \times \text{ACC}+\left(1-\alpha \right)\times \frac{D_{total}-D_{selected}\times \lambda }{D_{total}} \end{array}$$ (13)

Where ACC is the accuracy. *D*_*total*_ and *D*_*selected*_ represent the size of the total features and the size of the selected features, respectively. *α* is a weight in the range [0, 1], which describes the degree of importance of ACC and *D*_*selected*_. λ is an amplification factor. In our experiments, we tried multiple sets of parameters and finally set *α* = 0.6 and λ = 10 which had the highest ACC.

**Fig 9:**
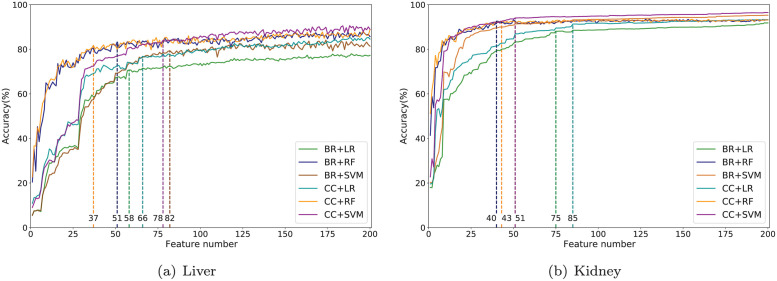
Intermediate results of selecting the optimal feature subset for BR and CC. The numbers of selected features are marked with dashed lines.

In this paper, we applied the improved fitness function to seek an optimal subset of relevant features. The intermediate results of selecting the optimal feature subset are presented in Fig 9, where x-axis shows the number of selected features that were used for machine learning model construction and y-axis shows the subset accuracy when classifying unknown samples using the selected feature sets. Here we combined the BR/CC with LR, RF and SVM, which are all popular classifiers in relevant areas [37–41].

From Fig 9, for liver data, BR based on SVM selected the most features, and CC based on RF selected the least features, approximately only one-half of the other methods. CC based on SVM achieved the highest accuracy. For feature selection in kidney data, BR and CC methods based on SVM selected the same number of genes and CC based SVM achieved the highest accuracy.

We further show the classification comparison of BR, CC and Att-RethinkNet in Table 7. From Table 7, we found that the prediction accuracy of traditional machine learning-based models for liver data ranges from 69.86% to 83.71% and for kidney data, 88.74% to 94.66%. Among these methods, it can be seen that BR using LR as base classifier has the lowest accuracy on both data sets. BR and CC, with RF as the base classifiers, gained the best AUCs in all data sets. In CC approach, when SVM was used as a base classifier, the classifiers always come with the highest accuracy score, 83.71% in liver and 94.66% in kidney respectively. In general, all these machine learning-based methods retain considerable small feature subsets and the result on kidney data is better than that on the liver data. Besides, when using the same base classifiers, CC outperforms BR in most times because CC takes label correlations into account. In terms of the Att-RethinkNet, the Att-RethinkNet achieves the highest ACC, ACC_pair_ and ACC_avelab_ for both liver and kidney data. One important reason is that our model not only considers label correlations but also applies proper weights to both labels and features, and solves the issue caused by label order as well.

**Table 7:** The performance of BR, CC and Att-RethinkNet on liver and kidney data.

ORG^1^	CLF^2^	BCLF^3^	FN^4^	ACC (%)	SEN (%)	SPE (%)	F1 (%)	AUC	ACC_pair_ (%)	ACC_avelab_ (%)
Liver	BR	LR	58	69.9	85.3	96.5	87.3	0.98	78.3	95.5
	BR	RF	51	81.5	92.0	98.3	95.0	0.99	87.2	98.0
	BR	SVM	82	78.7	88.7	97.0	89.5	0.99	84.1	96.5
	CC	LR	66	76.0	84.0	96.4	85.4	0.96	79.2	95.0
	CC	RF	37	79.6	89.6	98.2	93.4	0.99	85.2	97.6
	CC	SVM	78	83.7	88.0	97.0	88.5	0.97	85.5	96.1
	Att-RethinkNet	-	-	89.4	94.2	98.2	93.8	0.99	92.2	97.8
Kidney	BR	LR	75	88.7	97.4	95.6	96.3	0.99	92.1	96.6
	BR	RF	40	92.6	99.5	97.0	98.2	0.99	93.9	98.3
	BR	SVM	51	91.8	98.9	96.5	97.5	0.99	93.9	97.7
	CC	LR	85	91.8	98.5	96.2	97.2	0.99	92.9	97.4
	CC	RF	43	92.0	99.2	97.4	98.2	0.99	93.9	98.3
	CC	SVM	51	94.7	99.4	97.4	98.2	0.99	95.5	98.3
	Att-RethinkNet	-	-	97.5	99.1	99.5	99.2	0.99	98.1	99.3

^1^ ORG means the data of target organ.

^2^ CLF means classifier.

^3^ BCLF means base classifier.

^4^ FN means selected feature subset size.

**Fig 10:**
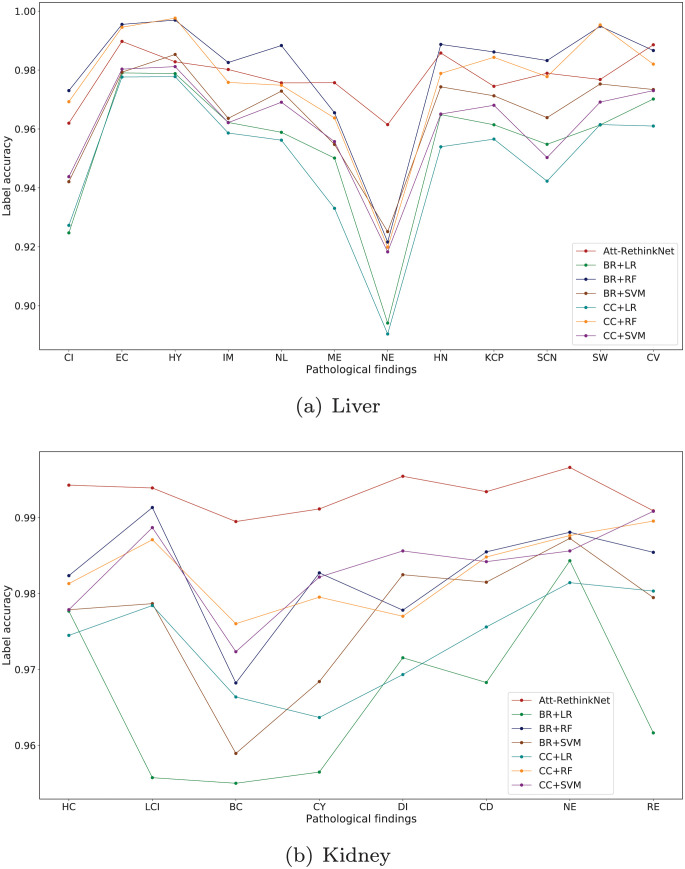
Accuracy of each label of Att-RethinkNet and the traditional machine learning-based methods in liver (a) and kidney (b).

Furthermore, the detailed ACC_lab_ of each label for the Att-RethinkNet and these machine learning-based models are shown in Fig 10, Tables 8 and 9. For liver pathological findings, the Att-RethinkNet maintains a high value on average compared with other methods. For kidney pathological findings, the ACC_lab_ of each label of Att-RethinkNet is the highest in comparison with all other referring traditional classification methods.

**Table 8:** Performance of all pathological findings for BR, CC and Att-RethinkNet in liver data.

CLF/PF^1^	BCLF^2^	CI	EC	HY	IM	NL	MI	NE	HN	KCP	SCN	SW	CV
BR	LR	92.5	97.9	97.9	96.2	95.9	95.0	89.4	96.5	96.1	95.5	96.1	97.0
BR	RF	97.3	99.6	99.7	98.3	98.8	96.5	92.2	98.9	98.6	98.3	99.5	98.7
BR	SVM	94.2	97.9	98.5	96.4	97.3	95.5	92.5	97.4	97.1	96.4	97.5	97.3
CC	LR	92.7	97.8	97.8	95.9	95.6	93.3	89.0	95.4	95.7	94.2	96.1	96.1
CC	RF	96.9	99.5	99.8	97.6	97.5	96.4	92.0	97.9	98.4	97.8	99.5	98.2
CC	SVM	94.4	98.0	98.1	96.2	96.9	95.6	91.8	96.5	96.8	95.0	96.9	97.3
Att-RethinkNet	-	96.2	99.0	98.3	98.0	97.6	97.6	96.1	98.6	97.4	97.9	97.7	98.9

^1^ CLF represents classifier and PF means pathological finding.

^2^ BCLF means base classifier. Columns 3 to 14 indicate the ACC_lab_ (%) of the corresponding pathological finding.

**Table 9:** Accuracy of all pathological findings for established deep learning models in kidney data.

CLF/PF^1^	BCLF^2^	HC	LCI	BC	CY	DI	CD	NE	RE
BR	LR	97.8	95.6	95.5	95.6	97.2	96.8	98.4	96.2
BR	RF	98.2	99.1	96.8	98.3	97.8	98.5	98.8	98.5
BR	SVM	97.8	97.9	95.9	96.8	98.2	98.1	98.7	97.9
CC	LR	97.4	97.8	96.6	96.4	96.9	97.6	98.1	98.0
CC	RF	98.1	98.7	97.6	98.0	97.7	98.5	98.8	99.0
CC	SVM	97.8	98.9	97.2	98.2	98.6	98.4	98.6	99.1
Att-RethinkNet	-	99.4	99.4	98.9	99.1	99.5	99.3	99.7	99.1

^1^ CLF represents classifier and PF means pathological finding.

^2^ BCLF means base classifier. Columns 3 to 10 indicate the ACC_lab_ (%) of the corresponding pathological finding.

**Fig 11:**
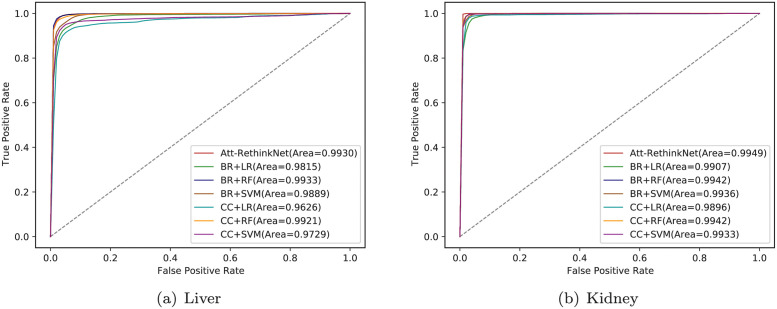
The comparison of ROC curves between the proposed method and some commonly used machine learning-based models. (a) shows the liver data and (b) shows the kidney data. In the plot, BR plus LR represents BR model that uses LR as base classifier. Other symbols are defined similarly.

We show the ROC curves of Att-RethinkNet and all the traditional models in Fig 11. We obtain the highest AUC values of the AttRethinkNet among all the methods.

### Comparison between Att-RethinkNet and the integrative model

We also compared the proposed approach with a method, that we called “integrative model” in our study [22]. This model was also developed for drug-induced pathological finding prediction. Different from our method, which builds a multi-label prediction model, this model trains a model for each pathological finding and combined all the pathology prediction models.

We trained the presented integrative model of 5-nearest neighbor classifiers. The pathology similarities matrix that describes co-occurrences of two pathological findings within training set of each fold were reported in S4 Fig. Tables 10, 11 and 12 list the performance of the integrative model and the proposed drug toxicity prediction model. From the tables, we can see that although the integrative model has obtained considerably high classification accuracy when classifying each label (although lower than the proposed method, shown in Tables 11 and 12), the subset accuracy of the integrative model is unsatisfactory (Table 10). The low subset accuracy is due to the fact that the integrative model makes predictions for each label separately, which cannot guarantee the prediction result for each pathology correct at the same time.

**Table 10:** The performance of the integrative model and Att-RethinkNet.

ORG^1^	CLF^2^	ACC (%)	SEN (%)	SPE (%)	F1 (%)	AUC	ACC_pair_ (%)	ACC_avelab_ (%)
Liver	Integra	50.3	99.3	89.8	84.9	0.50	83.0	92.6
	Att	89.4	94.2	98.2	93.8	0.99	92.2	97.8
Kidney	Integra	28.5	100.0	41.0	74.2	0.50	63.8	68.4
	Att	97.5	99.1	99.5	99.2	0.99	98.1	99.3

^1^ ORG means the data set of target organ.

^2^ CLF means classifier. Integra means integrative model. Att means Att-RethinkNet.

**Table 11:** ACC_lab_ of the integrative model and Att-RethinkNet for liver data.

CLF^1^	CI	EC	HY	IM	NL	MI	NE	HN	KCP	SCN	SW	CV
Integrative model	83.3	95.8	90.1	98.5	88.2	86.6	91.9	90.0	95.0	97.9	95.0	98.7
Att-RethinkNet	96.2	99.0	98.3	98.0	97.6	97.6	96.1	98.6	97.4	97.9	97.7	98.9

^1^ CLF means classifier. Columns 2 to 13 indicate the ACC_lab_ (%) of the corresponding pathological finding.

**Table 12:** ACC_lab_ of the integrative model and Att-RethinkNet for kidney data.

CLF^1^	HC	LCI	BC	CY	DI	CD	NE	RE
Integrative model	56.8	68.0	79.1	64.7	73.4	62.2	76.8	66.0
Att-RethinkNet	99.4	99.4	98.9	99.1	99.5	99.3	99.7	99.1

^1^ CLF represents classifier. Columns 2 to 9 indicate the ACC_lab_ (%) of the corresponding pathological finding.

**Fig 12:**
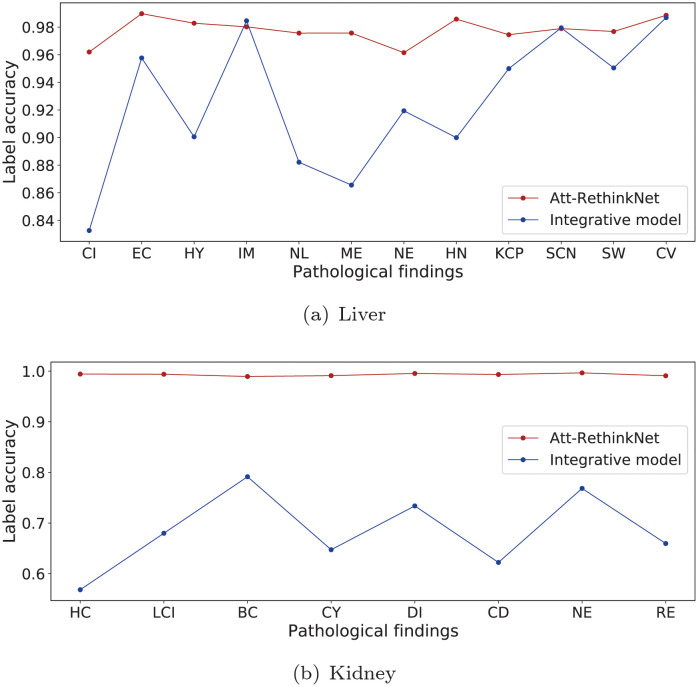
ACC_lab_ comparison of the proposed model with the integrative model using liver samples (a) and kidney samples (b).

In terms of predicting each label, we specifically show the ACC_lab_ of the proposed model and the integrative model in Fig 12. The results prove that the proposed model has a significant improvement in correctly predicting each pathology when compared with the integrative method. The difference of ACC_lab_ between our method and the integrative method ranges from around 1% to around 12% for drug-induced liver toxicity except increased mitosis(IM) and single cell necrosis(SCN), while the ACC_lab_ difference ranges from 20% to 42% for drug-induced kidney toxicity.

**Fig 13:**
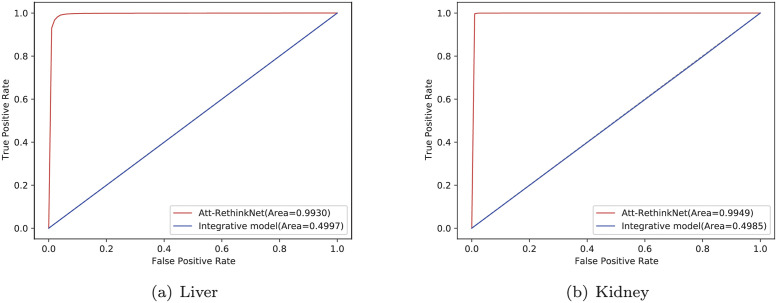
The ROC curves of the Att-RethinkNet and the integrative method. (a) is for liver data and (b) is for kidney data.

The ROC curves of Att-RethinkNet and the integrative model can be found in Fig 13. From the experimental results, we can find that the curve of Att-RethinkNet lies far above that of the integrative model. The AUC of the integrative model is approximately half of that of the Att-RethinkNet.

### Validation on rat liver *in vitro* data

In order to further verify the reliability of the prediction model, we carried out experiments on independent and invisible data, the *in vitro* toxicity data of rat liver. We divided the data into two parts. One part of data was used as the training set and it was augmented and balanced, and the remaining data was used as the test set. We selected the corresponding gene expression levels of rat liver *in vitro* at the time point of 24 hours after a single dose administration. Pre-processing operations including dose-response curve fitting was operated on this data.

Table 13 lists the classification results on liver *in vitro* data set. For liver pathology, our Att-RethinkNet model based on LSTM achieved relatively high accuracy, with a value of 73.0%. The SEN and SPE are all above 80%. And the SPE achieves 94.8% which is 10% higher than the SEN. Therefore, our approach can be used in the very first step of toxicity evaluation. Drugs are determined safe with high accuracy by predicting them without any pathological findings. However, if the results show that the drug can induce a certain pathological finding, further safety screening may still be needed. The experimental results show that our proposed model is applicable to new drugs and it is able to reduce the over-fitting, and make predictions for the invisible test data.

**Table 13:** Classification results on the liver *in vitro* data set.

ORG^1^	CLF^2^	ACC (%)	SEN (%)	SPE (%)	F1 (%)	AUC	ACC_pair_(%)	ACC_avelab_(%)
Liver	Att-RethinkNet	73.0	84.3	94.8	84.8	0.90	80.1	89.5

^1^ ORG means the dataset of target organ.

^2^ CLF means classifier.

Table 14 further shows the performance of the model on each label. It can be clearly seen that our proposed model has the ability to predict the specific pathological findings corresponding to 12 drug-induced liver toxicity, and can achieve high accuracy in predicting the pathology finding hepatodiaphragmatic nodule, while the prediction ability of pathology finding necrosis needs to be improved. In general, Att-RethinkNet can provide auxiliary functions for the process of drug development.

**Table 14:** ACC_lab_ of Att-RethinkNet for liver *in vitro* data.

CLF^1^	CI	EC	HY	IM	NL	MI	NE	HN	KCP	SCN	SW	CV
Att-RethinkNet	84.1	92.9	85.1	94.7	91.2	89.9	69.7	99.0	88.4	91.0	94.8	92.9

^1^ CLF means classifier. Columns 2 to 13 indicate the ACC_lab_ (%) of the corresponding pathological finding.

**Fig 14:**
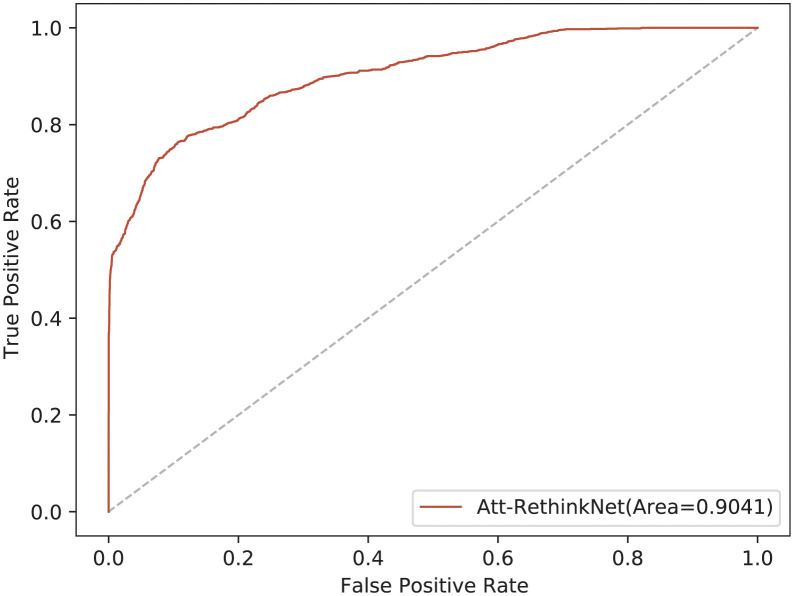
The ROC curves of the Att-RethinkNet for liver *in vitro* data.

The ROCs of the results are shown in Fig 14. It can be seen from the ROCs that the area under the curve of the proposed model is 0.9, which shows that the model can predict the pathological findings on invisible and independent data.

In conclusion, our proposed model is generalized, which is able to predict toxic pathological findings of unknown drugs. The subset accuracy of each fold and standard deviation of prediction results corresponding to the above cross-validation experiments are put in S2 Table, and the parameter settings of the proposed deep neural network model are put in S3 Table.

## Conclusion and discussion

Our proposed Att-RethinkNet framework can achieve excellent performance in predicting drug-induced pathology in multiple organs based on toxicogenomics data, which is helpful to detect and diagnose organ-specific toxicity in early stage, and provides a cost effective solution for drug industry.

Our study proposed an attention-based RethinkNet framework for drug-induced pathological finding prediction based on gene expression profile. This model mimics the human rethinking procedure, and the attention mechanism before LSTM layer focuses on more important features. Our model has shown impressive performance on both liver and kidney. The accuracy of the proposed Att-RethinkNet model is 89.4% for *in vivo* liver data and is 97.5% for *in vivo* kidney data, higher than those of the BR, CC, RethinkNet, and the “integrative model”. The running time of Att-RethinkNet only takes a few hours, which is much faster than the traditional method BR and CC which takes nearly a week.

In the current work, we find that the classification results are already good enough to show the effectiveness of the proposed Att-RethinkNet. However, our experiment still has several limitations. For example, pathological findings relies on manual labeling. When the collection of labels or the number of drugs is large, the definition of labels corresponding to each instance can take a lot of time. Moreover, we only targeted on liver and kidney without additional target organs due to the difficulty in obtaining gene expression data and pathological information, so there is no verification on other kinds of organs in our study.

For our proposed deep neural network model, there is still room for improvement and scope for expansion. Next work, we will consider the analysis of gene selection [42], our network model can be optimized to identify genes highly associated with potential toxicity. In fact, our model is not limited to liver and kidney and can be easily extended to other organs. In the future, we aim to construct a more generalized model which is suitable for all organs and incorporate multi-omics data.

Additionally, the proposed method provides a new and interesting insight to multi-label classification problems, which is applicable to a spectrum of domains, such as sound classification, image classification, and text categorization. It would be an interesting future work to explore the application scope of our multi-label classification model in the field of bioinformatics, such as prediction of compound-protein interactions [43], identification of human protein subcellular localization for understanding protein functions [44], diagnosing cervical cancer at early stages based on multiple risk factors [45].

## Supporting information

S1 Text The improved MLSMOTE (Multilabel Synthetic Minority Over-sampling Technique) algorithm which effectively handles the imbalanced data set for multi label classification. (PDF)

S1 Table The drugs or chemical compounds involved in the experimental data. (PDF)

S2 Table The standard deviation of all the results. (PDF)

S3 Table The parameter setting of the developed model. (PDF)

S1 Fig The visualization of all targeted pathological findings using t-SNE for liver. (PDF)

S2 Fig The visualization of all targeted pathological findings using t-SNE for kidney. (PDF)

S3 Fig The confusion matrix of the pathology classification of other folds for both liver and kidney. (PDF)

S4 Fig The pathology similarities matrix that describes co-occurrences of two pathological findings within training set of each fold. (PDF)
