# Supplementary material for: A multi-label learning model for predicting drug-induced pathology in multi-organ based on toxicogenomics data
Source: PLoS Comput Biol. 2022 Sep 7;18(9):e1010402. doi: 10.1371/journal.pcbi.1010402 (PMC9451100; doi:10.1371/journal.pcbi.1010402)
Supplement: S1 Text — (PDF) [file pcbi.1010402.s001.pdf]

MLSMOTE

- ## STEPS

The steps in improved MLSMOTE algorithm can be summarized as follows:

(1) Select minority instances. After selecting the data set that needs to be balanced, our first task is to select the reference instance around which the synthetic instance is to be generated. If a label l satisfies the inequality equation, $IRPL(l) > MeanIR$, it is considered a minority, through which the set of labels can be partitioned into two subsets, majority labels and minority labels. All samples containing minority labels are considered as minority instances, and each of them will be applied to K-nearest neighbor search.

(2) Choose an instance in minority instances and find its k nearest neighbors. In this step, one instance with minority labels is selected as reference point, then calculate the Euclidean distance and get the set of its nearest neighbors called neighbour points.

(3) Synthesis new data. The synthetic procedure consists of two parts: feature vector generation and label set generation. Randomly choose one of neighbour data points and draw a line between it and reference point. Our method of synthetic feature value is the same as SMOTE, while the approach to synthesizing new labels depends on the circumstances. If all the labels in reference point and in neighbour point are totally the same, we duplicate the label set. If the number of the same corresponding labels is not less than 1, we adopt a method, creating new data along the line, which is similar to feature generation to create new label values, and set a threshold of 0.5, values less than or equal to the threshold of 0.5 are assigned to class 0 and values greater than 0.5 are assigned to class 1. If the label correlation information of two points is completely different, we synthetic data based on the union approach mentioned in basic algorithm, which means all the labels that appear in the reference instance or any of its neighbour instances will be in the synthetic label set.

(4) Repeat the process until termination condition. The procedure is repeated enough times until the current iteration reaches max times or the value of MaxIR is below criterion rate.

- FORMULAS

The calculation formula involved in the MLSMOTE algorithm:

In multi label dataset, imbalance ratio (IR), the ratio of the number of samples in majority label to the number of samples in minority label, is used for measuring the degree of imbalance. IRPL is an abbreviation for imbalance ratio per label, which represents the maximum value of label sample number versus the current label sample number. MeanIR, an abbreviation for mean imbalance ratio, represents the average rate of IRPL of all the labels. Max imbalance ratio, also called MaxIR, denotes the greatest value of IRPL. All math formulas concerning IR are defined below.

$$IRPL(l) = \frac{arg\max_{l^*=L_1}^{L_{|L|}}\left(\sum_{i=1}^{|D|}\Phi(l^*,Y_i)\right)}{\sum_{i=1}^{|D|}\Phi(l,Y_i)} \qquad \Phi(l,Y_i) = \begin{cases} 1 & l \in Y_i \\ 0 & l \notin Y_i \end{cases}$$

$$MeanIR = \frac{1}{|L|}\sum_{l=L_1}^{L_{|L|}}(IRPL(l))$$

$$MaxIR = \max\left(\frac{1}{|L|}\sum_{l=L_1}^{L_{|L|}}(IRPL(l))\right)$$

Where D represents the dataset, L represents all labels, L_l stands for the lth label in the label set, and Y_i for the label of ith instance in dataset D.

- RESULTS

Here, we presented the results of metrics that measure the imbalance ratio of data set before and after the MLSMOTE, and indicated the number of samples liver or kidney had in the majority and minority classes of original data.

After data augmentation, we obtained 16,460 samples and 16,268 samples for liver and kidney respectively. To better understand the degree of data imbalance in the original data, we show the number of samples corresponding to the presence of specific pathological findings in two organ data sets. For original liver data set, Table A in S1 Text shows the number of samples with this pathological finding in each label, and the corresponding imbalance ratio per label (IRPL). For original kidney data set, Table B in S1 Text shows the number of samples with this pathological finding in each label, and the corresponding imbalance ratio per label (IRPL). Table C in S1 Text shows the majority and minority classes in liver and kidney before the MLSMOTE.

Table A in S1 Text. The number of samples corresponding to the presence of specific pathological findings in liver.

ME/PF[1]	CI	EC	HY	IM	NL	MI	NE	HN	KCP	SCN	SW	CV
NUM[2]	25	15	12	26	5	25	71	6	6	20	5	15
IRPL[3]	2.84	4.73	5.91	2.73	14.20	2.84	1.00	11.83	11.83	3.55	14.20	4.73

[1]ME means the results of metrics and PF means pathological finding. [2]NUM means the number of samples with specific pathological finding. [3]IRPL is a measure of the MLSMOTE algorithm, imbalance ratio per label. Columns 2 to 13 indicate the results of pathological finding..

Table B in S1 Text. The number of samples corresponding to the presence of specific pathological findings in kidney.

ME/PF[1]	HC	LCI	BC	CY	DI	CD	NE	RE
NUM[2]	9	5	8	7	17	12	7	12
IRPL[3]	1.89	3.40	2.13	2.43	1.00	1.42	2.43	1.42

[1]ME means the results of metrics and PF means pathological finding. [2]NUM means the number of samples with specific pathological finding. [3]IRPL is a measure of the MLSMOTE algorithm, imbalance ratio per label. Columns 2 to 9 indicate the results of pathological finding..

Table C in S1 Text. shows the majority and minority classes in liver and kidney before the MLSMOTE.

	Majority classes	Minority classes
Liver	CI, EC, HY, IM, MI, NE, SCN, CV	NL, HN, KCP, SW
Kidney	HC, DI, CD, RE	LCI, BC, CY, NE

Furthermore, Table D in S1 Text illustrates the change in imbalance ratio (IR) before and after augmentation of the dataset by MLSMOTE. Two metrics, MaxIR and MeanIR, were selected for analysis on the liver and kidney datasets, and their rates of change relative to the original data were calculated, expressed as %△. It is not difficult to see from Table D in S1 Text that in the two datasets, both MaxIR and MeanIR show a relatively significant decrease, which means that the sample imbalance ratio between the pathological findings with the highest frequency and the pathological findings with the lowest frequency has been improved to a greater extent.

Table D in S1 Text. The change in imbalance ratio (IR) before and after augmentation of the dataset by MLSMOTE.

ORG[1]/ME[4]	MaxIR			MeanIR		
	Before[2]	After[3]	%△	Before[2]	After[3]	%Δ
Liver	14.20	8.70	-38.72	6.70	3.88	-42.11
Kidney	3.40	1.76	-48.14	2.01	1.37	-32.15

[1]ORG means the dataset of target organ. [2]Before means the change in imbalance ratio before augmentation of the dataset by MLSMOTE. [3]After means the change in imbalance ratio after augmentation of the dataset by MLSMOTE. [4]ME means the results of metrics.

- ## ADVANTAGES

In the unbalanced data set, the category labels concerned by people may only occupy a small part of the data, that is, the number of positive samples with certain toxic effects of drugs may be relatively small. If the training data is directly used for modeling, the satisfactory effect will not be obtained due to the scarcity of effective information. Researchers usually use the sampling method to change the unbalanced sample distribution and get a relatively balanced sample set. The basic idea is divided into two categories: one is the undersampling realized by eliminating the categories with more samples, and the other is to add the oversampling of a few samples to the data set. However, the sampling method of under sampling may delete the samples that are helpful to the performance of the classification model and lose a lot of important information. In oversampling, the random return sampling method of minority samples is easy to lead to serious over fitting, and the clinical experimental data can not be collected anytime and anywhere, so it is difficult to directly add new minority samples. Therefore, it is necessary to amplify the data by artificial synthesis in order to balance the data categories. At present, the mainstream method is to use SMOTE technology (Synthetic Minority Oversampling Technique) to interpolate between minority samples to synthesize new minority samples.

Different from the one-to-one relationship between labels and samples in SMOTE, in the multi-label classification task, a sample may contain labels with high frequency and labels with low frequency. According to the definition of multi-label classification, the labels that are positive in most samples can be called majority classes, and the labels with low frequency in samples can be called minority classes. In order to solve the problem of data imbalance in the field of multi-label classification, MLSMOTE technique (Multilabel Synthetic Minority Over-sampling Technique), which is an extension of the traditional SMOTE method, is proposed to deal with multi-dimensional label vectors, which can not only overcome the problem of loss of samples of most classes, but also prevent over fitting caused by simply copying samples of a few classes.
