# Supplementary material for: A multi-label learning model for predicting drug-induced pathology in multi-organ based on toxicogenomics data
Source: PLoS Comput Biol. 2022 Sep 7;18(9):e1010402. doi: 10.1371/journal.pcbi.1010402 (PMC9451100; doi:10.1371/journal.pcbi.1010402)
Supplement: S1 Table — (PDF) [file pcbi.1010402.s002.pdf]

Table A in S1 Table. The drugs or chemical compounds involved in the experimental data

Liver	Kidney	Overlapping between two datasets
2,4-dinitrophenol	acetaminophen	acetaminophen
2-nitrofluorene	acetazolamide	acetazolamide
3-methylcholanthrene	allopurinol	allopurinol
LPS	allyl alcohol	allyl alcohol
N-methyl-N-nitrosourea	amphotericin B	amphotericin B
N-nitrosomorpholine	bromobenzene	bromobenzene
TNFalpha	bromoethylamine	bromoethylamine
WY-14643	bucetin	bucetin
acarbose	caffeine	caffeine
acetamidofluorene	captopril	captopril
acetaminophen	carboplatin	carboplatin
acetazolamide	cephalothin	cephalothin
adapin	ciprofloxacin	ciprofloxacin
aflatoxin B1	cisplatin	cisplatin
ajmaline	clofibrate	clofibrate
allopurinol	cyclophosphamide	cyclophosphamide
allyl alcohol	cyclosporine A	cyclosporine A
amiodarone	desmopressin acetate	desmopressin acetate
amitriptyline	doxorubicin	doxorubicin
amphotericin B	enalapril	enalapril
aspirin	erythromycin ethylsuccinate	erythromycin ethylsuccinate
azathioprine	ethinylestradiol	ethinylestradiol
bendazac	ethionine	ethionine
benzbromarone	gentamicin	gentamicin
benziodarone	hexachlorobenzene	hexachlorobenzene
bortezomib	imipramine	imipramine
bromobenzene	indomethacin	indomethacin
bromoethylamine	ketoconazole	ketoconazole
bucetin	lomustine	lomustine
buthionine sulfoximine	methyltestosterone	methyltestosterone
butylated hydroxyanisole	monocrotaline	monocrotaline
caffeine	nitrofurantoin	nitrofurantoin
captopril	omeprazole	omeprazole
carbamazepine	phenacetin	phenacetin
carbon tetrachloride	phenylanthranilic acid	phenylanthranilic acid
carboplatin	phenylbutazone	phenylbutazone
cephalothin	puromycin aminonucleoside	puromycin aminonucleoside
chloramphenicol	rifampicin	rifampicin
chlormadinone	thioacetamide	thioacetamide
chlormezanone	triamterene	triamterene
chlorpheniramine	valproic acid	valproic acid
chlorpromazine		
chlorpropamide		
cimetidine		
ciprofloxacin		
cisplatin		
clofibrate		
clomipramine		
colchicine		
coumarin		
cycloheximide		
cyclophosphamide		

cyclosporine A
danazol
dantrolene
desmopressin acetate
dexamethasone
diazepam
diclofenac
diethyl maleate
diltiazem
disopyramide
disulfiram
doxorubicin
enalapril
erythromycin ethylsuccinate
ethambutol
ethanol
ethinylestradiol
ethionamide
ethionine
etoposide
famotidine
fenofibrate
fluoxetine hydrochloride
fluphenazine
flutamide
furosemide
galactosamine
gefitinib
gemfibrozil
gentamicin
glibenclamide
griseofulvin
haloperidol
hexachlorobenzene
hydroxyzine
ibuprofen
imatinib, methanesulfonate salt
imipramine
indomethacin
iproniazid
isoniazid
ketoconazole
labetalol
lomustine
lornoxicam
mefenamic acid
meloxicam
metformin
methapyrilene
methimazole
methyldopa
methylene dianiline
methyltestosterone
mexiletine

monocrotaline
moxisylyte
naphthyl isothiocyanate
naproxen
nicotinic acid
nifedipine
nimesulide
nitrofurantoin
nitrofurazone
nitrosodiethylamine
omeprazole
papaverine
pemoline
penicillamine
perhexiline
phalloidin
phenacetin
phenobarbital
phenylanthranilic acid
phenylbutazone
phenytoin
phorone
promethazine
propranolol
propylthiouracil
puromycin aminonucleoside
quinidine
ranitidine
rifampicin
rosiglitazone maleate
rotenone
simvastatin
sulfasalazine
sulindac
sulpiride
tacrine
tamoxifen
tannic acid
terbinafine
tetracycline
theophylline
thioacetamide
thioridazine
ticlopidine
tiopronin
tolbutamide
triamterene
triazolam
trimethadione
tunicamycin
valproic acid
vitamin A
