# Supplementary material for: A multi-label learning model for predicting drug-induced pathology in multi-organ based on toxicogenomics data
Source: PLoS Comput Biol. 2022 Sep 7;18(9):e1010402. doi: 10.1371/journal.pcbi.1010402 (PMC9451100; doi:10.1371/journal.pcbi.1010402)
Supplement: S2 Table — (PDF) [file pcbi.1010402.s003.pdf]

Table A in S2 Table. Test set subset accuracy (ACC) and corresponding mean, standard deviation, variance

Organ	Classifier	Test1(ACC)	Test2(ACC)	Test3(ACC)	Test4(ACC)	Test5(ACC)	Std(Standard Deviation)	Variance	Average
Liver	BR+LR	0.702612394	0.714459295	0.69835966	0.702308627	0.67527339	0.012849583	0.0143663	0.698602673
	CC+LR	0.758809235	0.771567436	0.743924666	0.77764277	0.749696233	0.012732138	0.014235	0.760328068
	BR+RF	0.812879708	0.816221142	0.809538275	0.816828676	0.817436209	0.002973812	0.0033248	0.814580802
	CC+RF	0.780680437	0.796780073	0.807108141	0.793742406	0.803766707	0.009200471	0.0102864	0.796415553
	BR+SVM	0.802247874	0.788882139	0.806196841	0.772782503	0.765492102	0.015932441	0.017813	0.787120292
	CC+SVM	0.845686513	0.846597813	0.831409478	0.837788578	0.82381531	0.008640227	0.0096601	0.837059538
Kidney	BR+LR	0.888137677	0.880454825	0.901352182	0.878573624	0.888718106	0.008038335	0.0089871	0.887447283
	CC+LR	0.914259373	0.913337431	0.927473878	0.915155241	0.918844144	0.00517916	0.0057905	0.917814013
	BR+RF	0.919176398	0.932390904	0.934542102	0.918229327	0.924992315	0.006658558	0.0074445	0.925866209
	CC+RF	0.919791026	0.92685925	0.933312846	0.907470028	0.913618199	0.009185112	0.0102693	0.92021027
	BR+SVM	0.916717886	0.914874001	0.932390904	0.908392253	0.915770058	0.007938603	0.0088756	0.91762902
	CC+SVM	0.947141979	0.946834665	0.954824831	0.940055334	0.944359053	0.004813353	0.0053815	0.946643172
Liver	Att-RethinkNet(LSTM)	0.905224787	0.891555286	0.893681652	0.887302552	0.893985419	0.005938309	0.0066392	0.894349939
	Att-RethinkNet(SRN)	0.866950182	0.882746051	0.858748481	0.90036452	0.868772783	0.014626801	0.0163533	0.875516403
	RethinkNet(LSTM)	0.859356015	0.883049818	0.845686513	0.874240583	0.896415553	0.017749336	0.0198444	0.871749696
Kidney	Att-RethinkNet(LSTM)	0.978180701	0.973878304	0.97664413	0.975714725	0.969873962	0.002855346	0.0031924	0.974858364
	Att-RethinkNet(SRN)	0.979102643	0.947141979	0.944990781	0.979403627	0.971103597	0.015236526	0.017035	0.964348525
	RethinkNet(LSTM)	0.977566073	0.940381069	0.940995698	0.971718414	0.981248079	0.017972582	0.020094	0.962381866
Liver	Integrative model	0.52764277	0.515795869	0.490583232	0.486026731	0.494532199	0.016030826	0.017923	0.50291616
Kidney	Integrative model	0.293177628	0.292562999	0.282728949	0.28127882	0.277589917	0.00627524	0.0070159	0.285467662
