# Supplementary material for: A multi-label learning model for predicting drug-induced pathology in multi-organ based on toxicogenomics data
Source: PLoS Comput Biol. 2022 Sep 7;18(9):e1010402. doi: 10.1371/journal.pcbi.1010402 (PMC9451100; doi:10.1371/journal.pcbi.1010402)
Supplement: S3 Table — (PDF) [file pcbi.1010402.s004.pdf]

Table A in S3 Table. Parameter settings of proposed model

ORG	MODEL	OPT	LR	Epoch	BS	L2	$ActFunc_{Att\text{-}act}$	$ActFunc_{DENSE1}$	$Dropout_{RNN}$	$ActFunc_{RNN}$	$ActFunc_{DENSE2}$
Liver	Att-RethinkNet	Adam	0.001	368	256	10^{-6}	softmax	relu	0.1	sigmoid	sigmoid
Kidney	Att-RethinkNet	Adam	0.001	151	256	10^{-5}	softmax	relu	0.1	sigmoid	sigmoid

ORG means target organ. OPT represents optimizer. LR represents the initial learning rate. BS represents batch size. L2 represents l2 regularization. $ActFunc_{Att\text{-}act}$ refers to the activation function used by the activation layer in the attention block. $Dropout_{RNN}$ and $ActFunc_{RNN}$ are parameters of the RNN layer, respectively representing recurrent dropout and activation function. $ActFunc_{DENSE1}$ and $ActFunc_{DENSE2}$ represent the dense layer before and after the RNN layer respectively. Other hyperparameters are indicated in the code of GitHub https://github.com/RanSuLab/Drug-Toxicity-Prediction-MultiLabel.
