# Supplementary material for: A multi-label learning model for predicting drug-induced pathology in multi-organ based on toxicogenomics data
Source: PLoS Comput Biol. 2022 Sep 7;18(9):e1010402. doi: 10.1371/journal.pcbi.1010402 (PMC9451100; doi:10.1371/journal.pcbi.1010402)
Supplement: S1 Fig — (PDF) [file pcbi.1010402.s005.pdf]

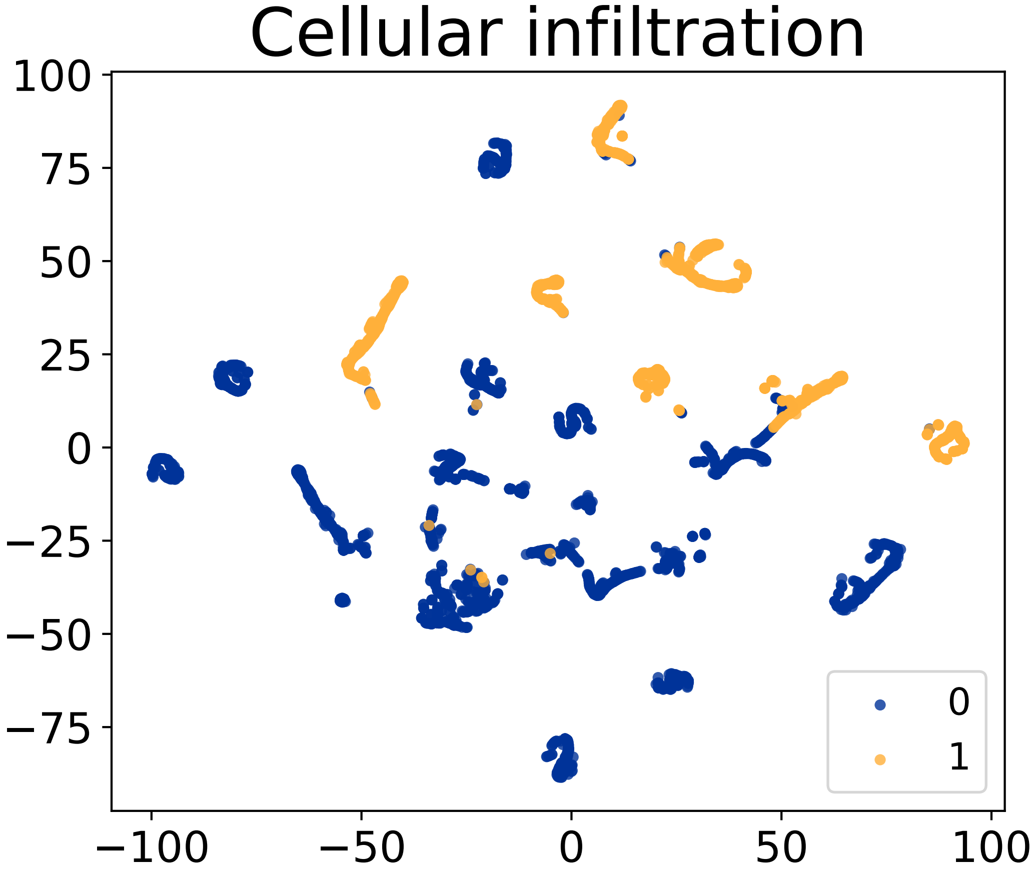

S1 Fig (a): The t-SNE visualization of cellular infiltration features generated after RNN layer on liver data.

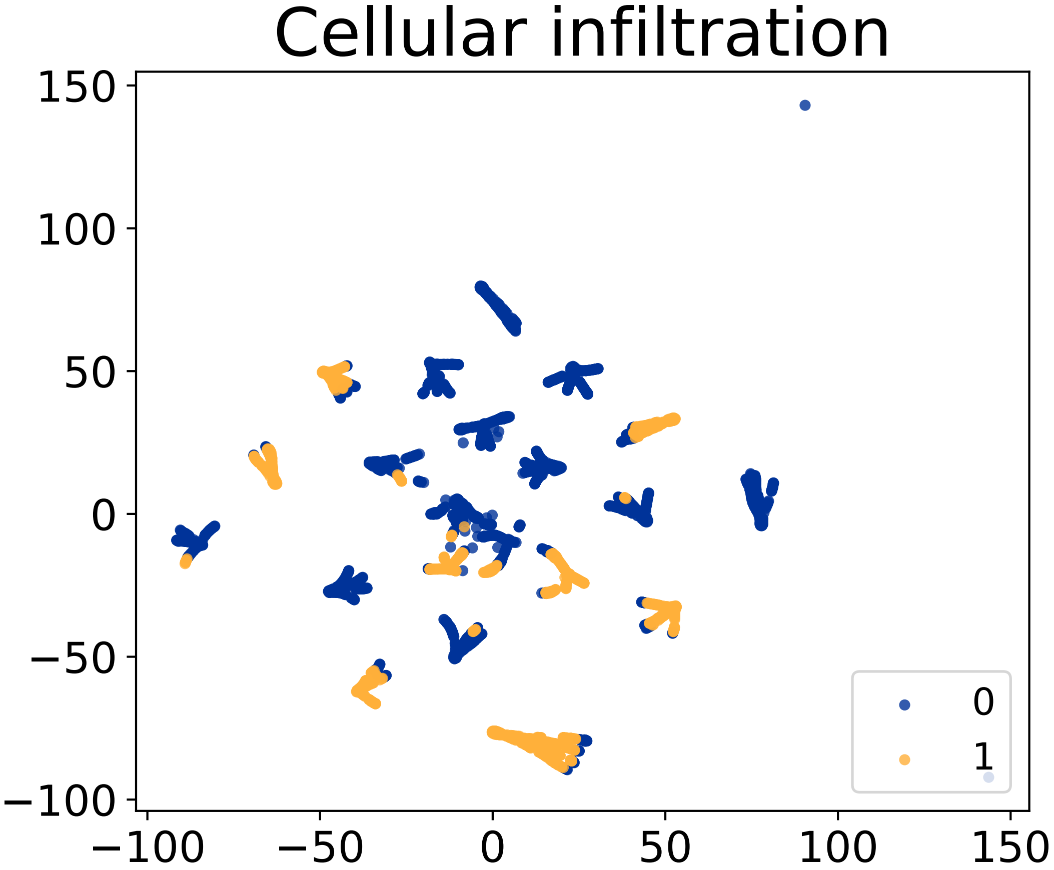

S1 Fig (b): The t-SNE visualization of cellular infiltration raw features on liver data.

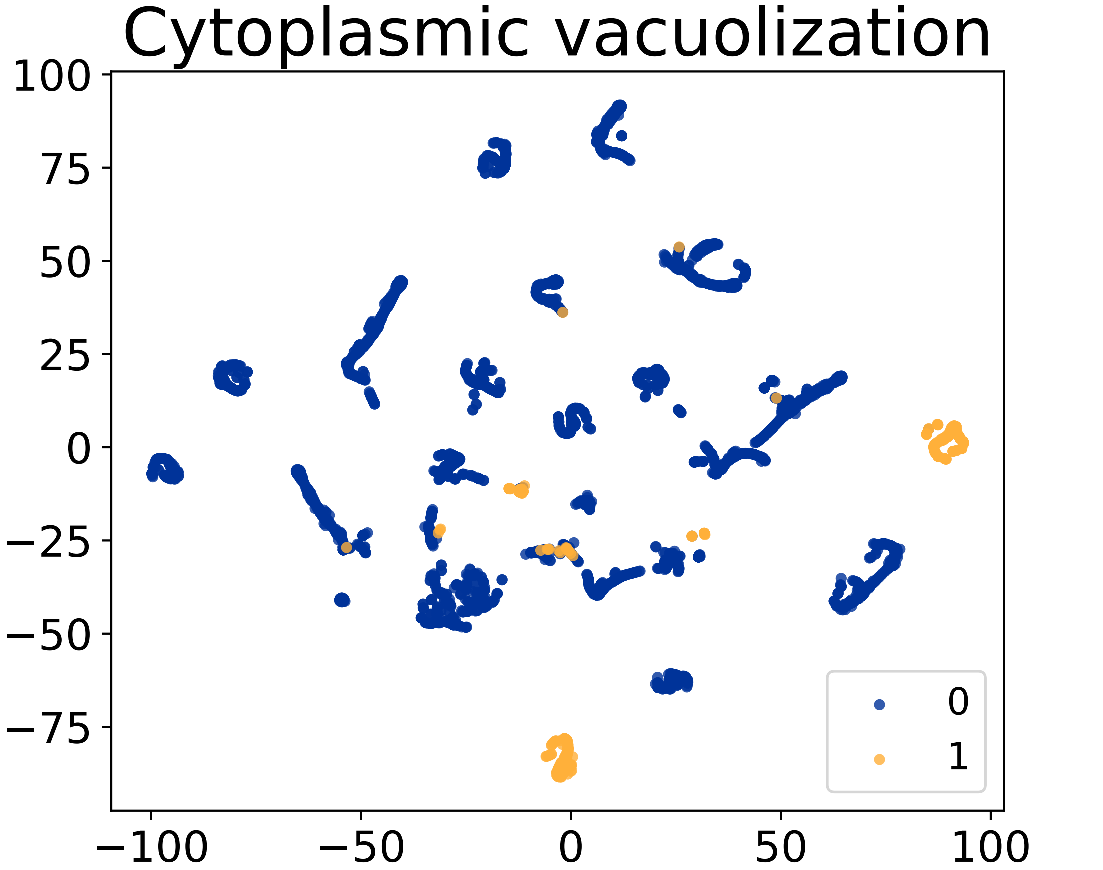

S1 Fig (c): The t-SNE visualization of cytoplasmic vacuolization features generated after RNN layer on liver data.

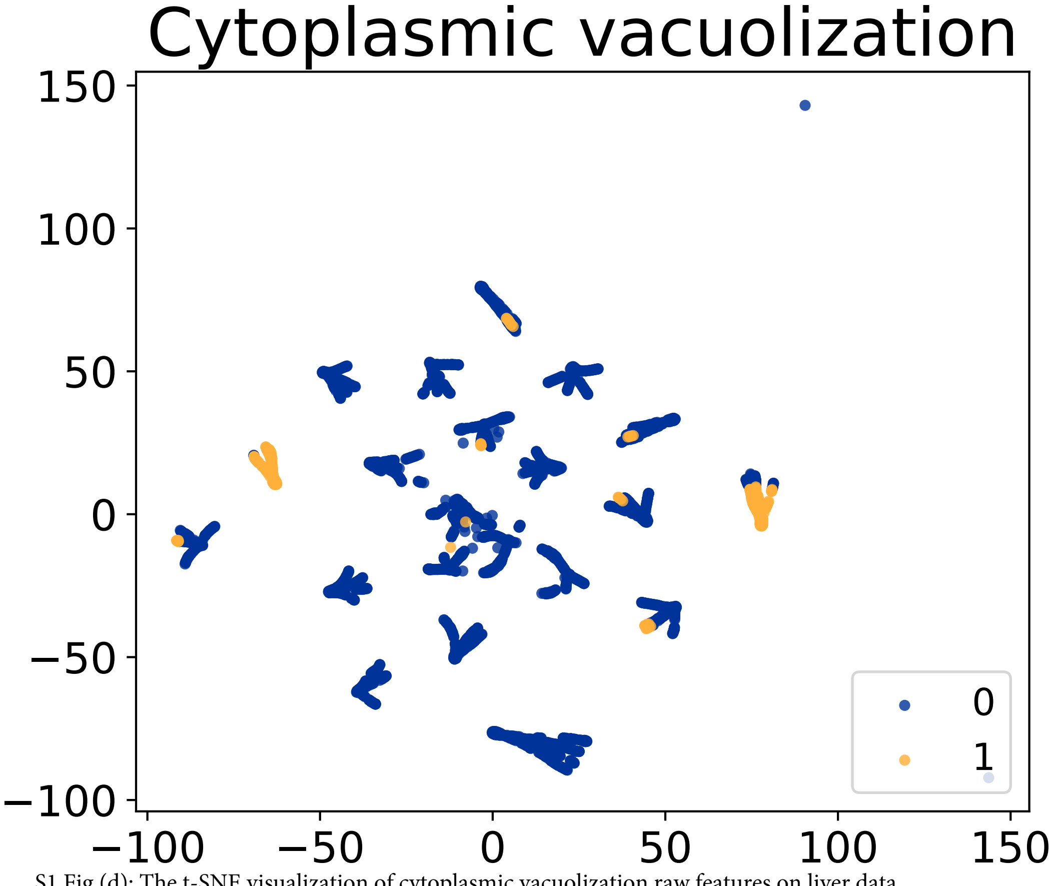

S1 Fig (d): The t-SNE visualization of cytoplasmic vacuolization raw features on liver data.

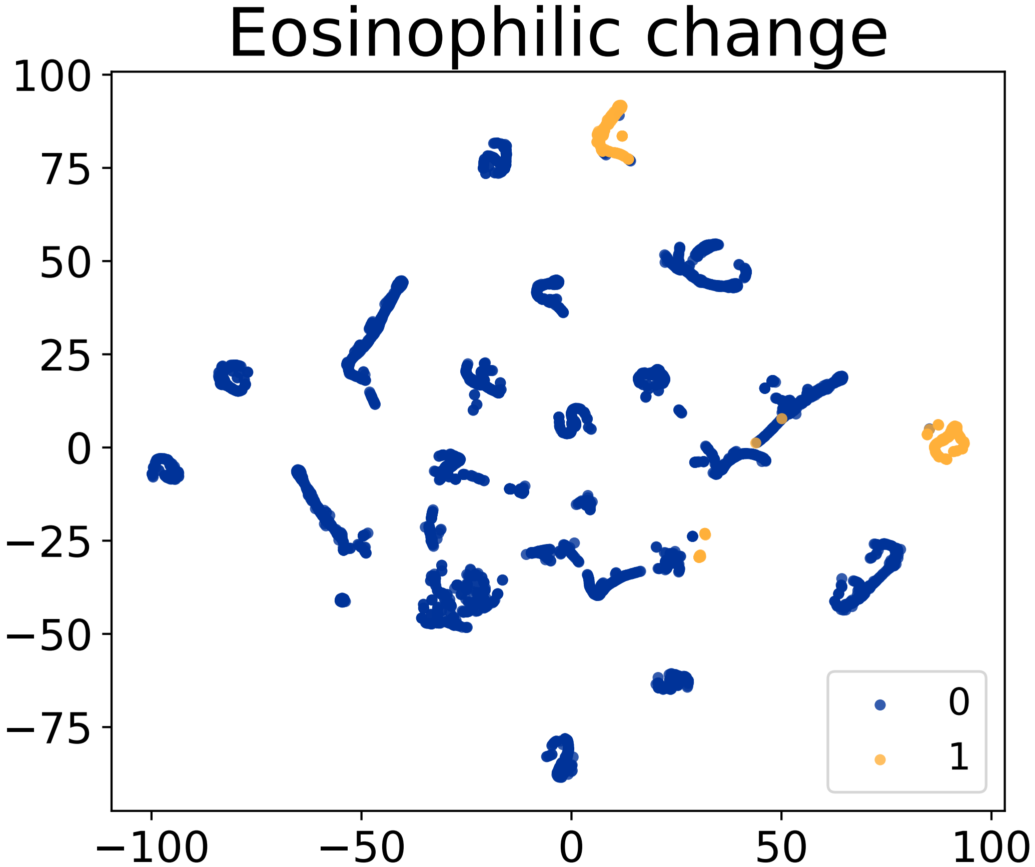

S1 Fig (e): The t-SNE visualization of eosinophilic change features generated after RNN layer on liver data.

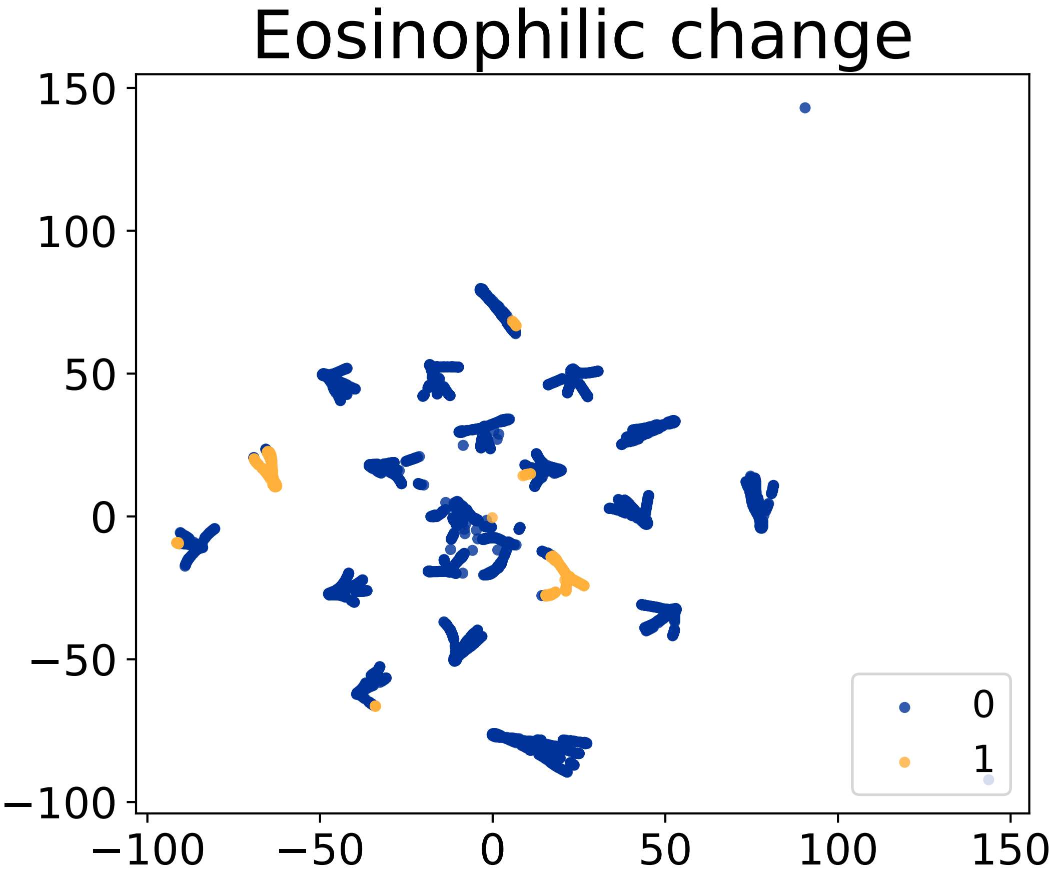


S1 Fig (f): The t-SNE visualization of eosinophilic change raw features on liver data.

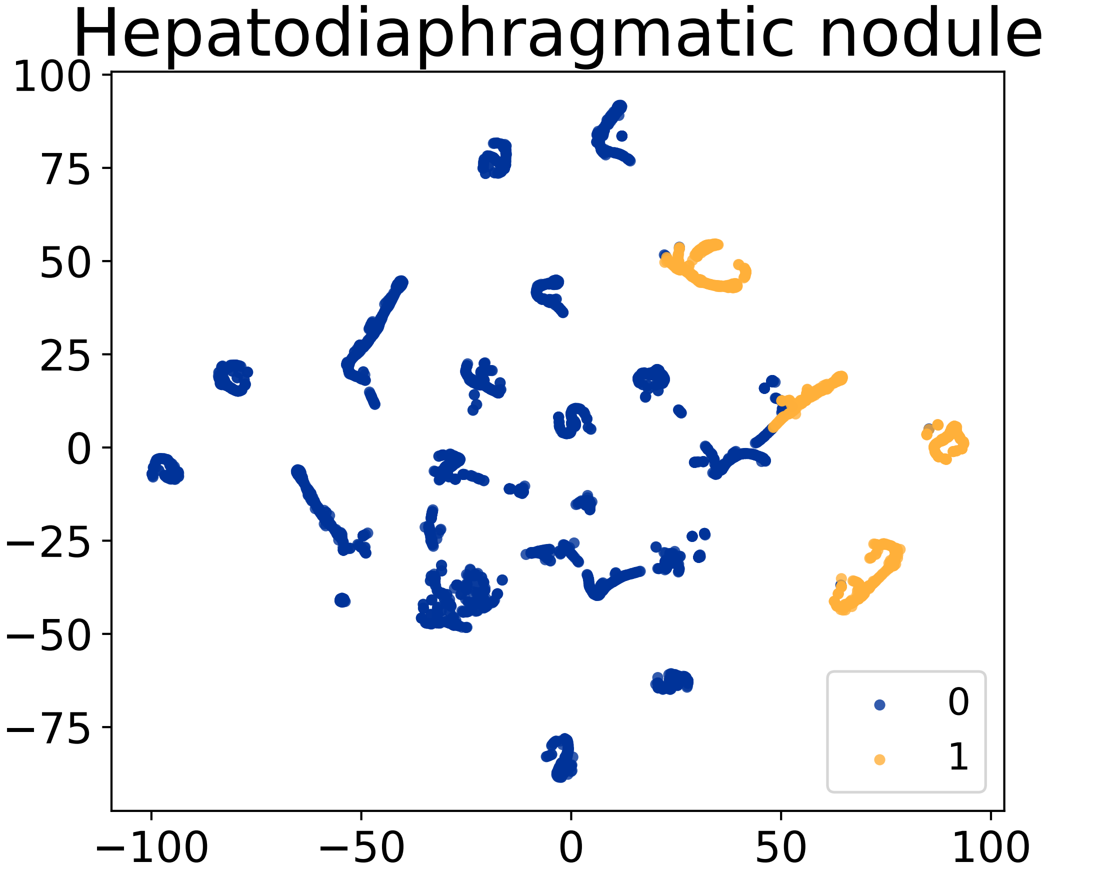


S1 Fig (g): The t-SNE visualization of hepatodiaphragmatic nodule features generated after RNN layer on liver data.

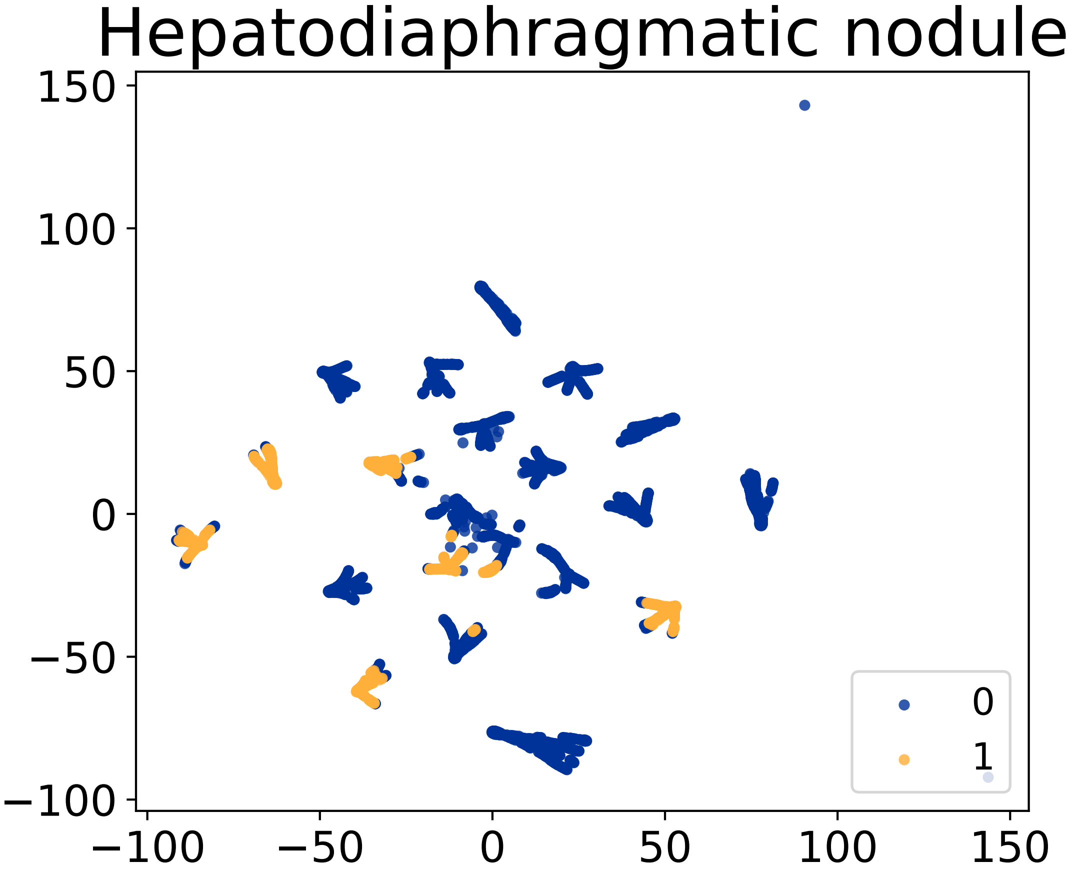

S1 Fig (h): The t-SNE visualization of hepatodiaphragmatic nodule raw features on liver data.

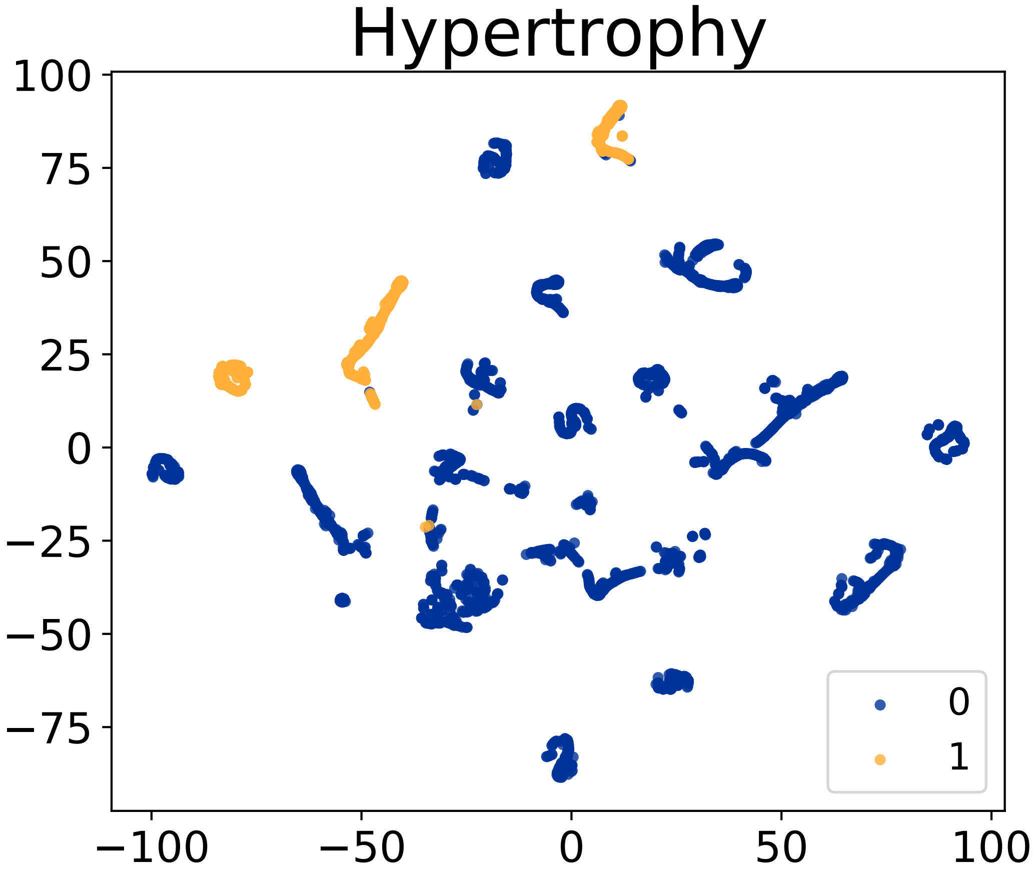

S1 Fig (i): The t-SNE visualization of hypertrophy features generated after RNN layer on liver data.

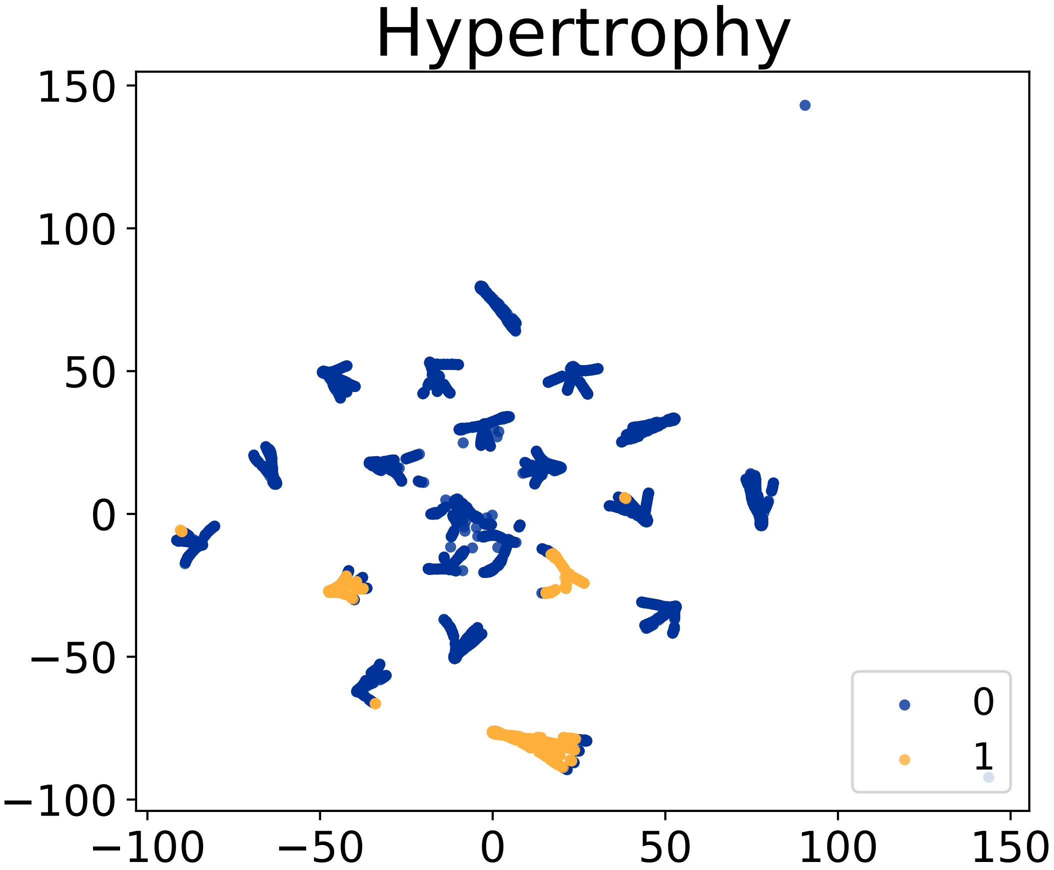

S1 Fig (j): The t-SNE visualization of hypertrophy raw features on liver data.

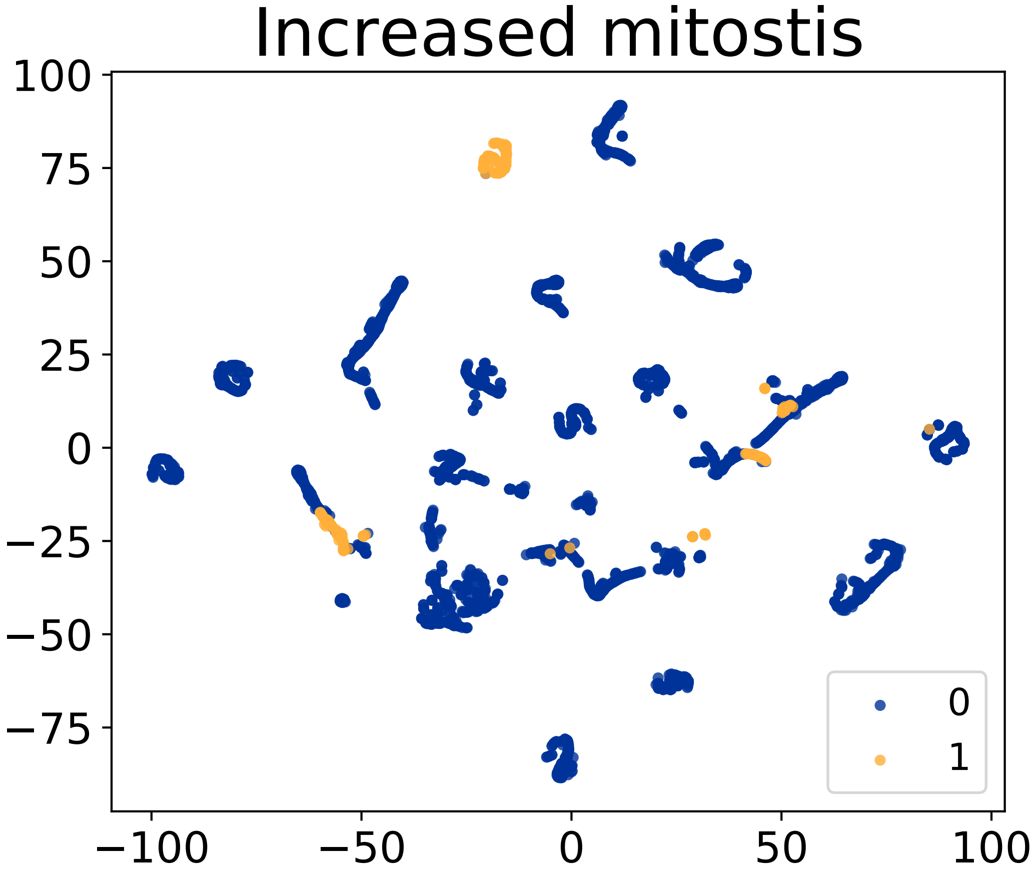

S1 Fig (k): The t-SNE visualization of increased mitostis features generated after RNN layer on liver data.

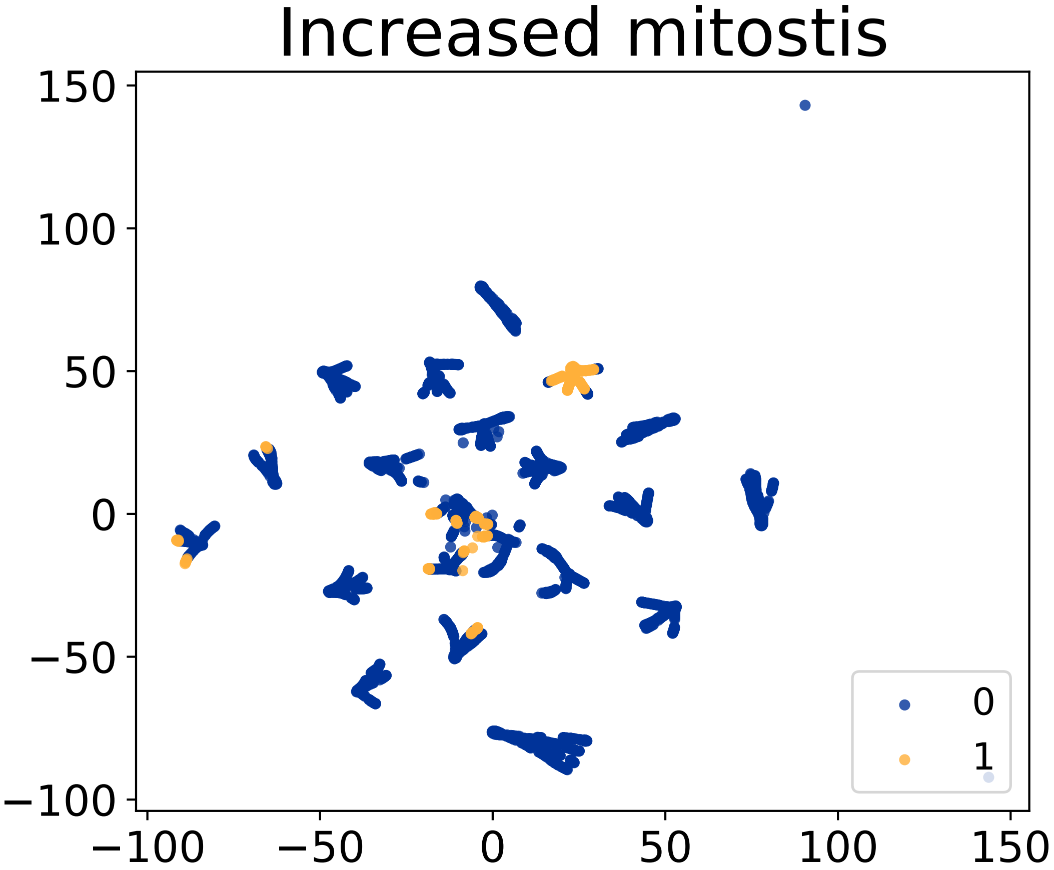

S1 Fig (l): The t-SNE visualization of increased mitostis raw features on liver data .

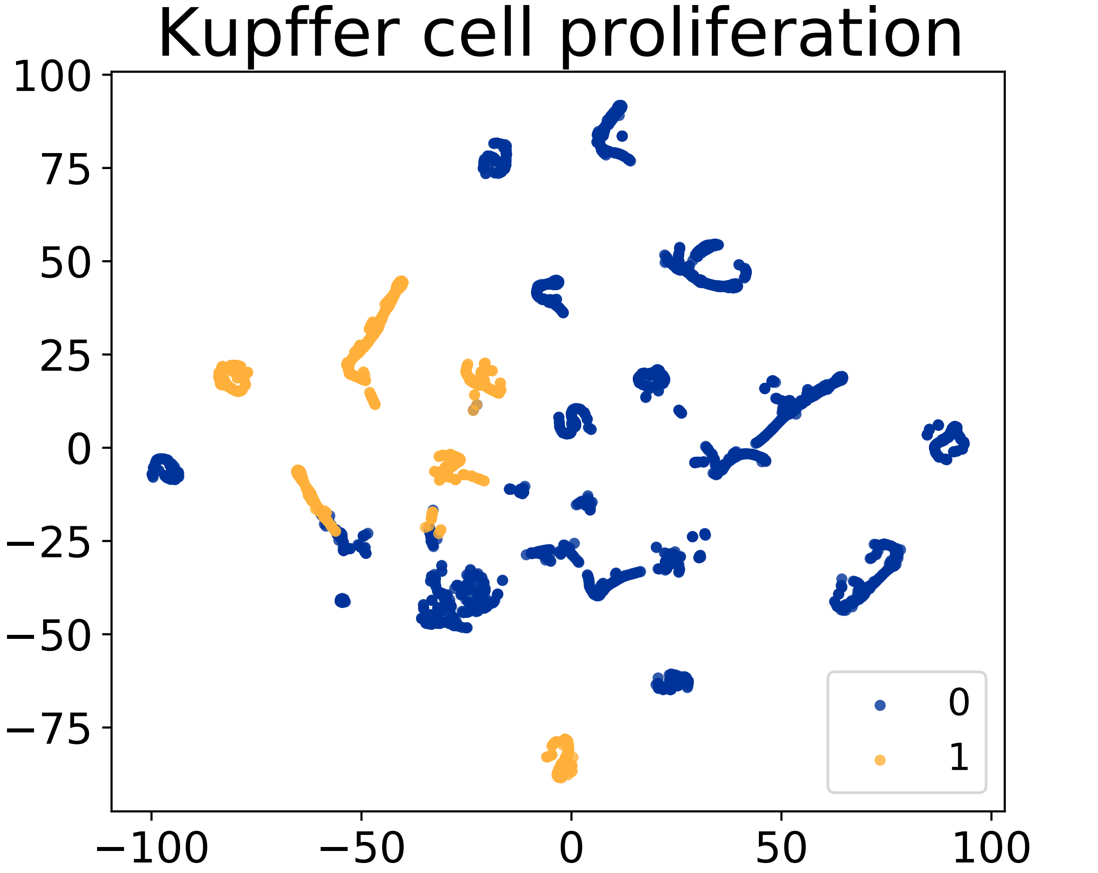

S1 Fig (m): The t-SNE visualization of kupffer cell proliferation features generated after RNN layer on liver data.

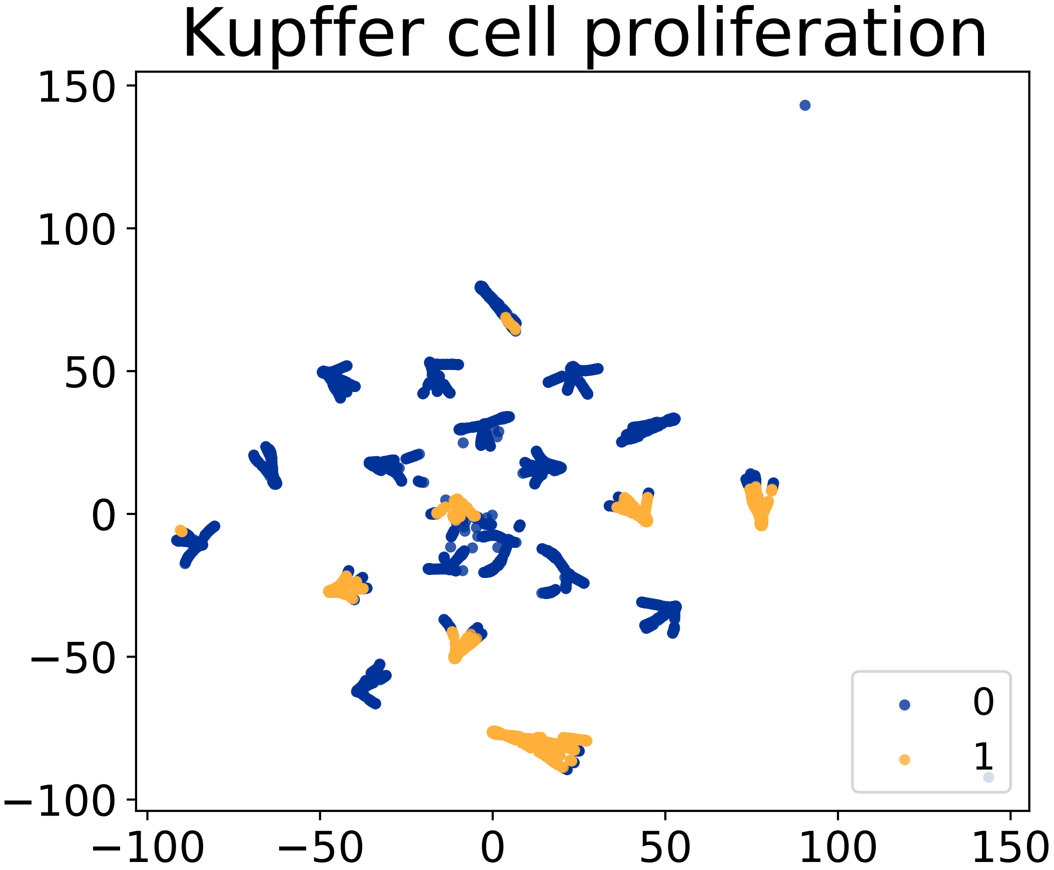

S1 Fig (n): The t-SNE visualization of kupffer cell proliferation raw features on liver data.

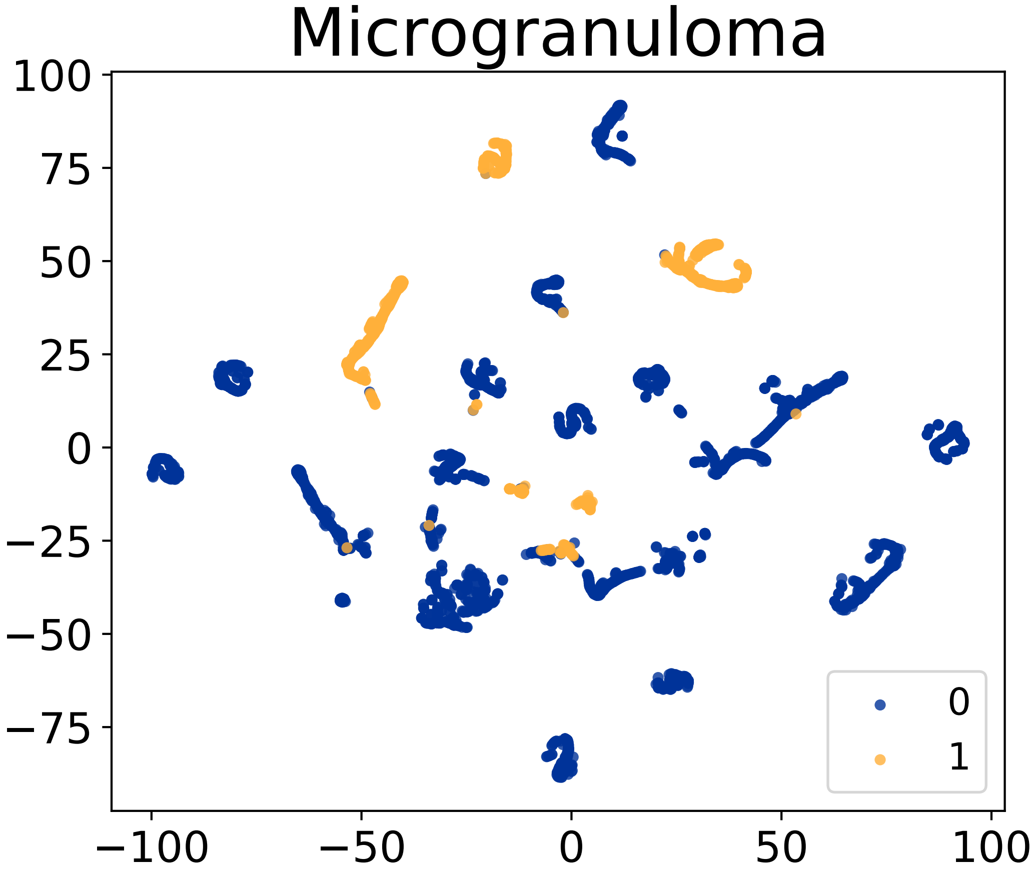


S1 Fig (o): The t-SNE visualization of microgranuloma features generated after RNN layer on liver data.

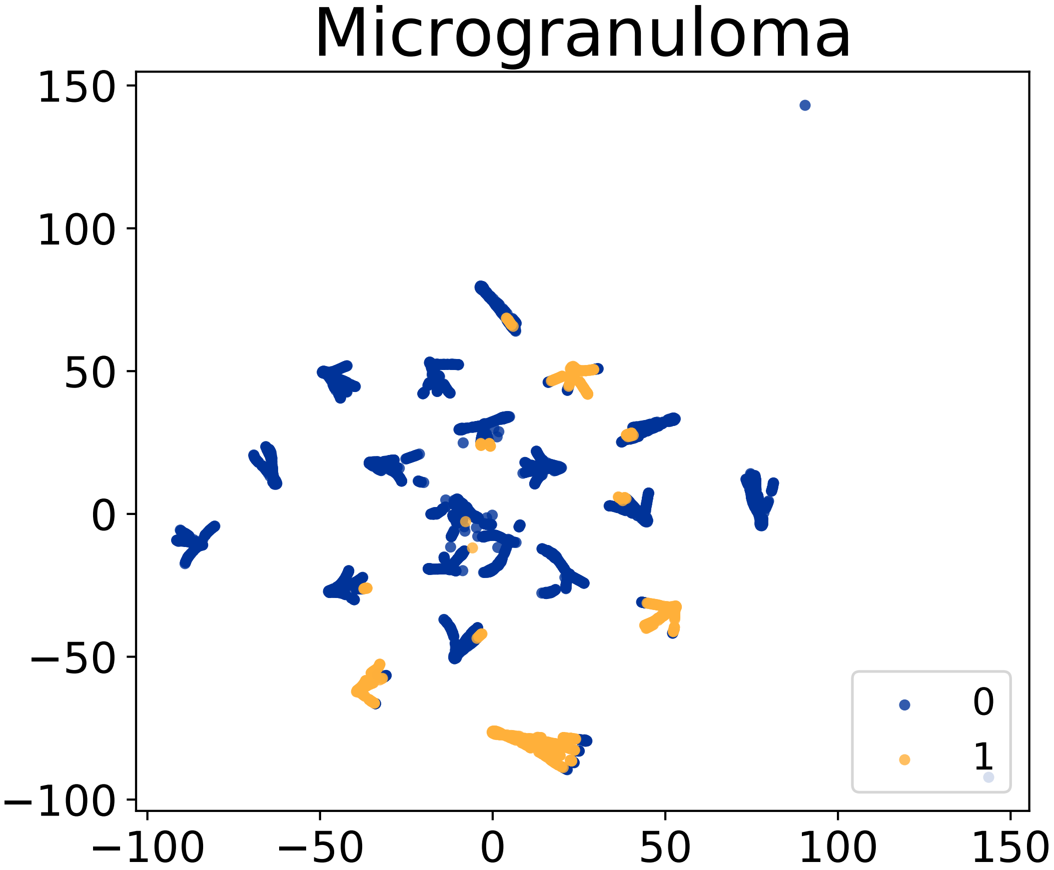

S1 Fig (p): The t-SNE visualization of microgranuloma raw features on liver data.

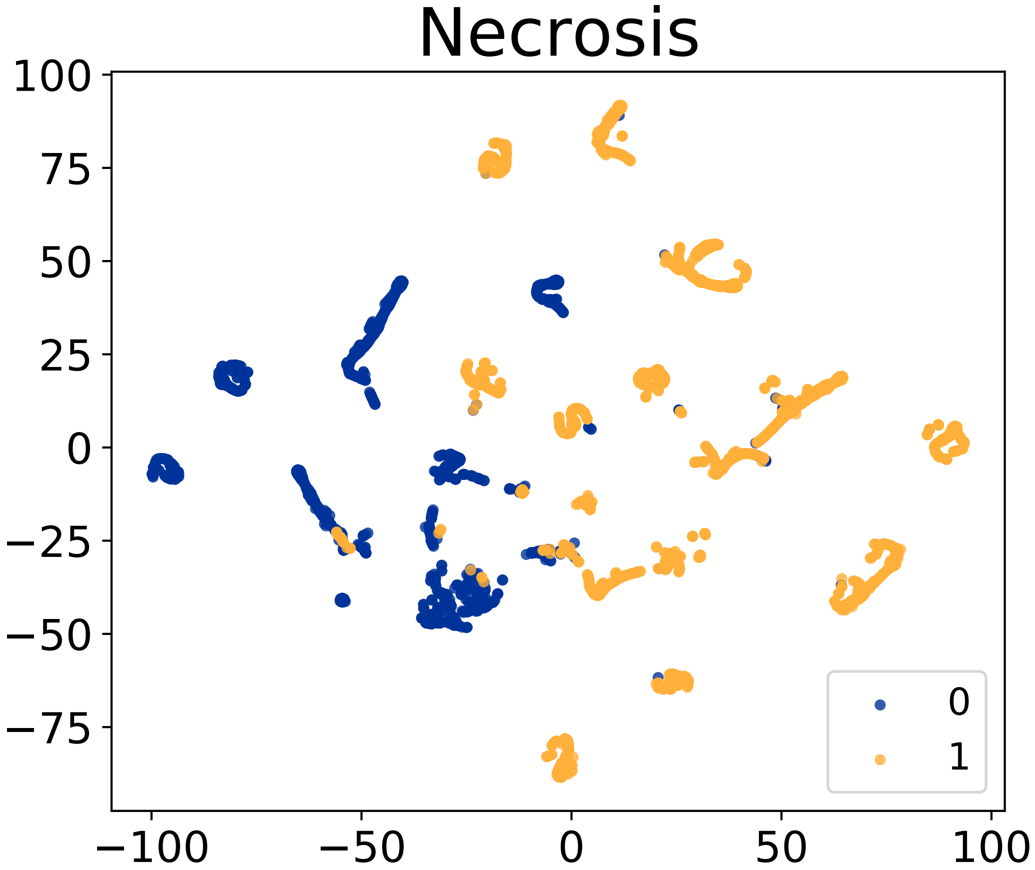

S1 Fig (q): The t-SNE visualization of necrosis features generated after RNN layer on liver data .

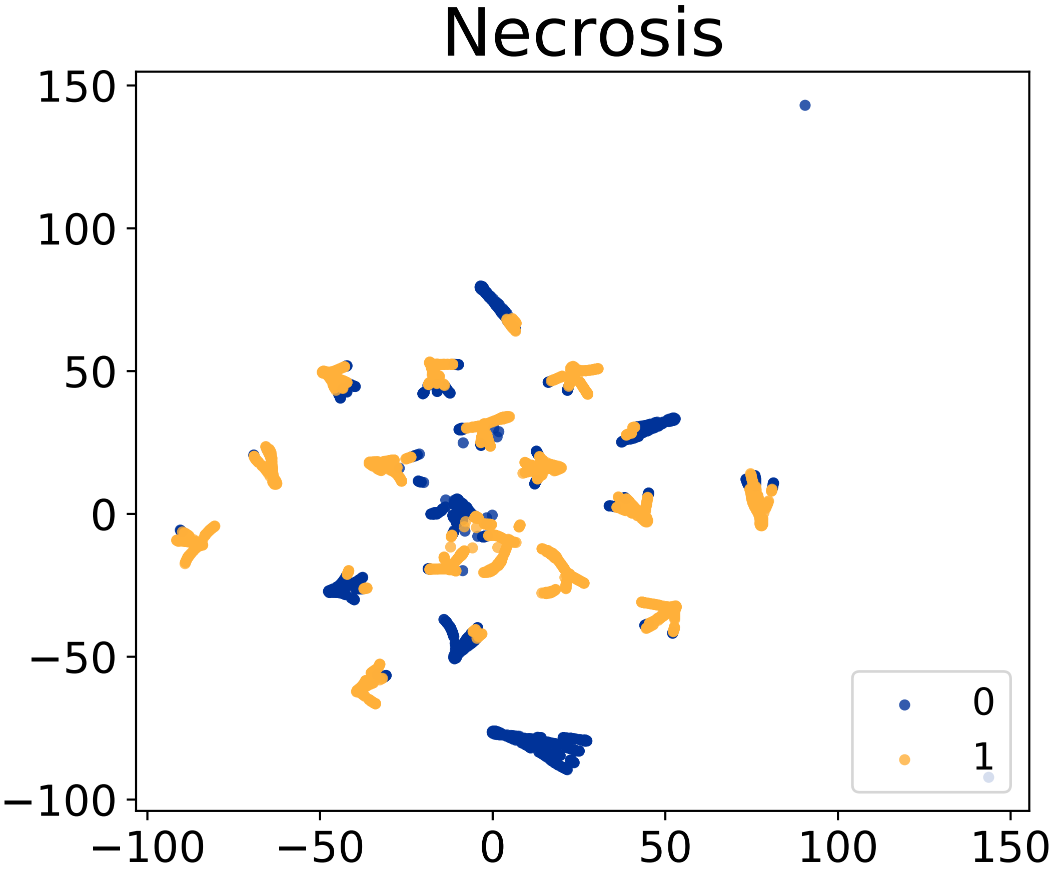

S1 Fig (r): The t-SNE visualization of necrosis raw features on liver data.

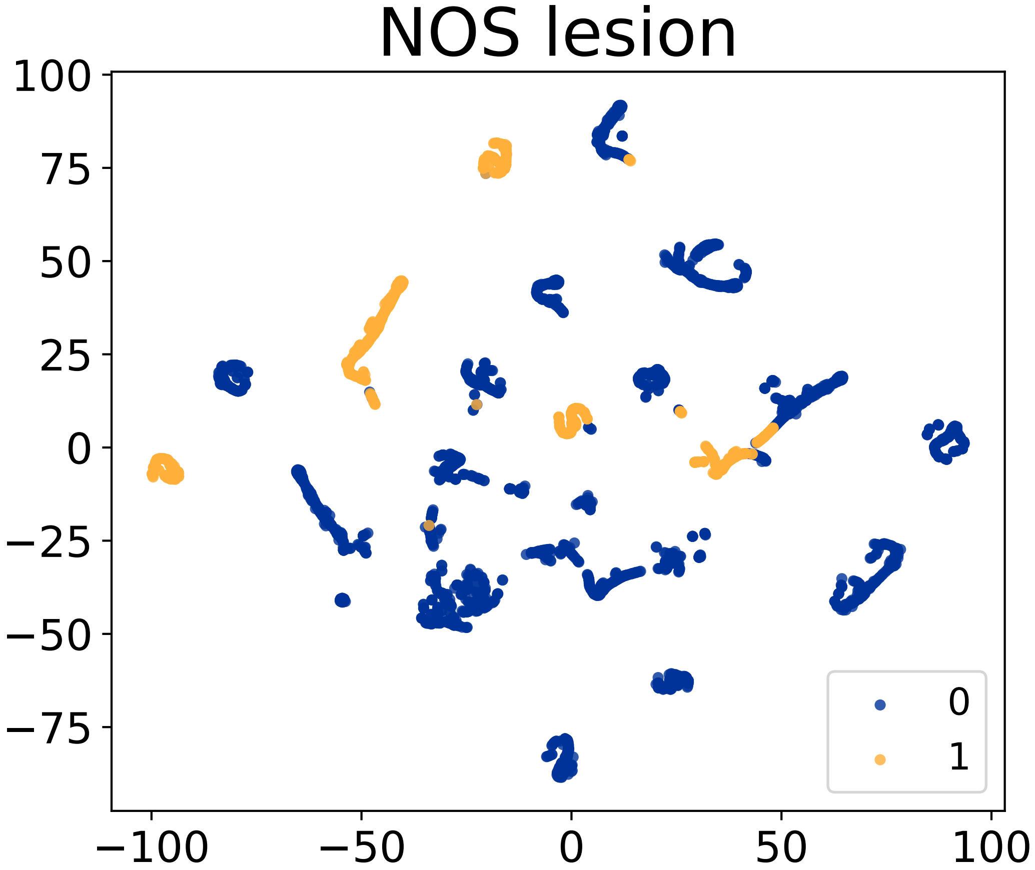

S1 Fig (s): The t-SNE visualization of NOS lesion features generated after RNN layer on liver data.

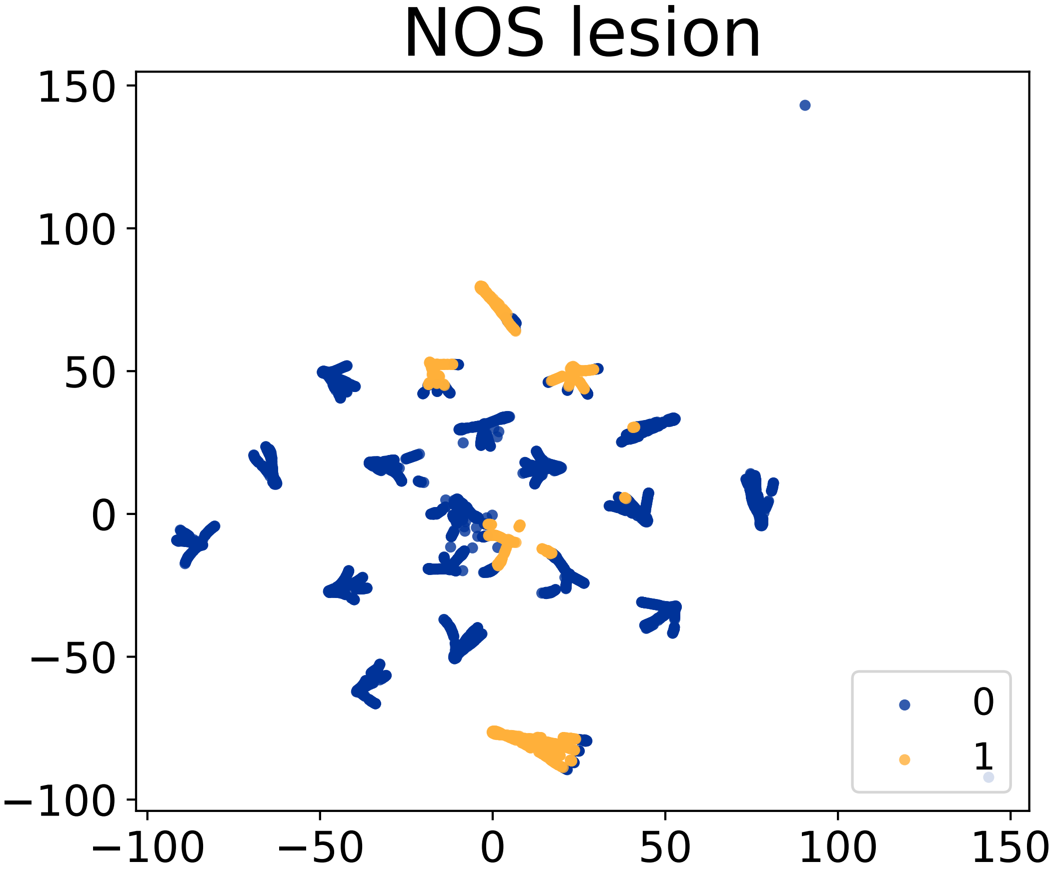

S1 Fig (t): The t-SNE visualization of NOS lesion raw features on liver data.

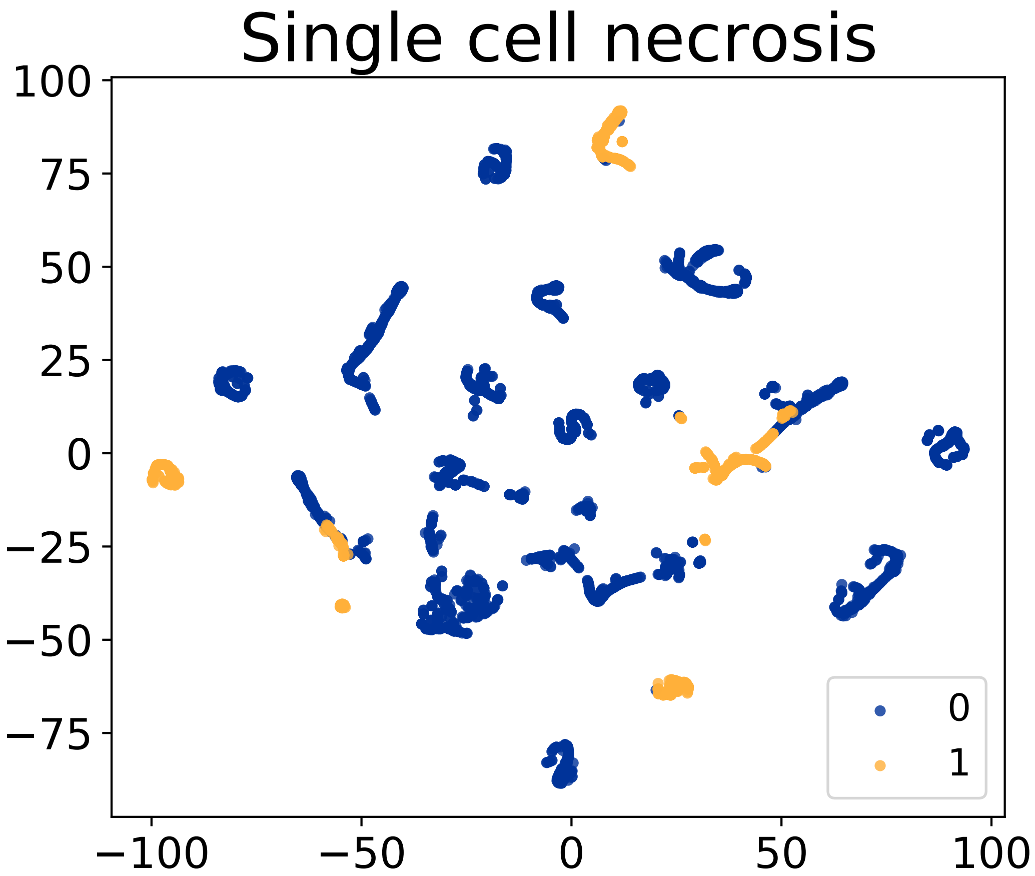

S1 Fig (u): The t-SNE visualization of single cell necrosis features generated after RNN layer on liver data.

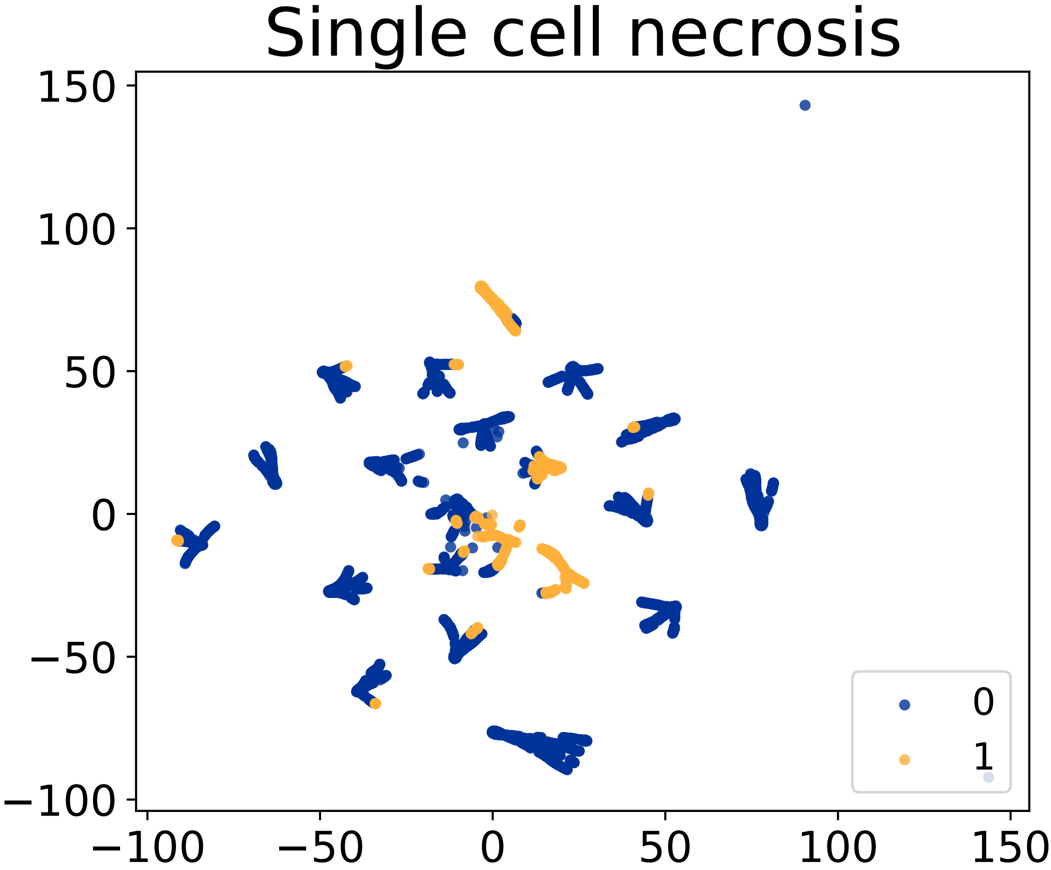

S1 Fig (v): The t-SNE visualization of single cell necrosis raw features on liver data.

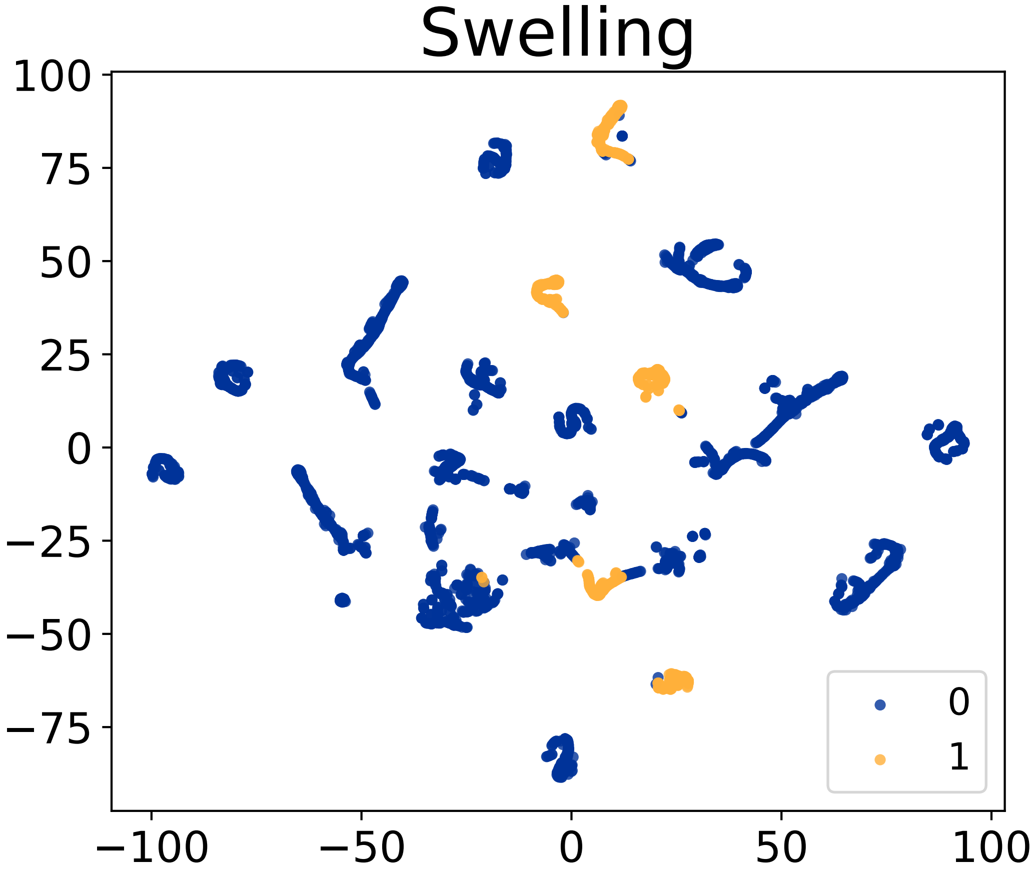

S1 Fig (w): The t-SNE visualization of swelling features generated after RNN layer on liver data .

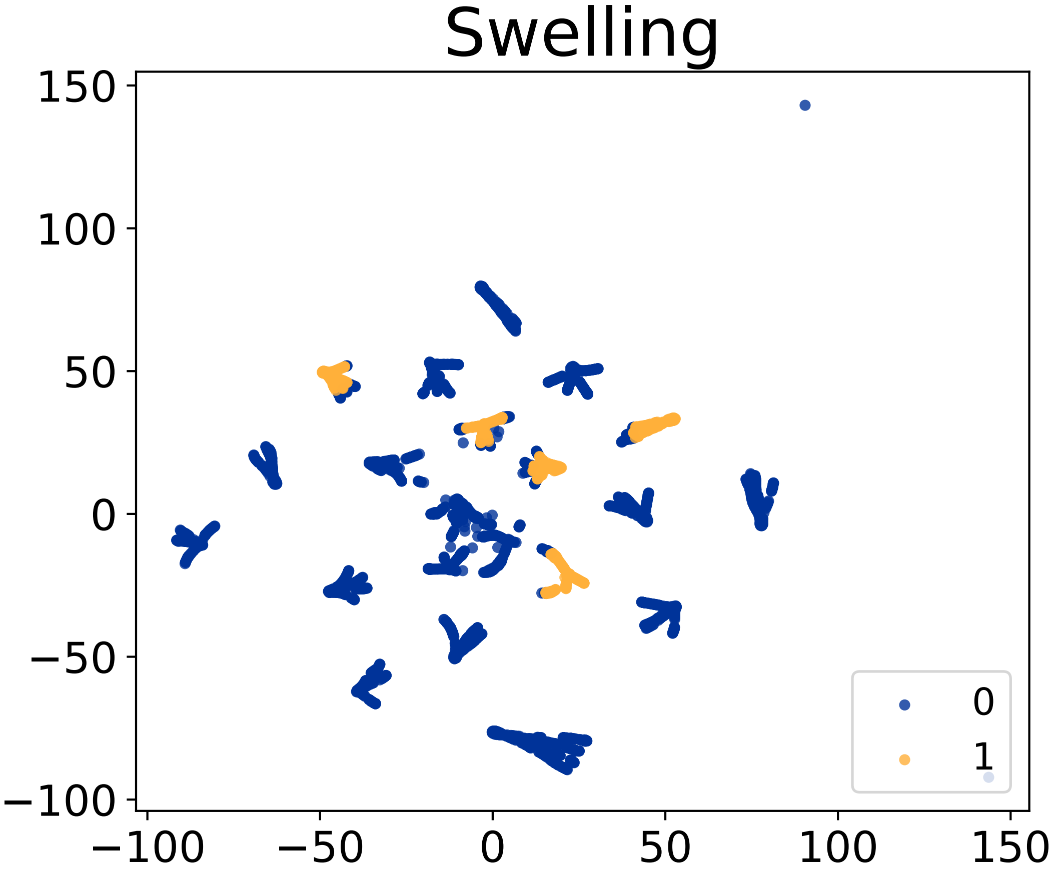

S1 Fig (x): The t-SNE visualization of swelling raw features on liver data.
