# Supplementary material for: A multi-label learning model for predicting drug-induced pathology in multi-organ based on toxicogenomics data
Source: PLoS Comput Biol. 2022 Sep 7;18(9):e1010402. doi: 10.1371/journal.pcbi.1010402 (PMC9451100; doi:10.1371/journal.pcbi.1010402)
Supplement: S2 Fig — (PDF) [file pcbi.1010402.s006.pdf]

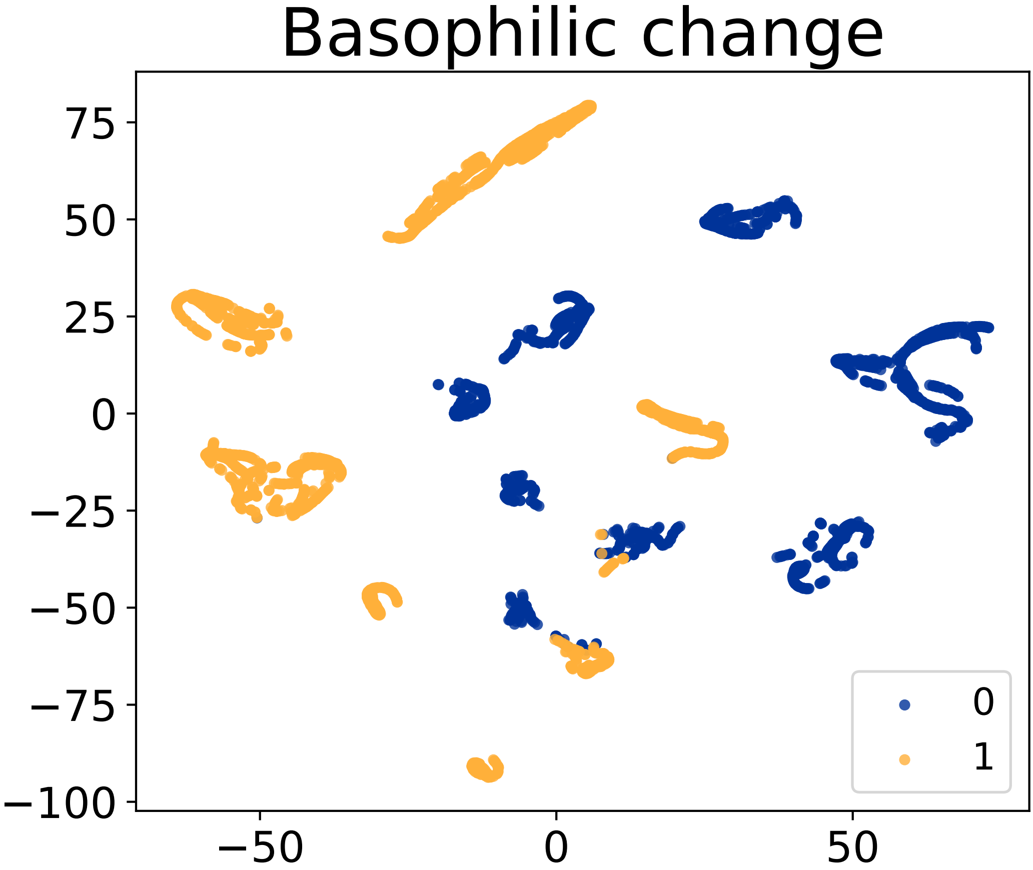

S2 Fig (a): The t-SNE visualization of basophilic change features generated after RNN layer on kidney data.

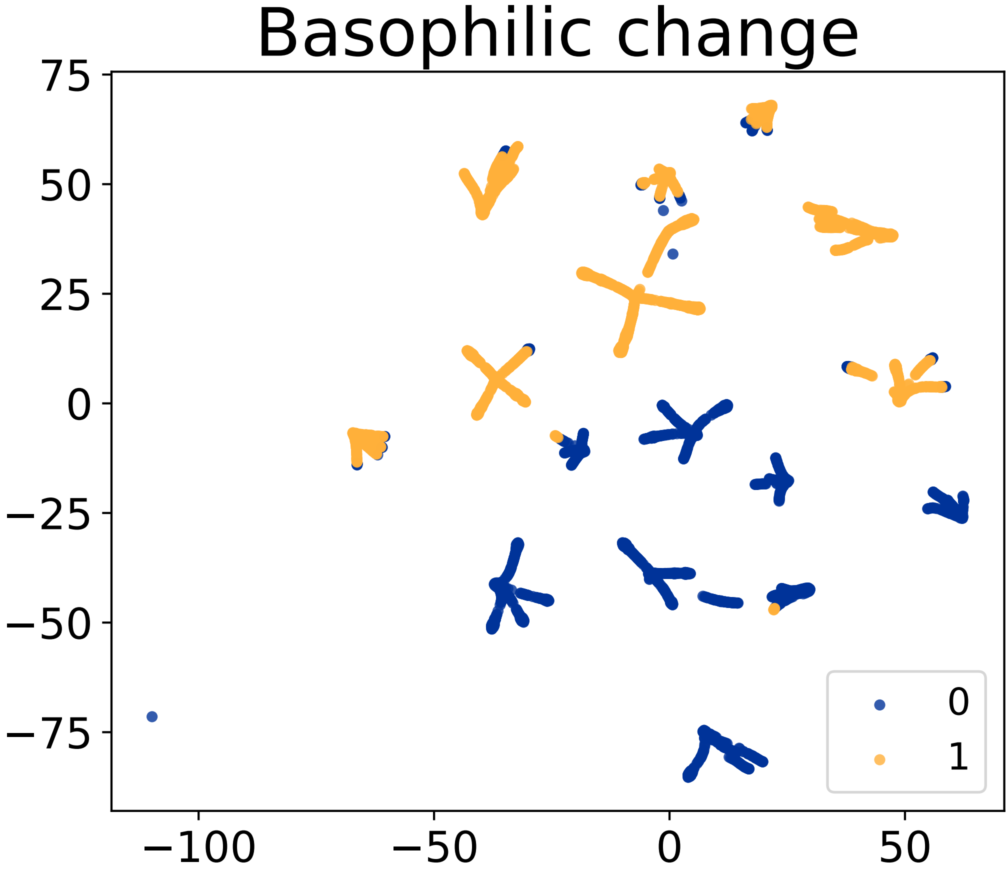

S2 Fig (b): The t-SNE visualization of basophilic change raw features on kidney data.

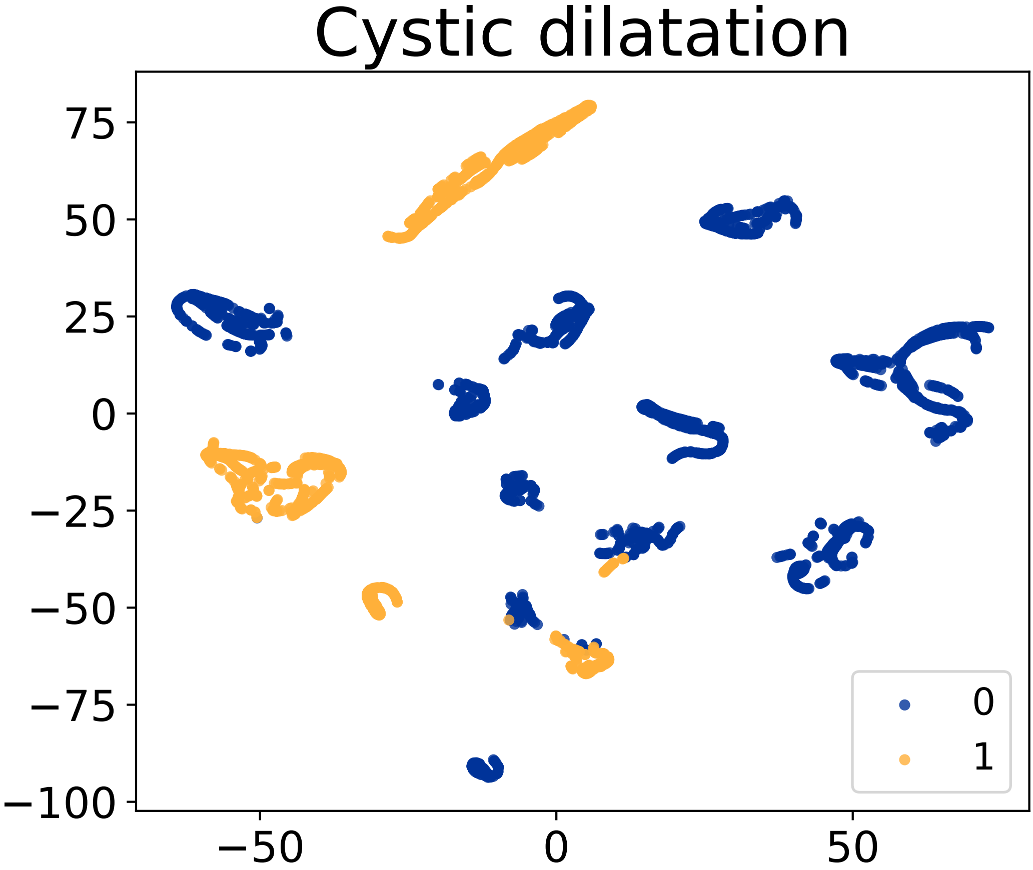

S2 Fig (c): The t-SNE visualization of cystic dilatation features generated after RNN layer on kidney data.

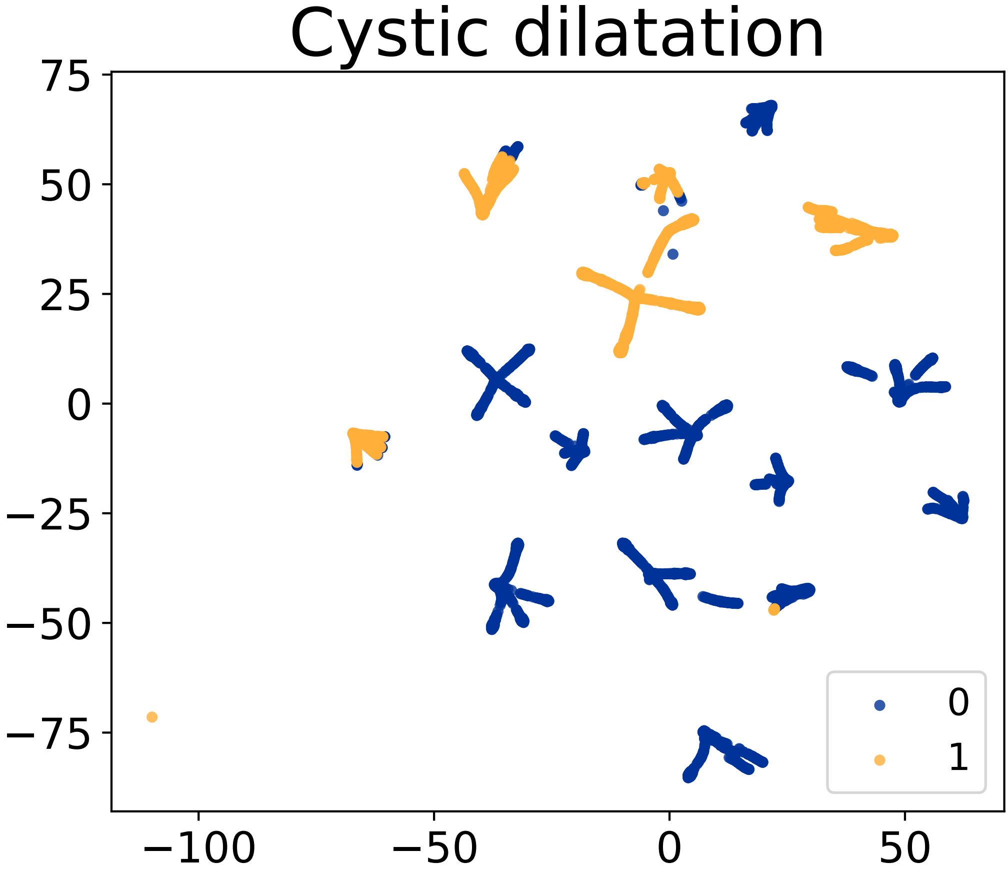

S2 Fig (d): The t-SNE visualization of cystic dilatation raw features on kidney data.

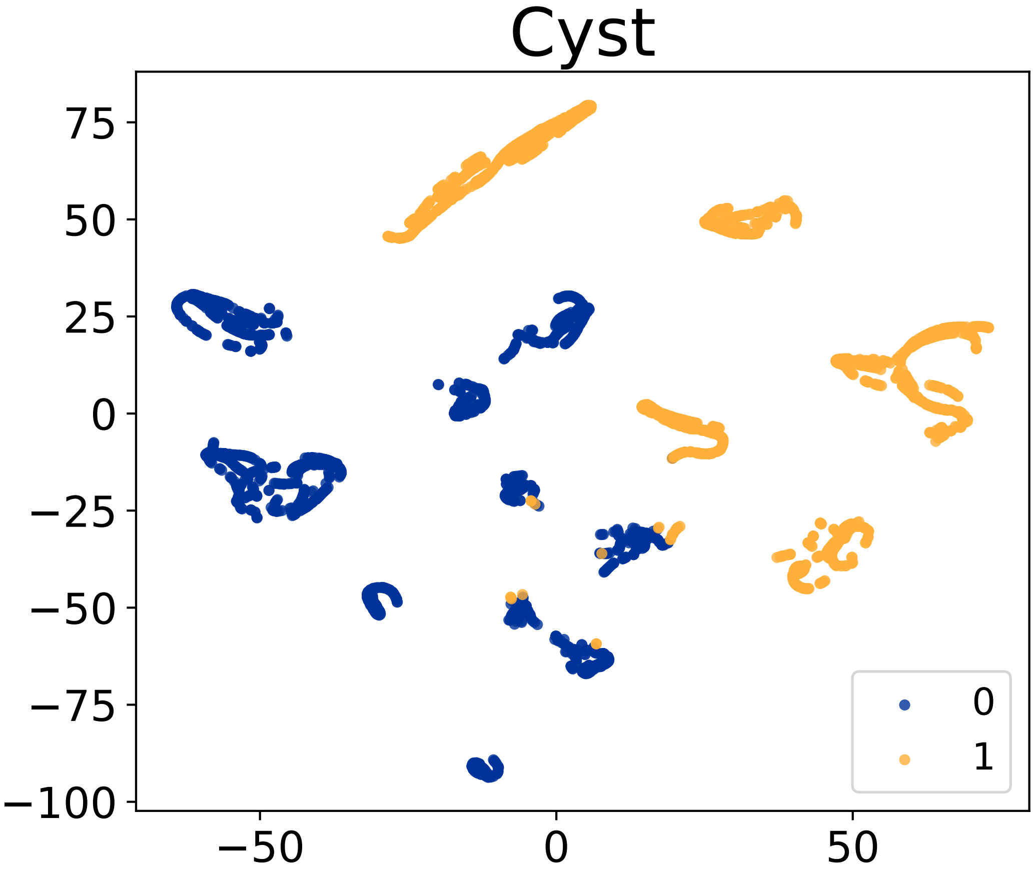

S2 Fig (e): The t-SNE visualization of cyst features generated after RNN layer on kidney data.

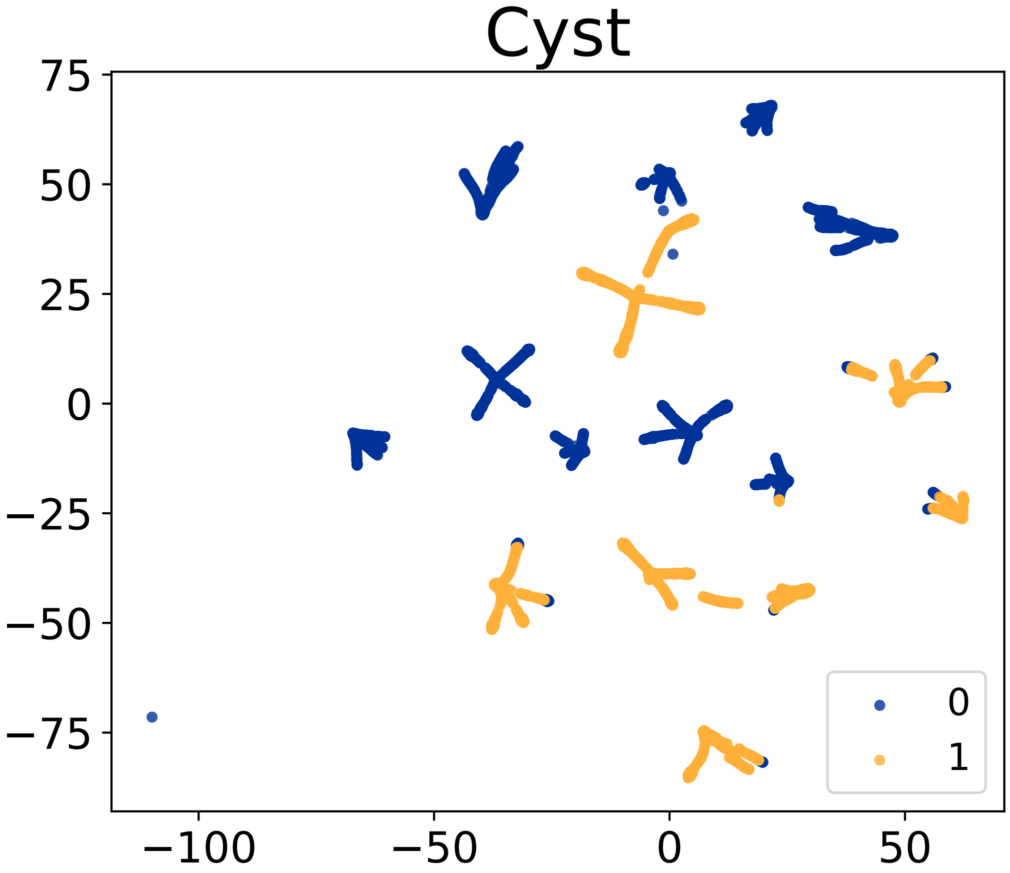

S2 Fig (f): The t-SNE visualization of cyst raw features on kidney data .

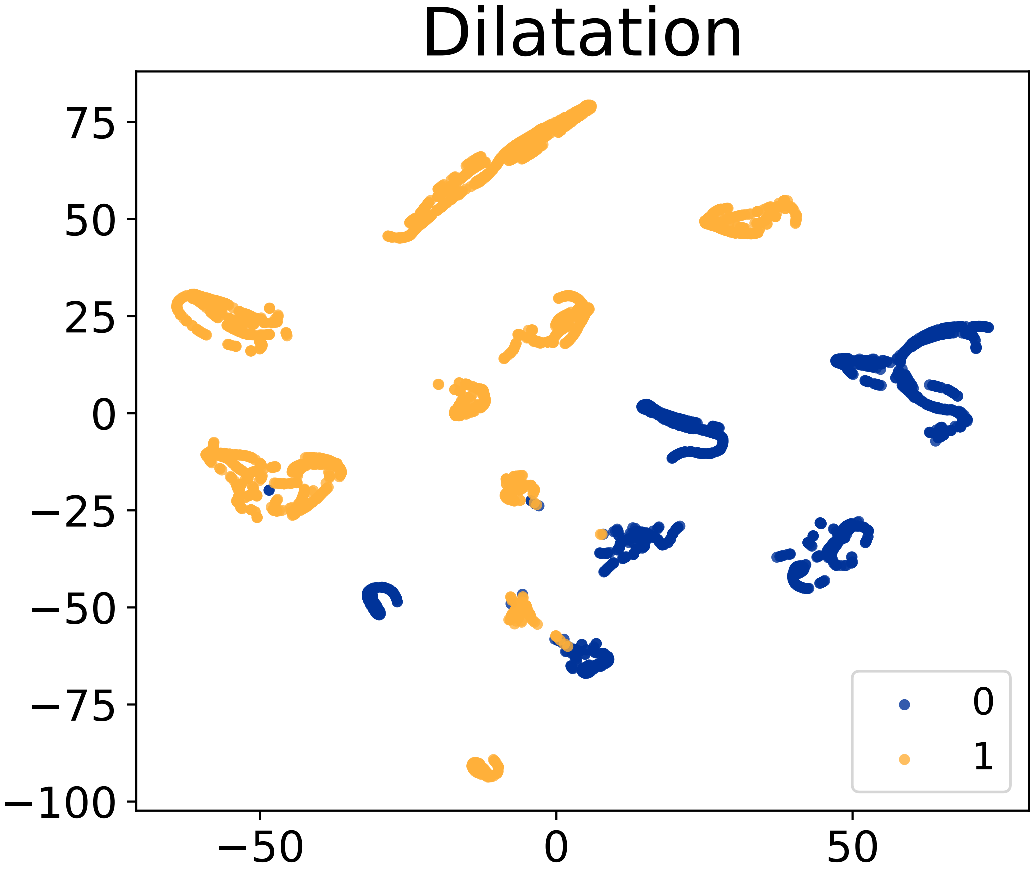

S2 Fig (g): The t-SNE visualization of dilatation features generated after RNN layer on kidney data.

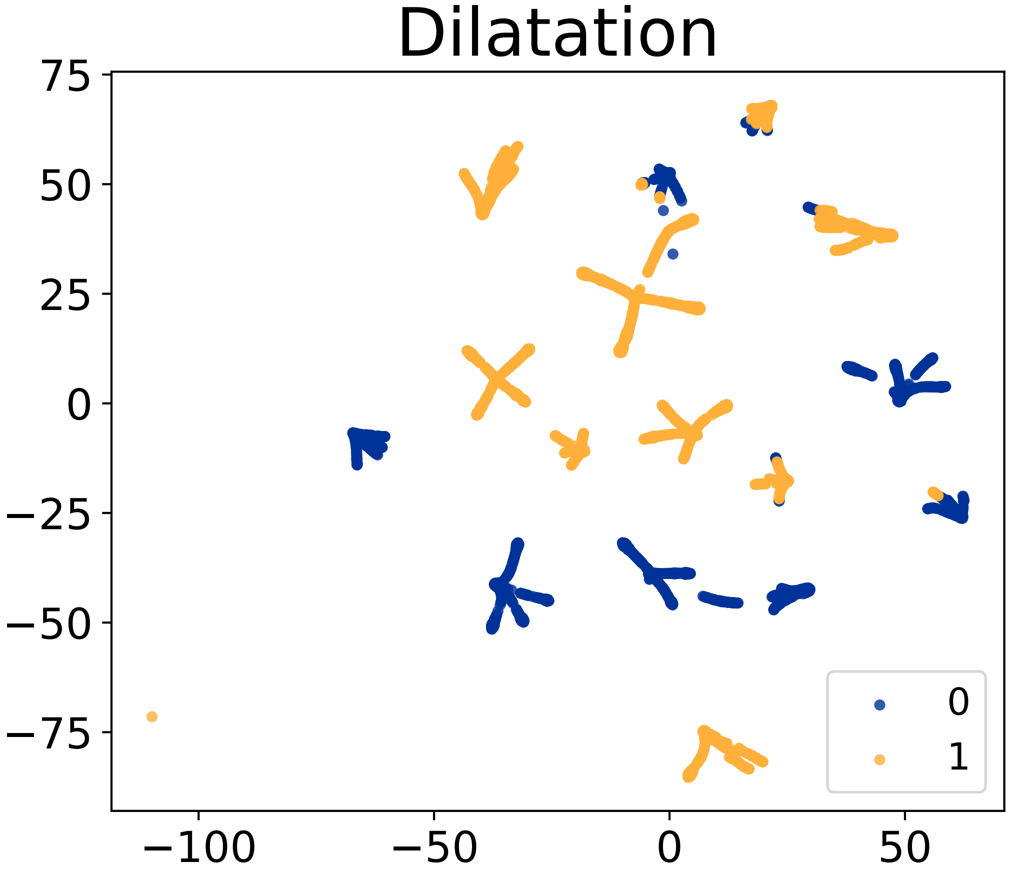

S2 Fig (h): The t-SNE visualization of dilatation raw features on kidney data.

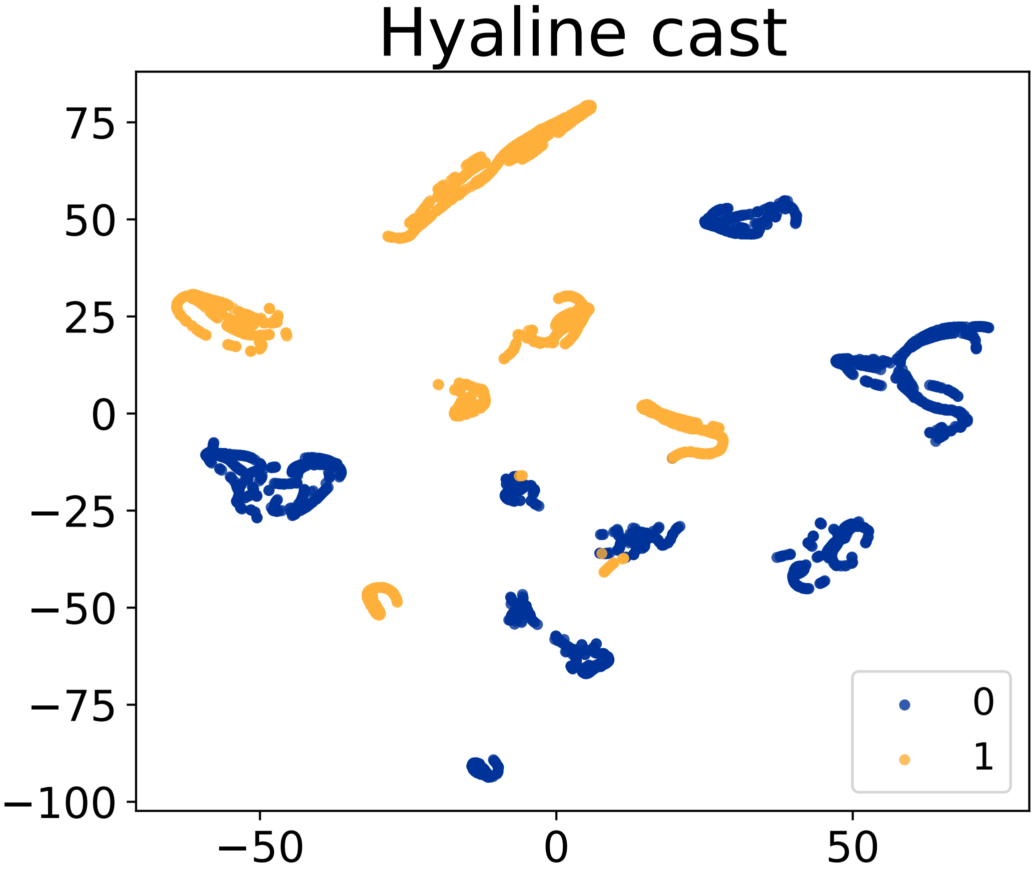

S2 Fig (i): The t-SNE visualization of hyaline cast features generated after RNN layer on kidney data.

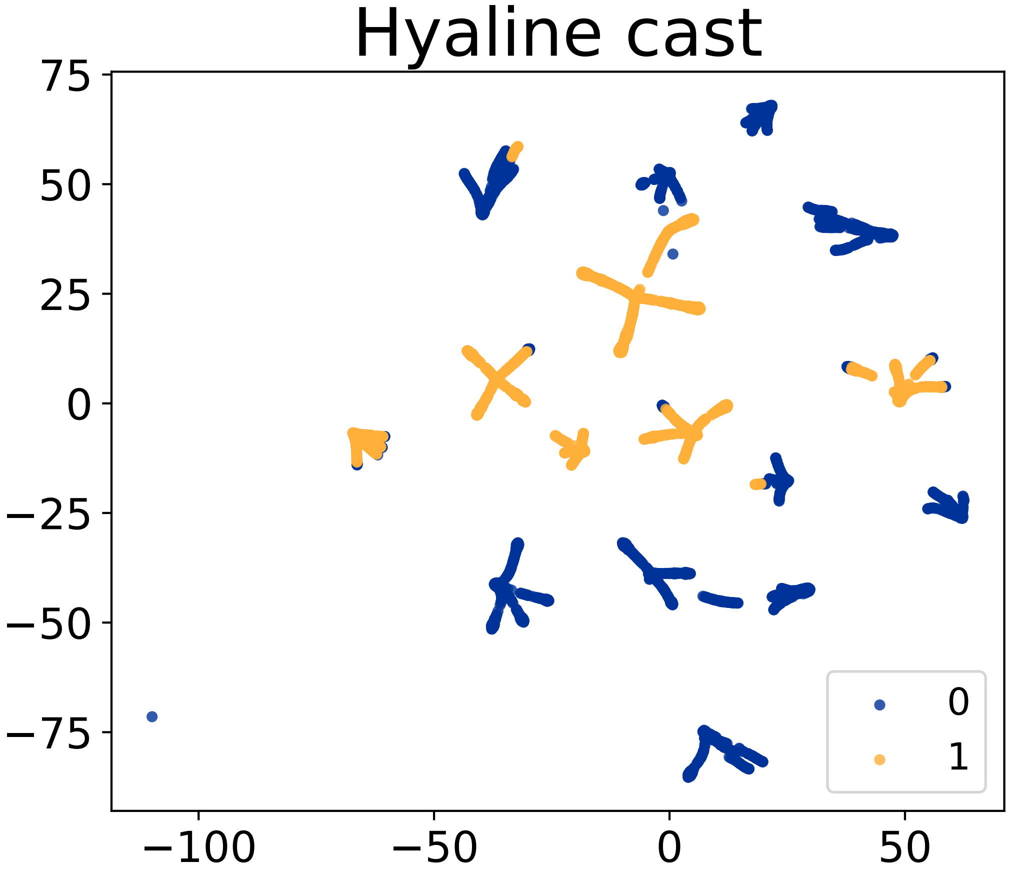

S2 Fig (j): The t-SNE visualization of hyaline cast raw features on kidney data.

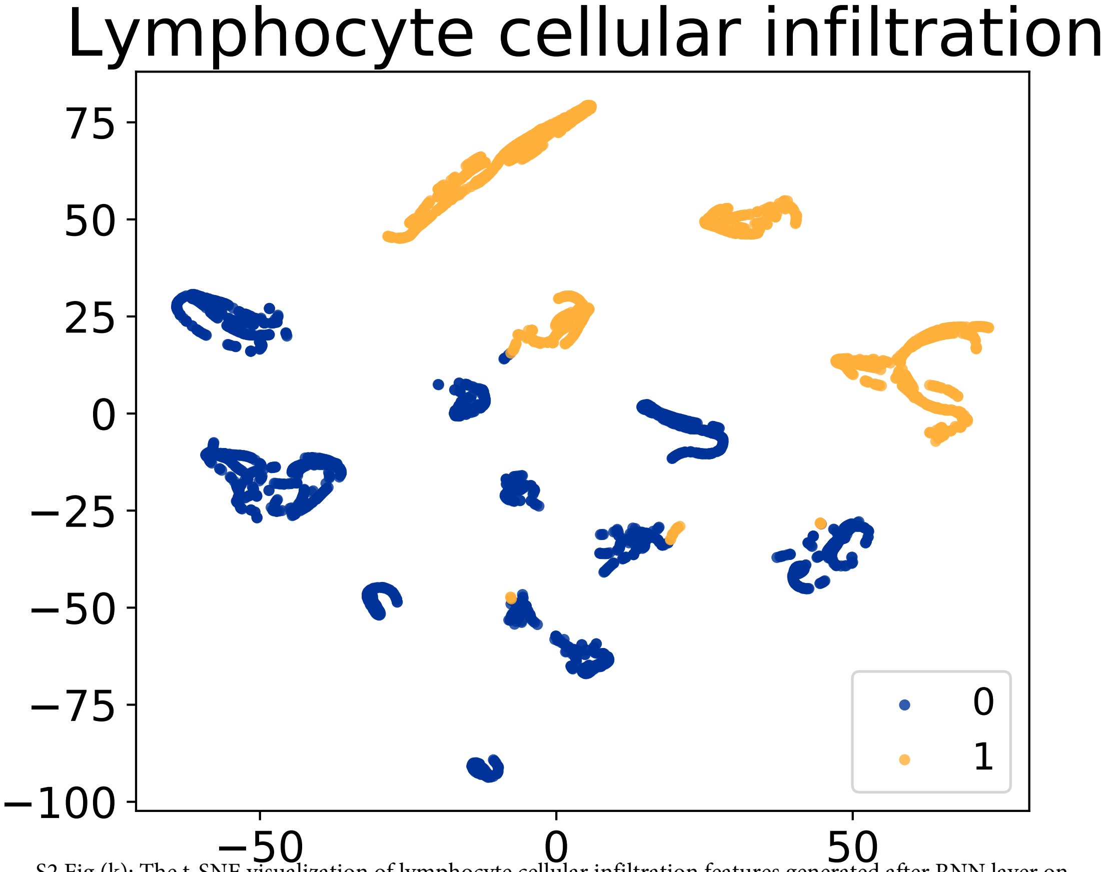

S2 Fig (k): The t-SNE visualization of lymphocyte cellular infiltration features generated after RNN layer on kidney data.

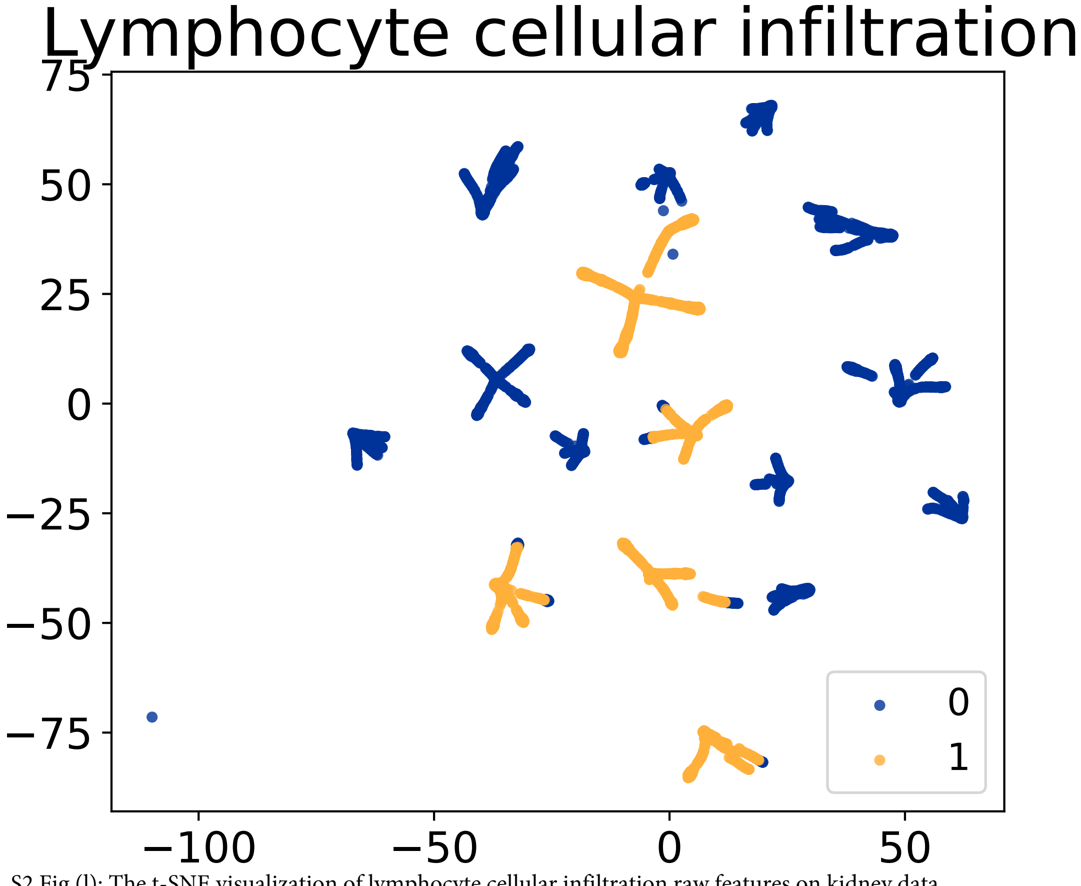

S2 Fig (l): The t-SNE visualization of lymphocyte cellular infiltration raw features on kidney data.

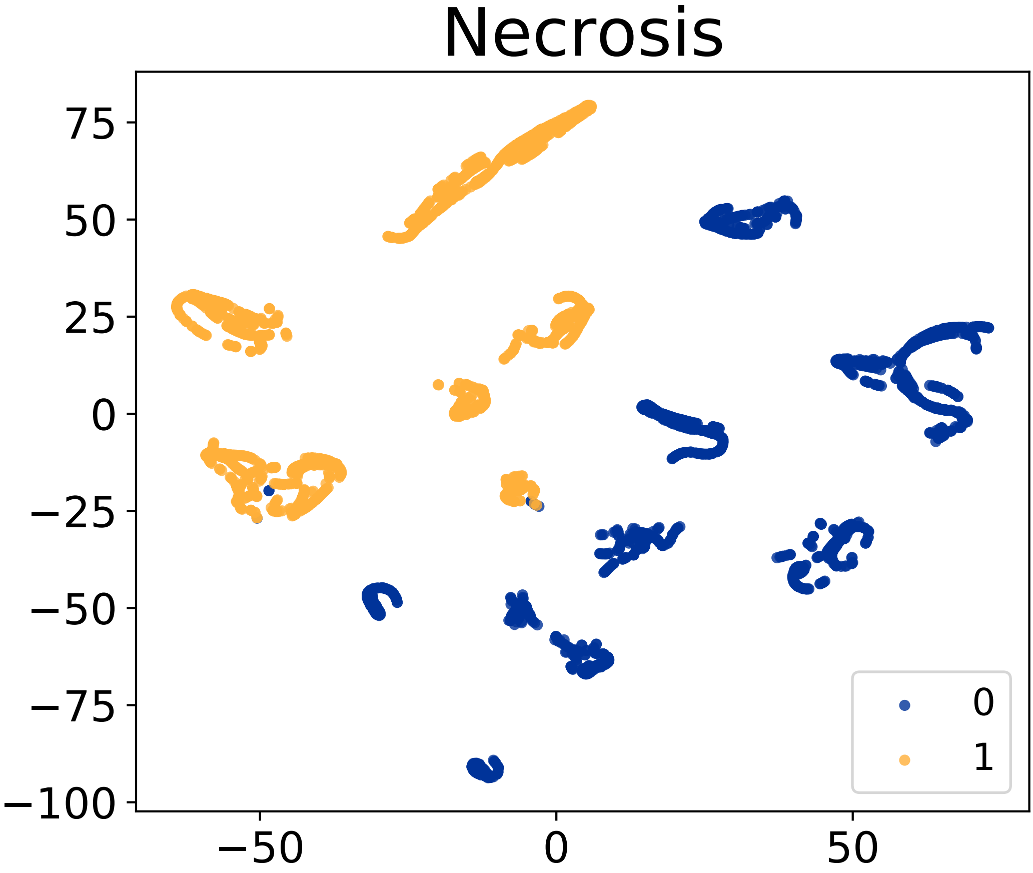

S2 Fig (m): The t-SNE visualization of necrosis features generated after RNN layer on kidney data.

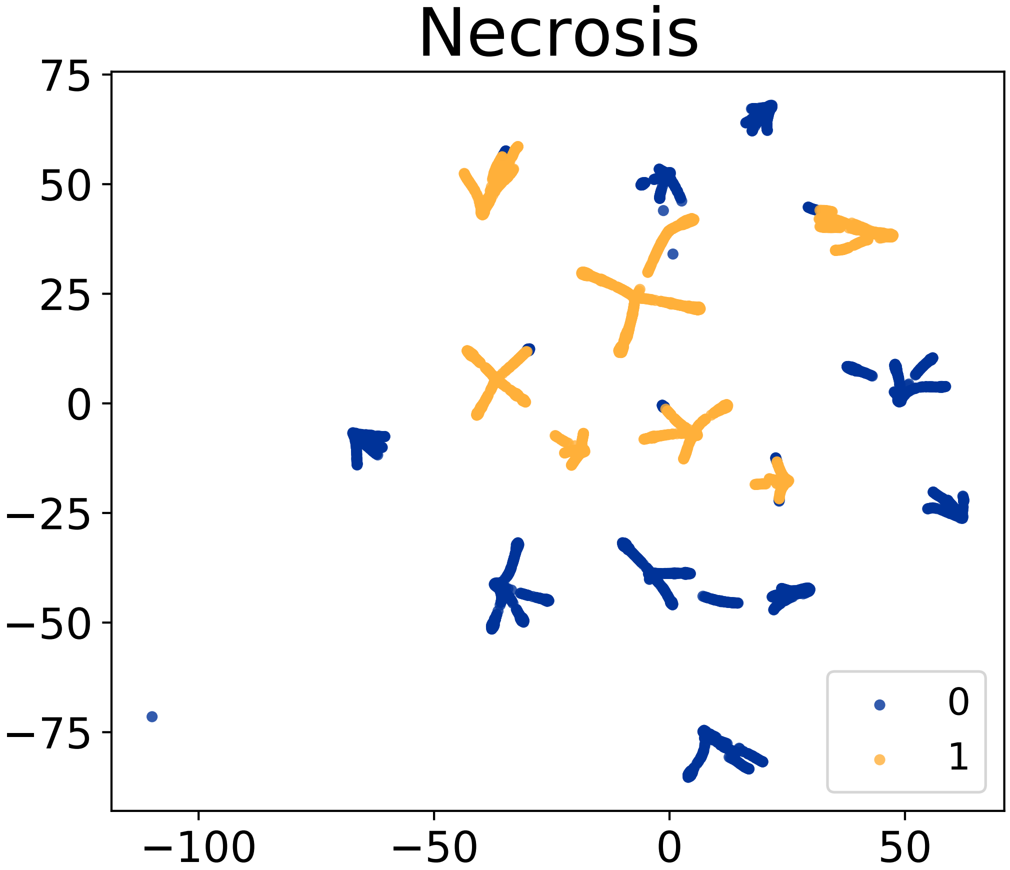

S2 Fig (n): The t-SNE visualization of necrosis raw features on kidney data.

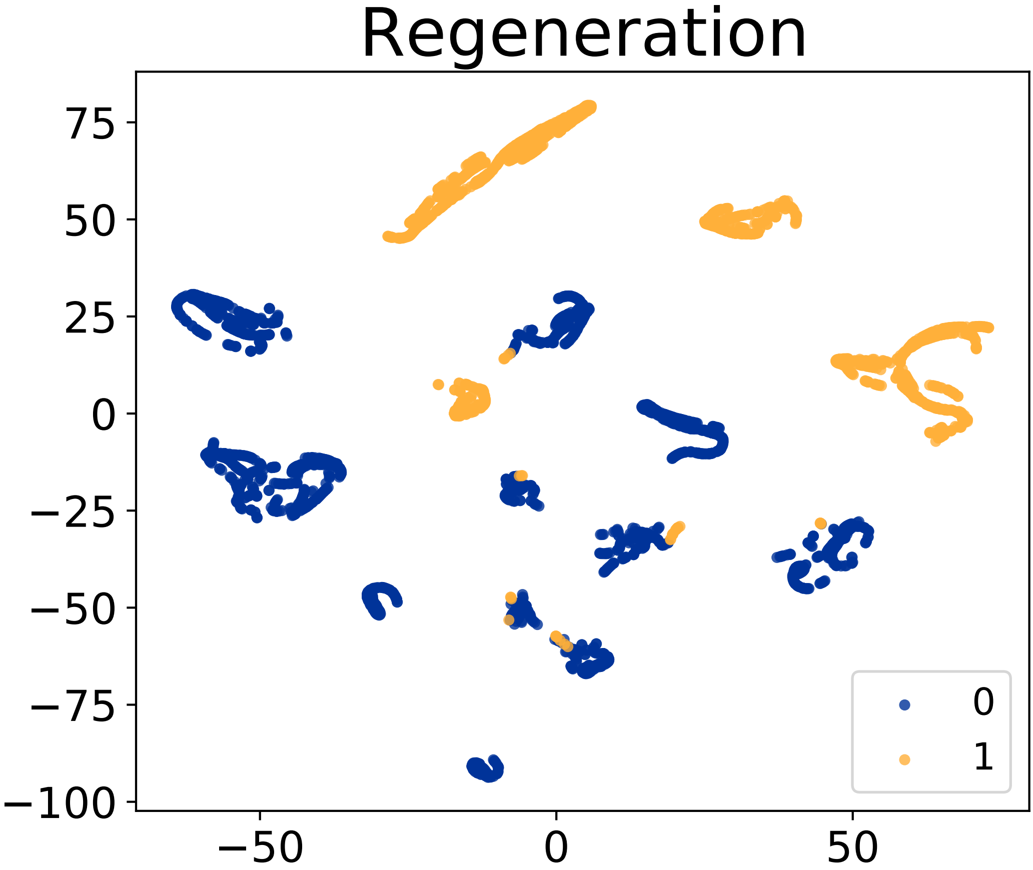

S2 Fig (o): The t-SNE visualization of regeneration features generated after RNN layer on kidney data.

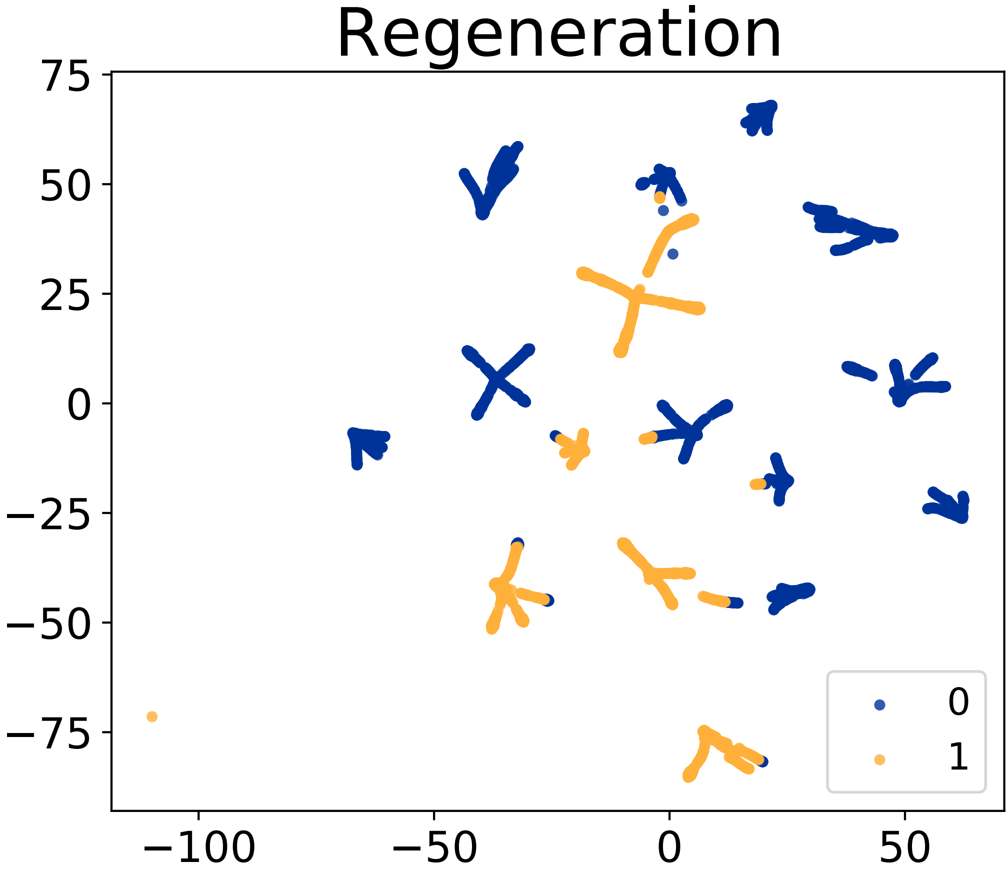

S2 Fig (p): The t-SNE visualization of regeneration raw features on kidney data.
