# Supplementary material for: A multi-label learning model for predicting drug-induced pathology in multi-organ based on toxicogenomics data
Source: PLoS Comput Biol. 2022 Sep 7;18(9):e1010402. doi: 10.1371/journal.pcbi.1010402 (PMC9451100; doi:10.1371/journal.pcbi.1010402)
Supplement: S3 Fig — (PDF) [file pcbi.1010402.s007.pdf]

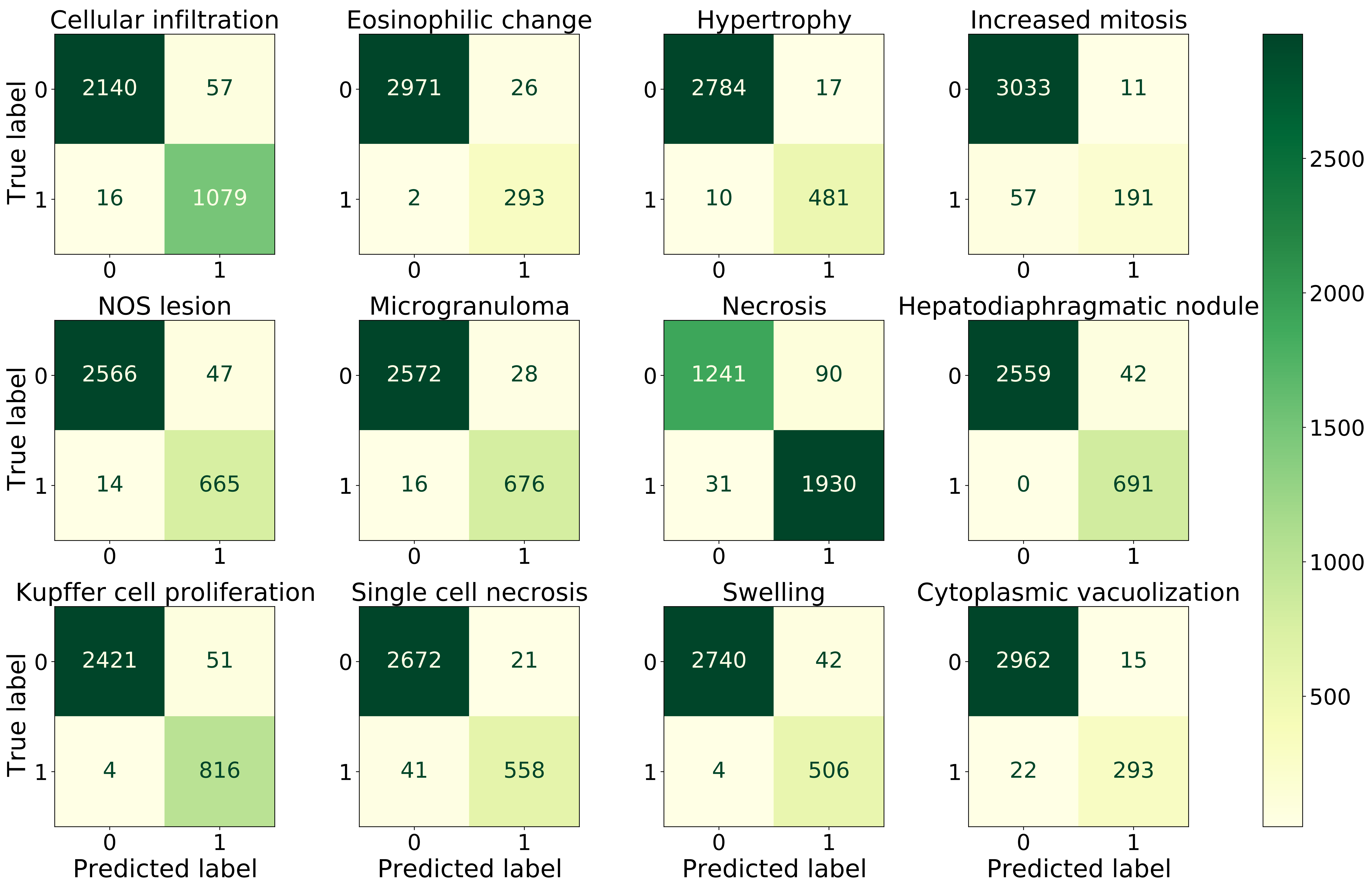

S3 Fig (a): The confusion matrix of pathology classification in the proposed Att-RethinkNet model. The top-left represents the TN, the top-right represents the FP, the bottom-left is FN and the bottom-right is TP. This figure shows the confusion matrix on liver data of fold 1.

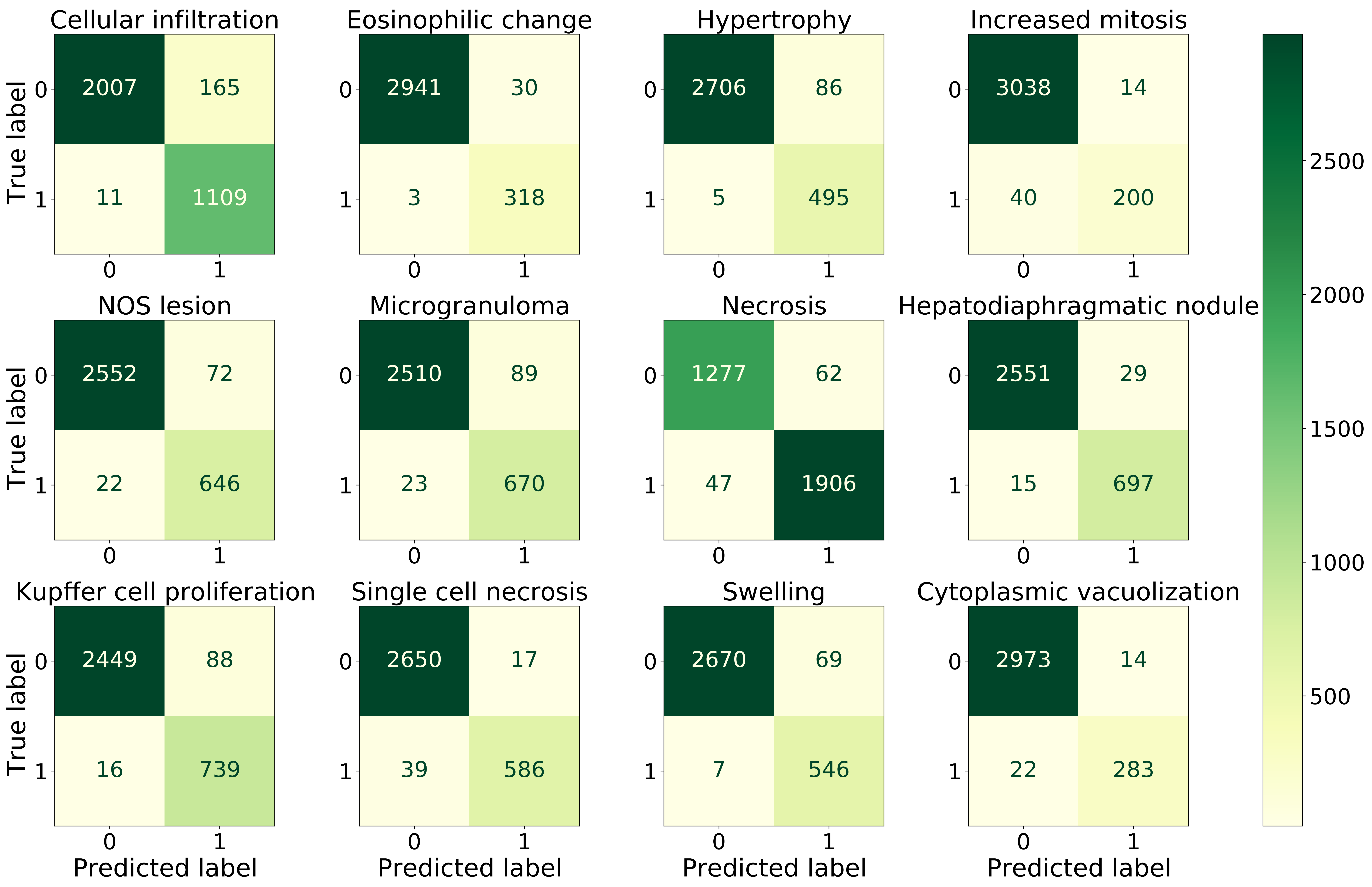

S3 Fig (b): The confusion matrix of pathology classification in the proposed Att-RethinkNet model. The top-left represents the TN, the top-right represents the FP, the bottom-left is FN and the bottom-right is TP. This figure shows the confusion matrix on liver data of fold 2.

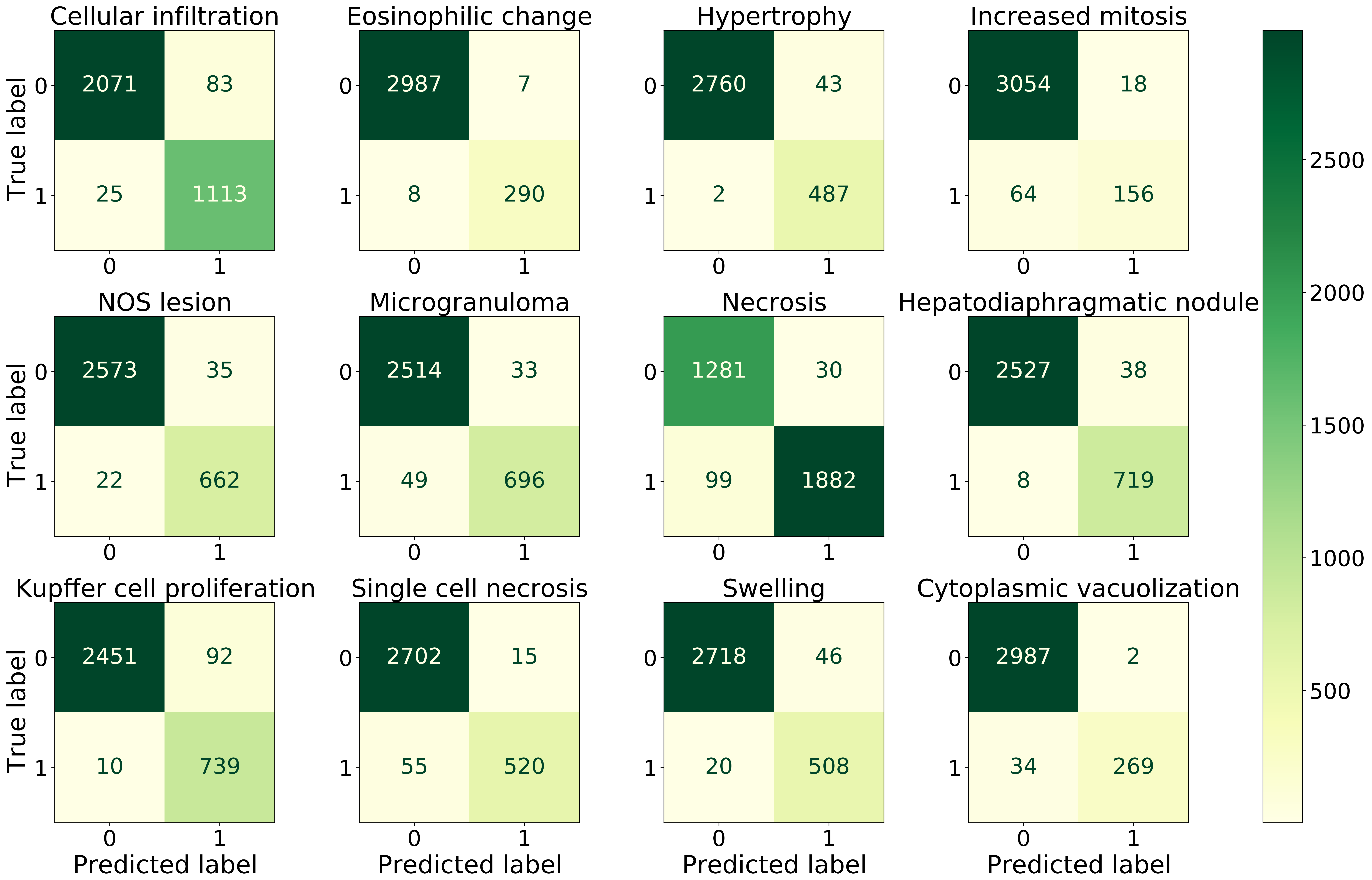

S3 Fig (c): The confusion matrix of pathology classification in the proposed Att-RethinkNet model. The top-left represents the TN, the top-right represents the FP, the bottom-left is FN and the bottom-right is TP. This figure shows the confusion matrix on liver data of fold 3.

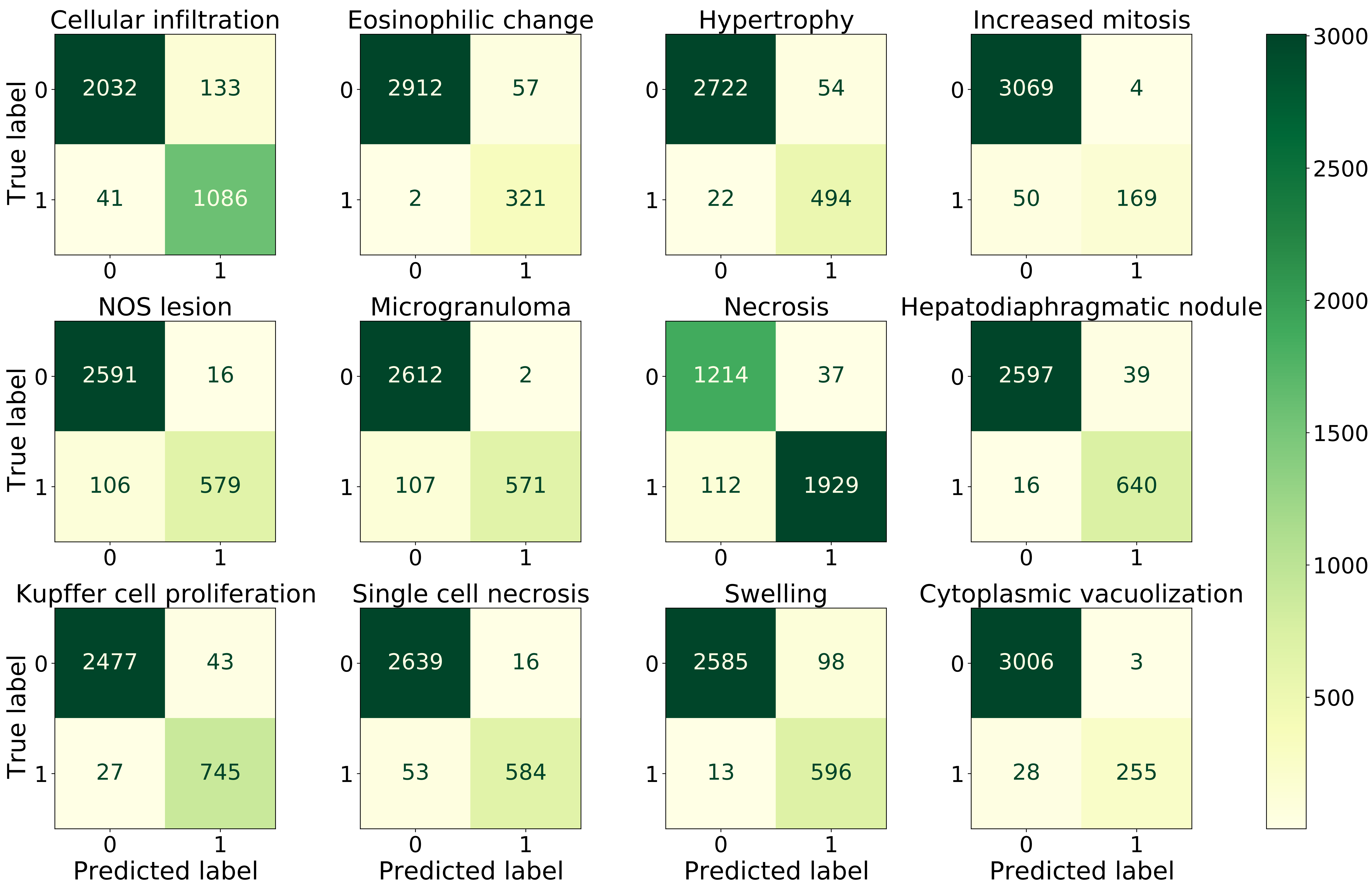

S3 Fig (d): The confusion matrix of pathology classification in the proposed Att-RethinkNet model. The top-left represents the TN, the top-right represents the FP, the bottom-left is FN and the bottom-right is TP. This figure shows the confusion matrix on liver data of fold 4.

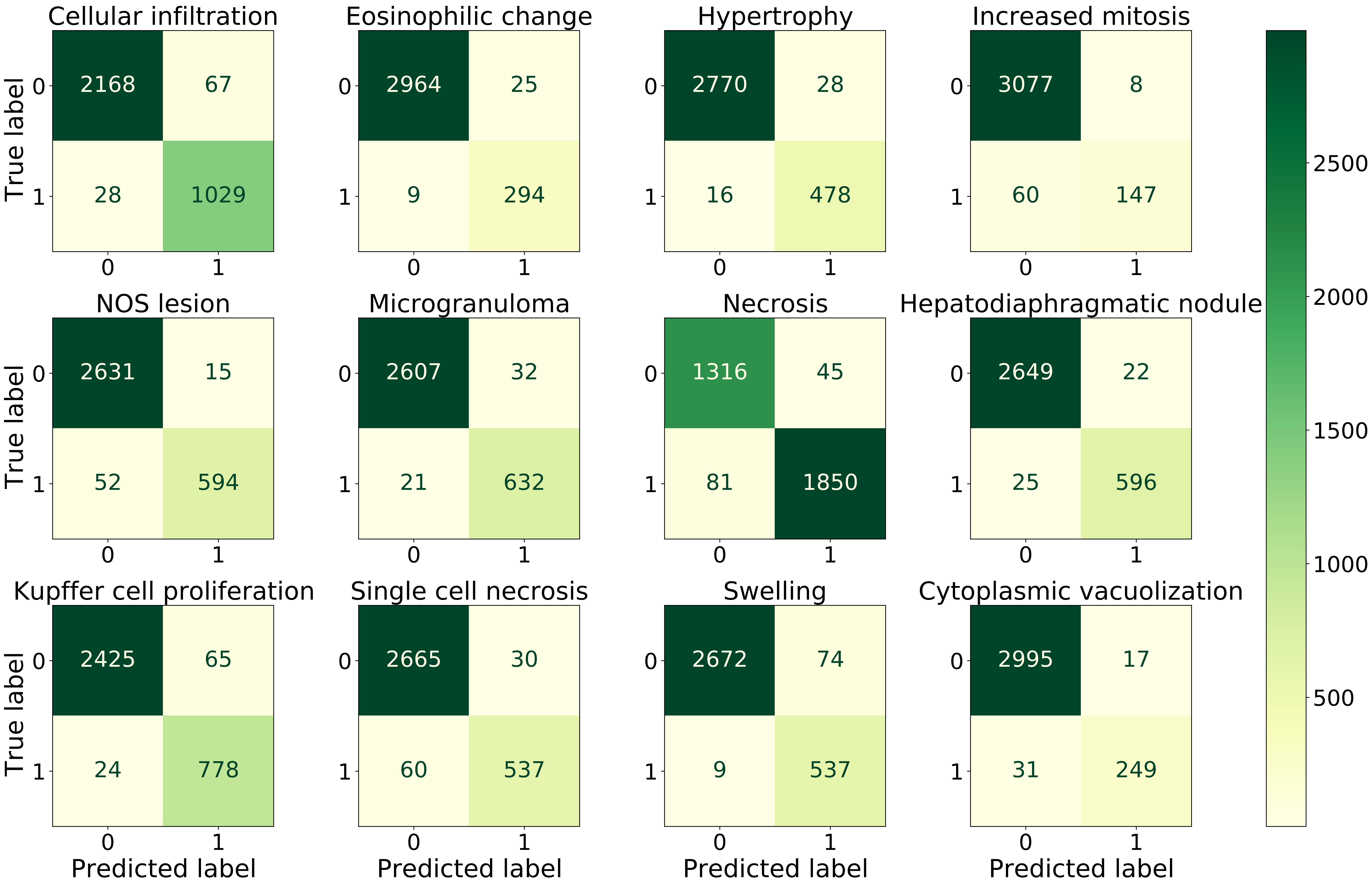

S3 Fig (e): The confusion matrix of pathology classification in the proposed Att-RethinkNet model. The top-left represents the TN, the top-right represents the FP, the bottom-left is FN and the bottom-right is TP. This figure shows the confusion matrix on liver data of fold 5.

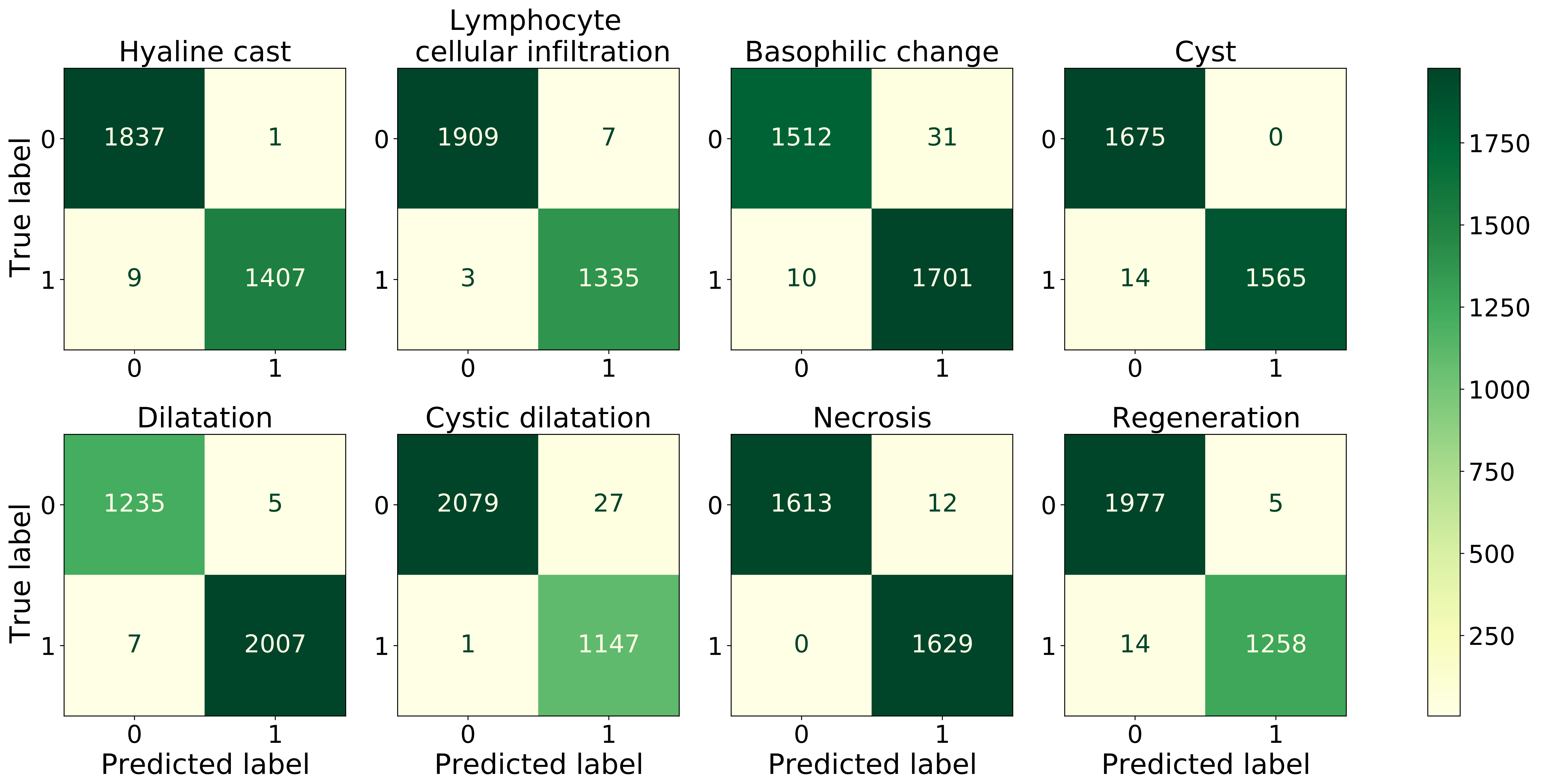

S3 Fig (f): The confusion matrix of pathology classification in the proposed Att-RethinkNet model. The top-left represents the TN, the top-right represents the FP, the bottom-left is FN and the bottom-right is TP. This figure shows the confusion matrix on kidney data of fold 1.

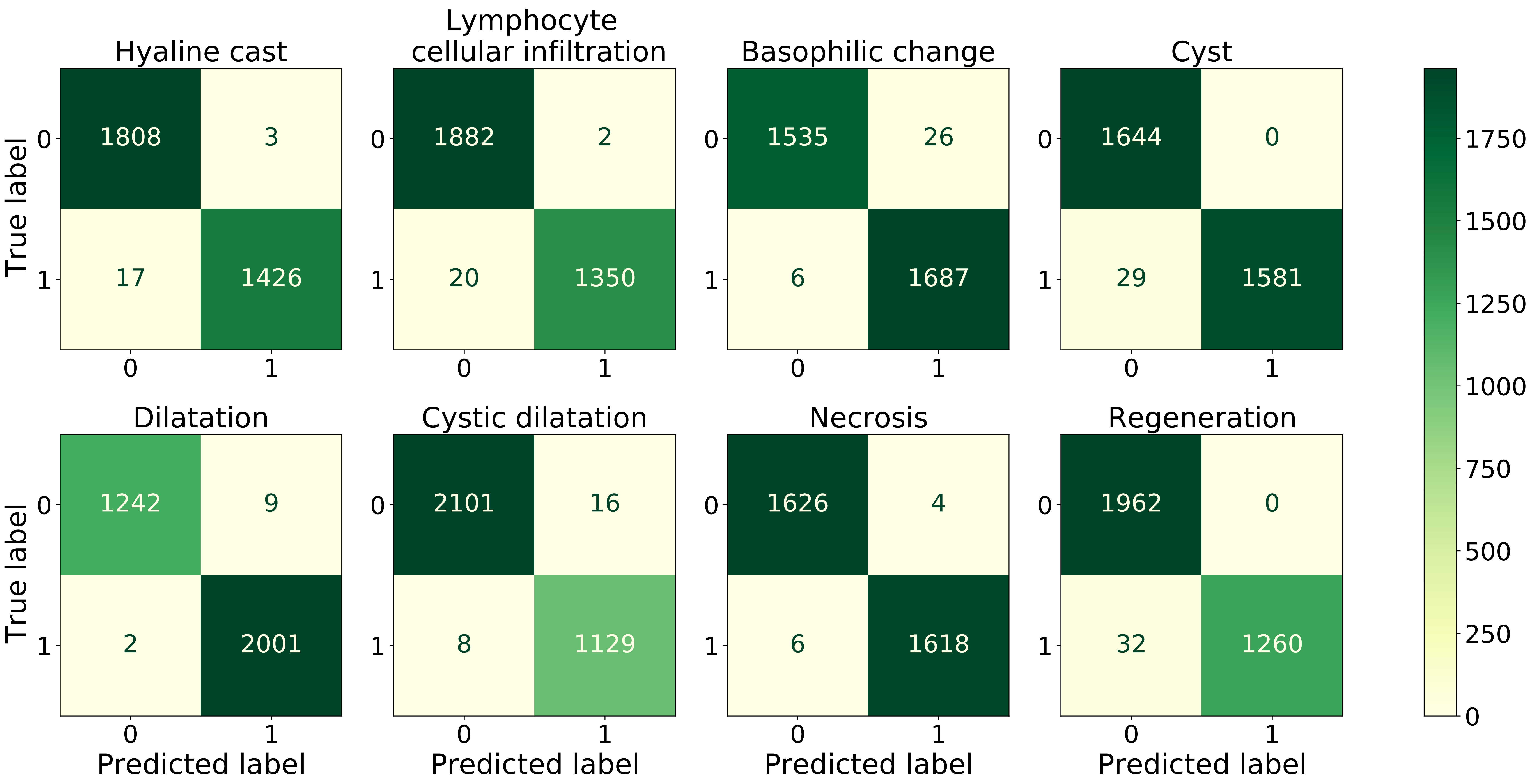

S3 Fig (g): The confusion matrix of pathology classification in the proposed Att-RethinkNet model. The top-left represents the TN, the top-right represents the FP, the bottom-left is FN and the bottom-right is TP. This figure shows the confusion matrix on kidney data of fold 2.

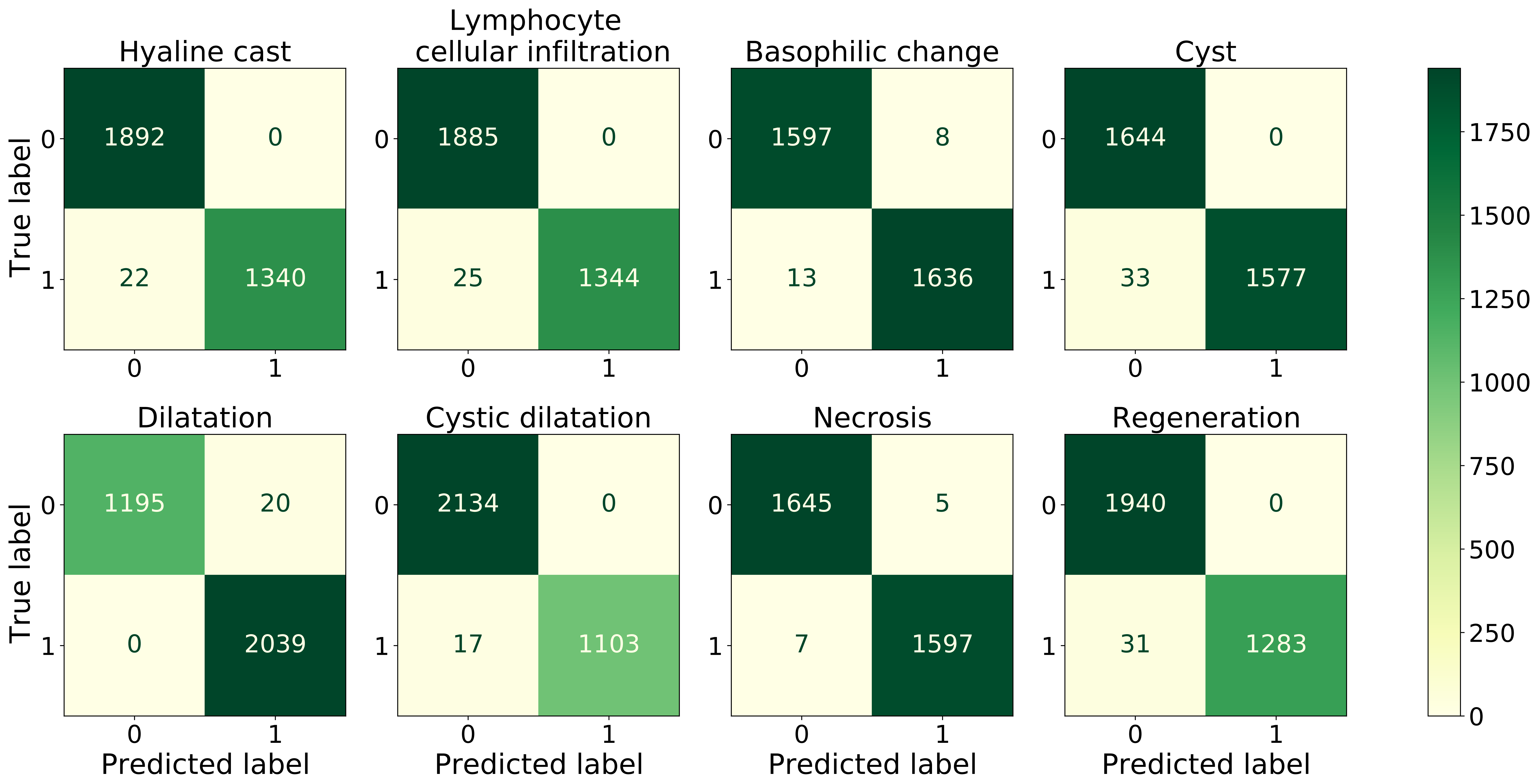

S3 Fig (h): The confusion matrix of pathology classification in the proposed Att-RethinkNet model. The top-left represents the TN, the top-right represents the FP, the bottom-left is FN and the bottom-right is TP. This figure shows the confusion matrix on kidney data of fold 3.

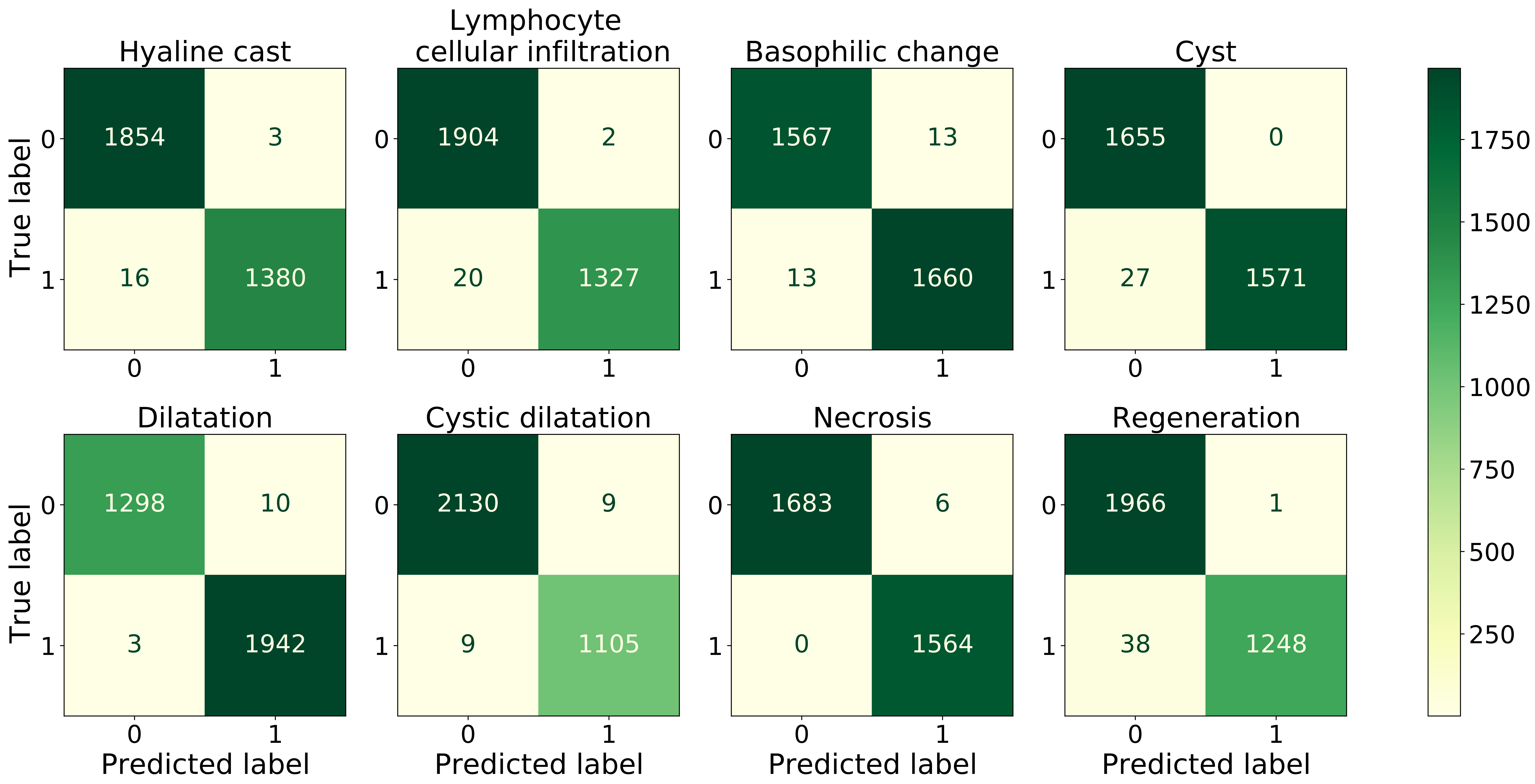

S3 Fig (i): The confusion matrix of pathology classification in the proposed Att-RethinkNet model. The top-left represents the TN, the top-right represents the FP, the bottom-left is FN and the bottom-right is TP. This figure shows the confusion matrix on kidney data of fold 4.

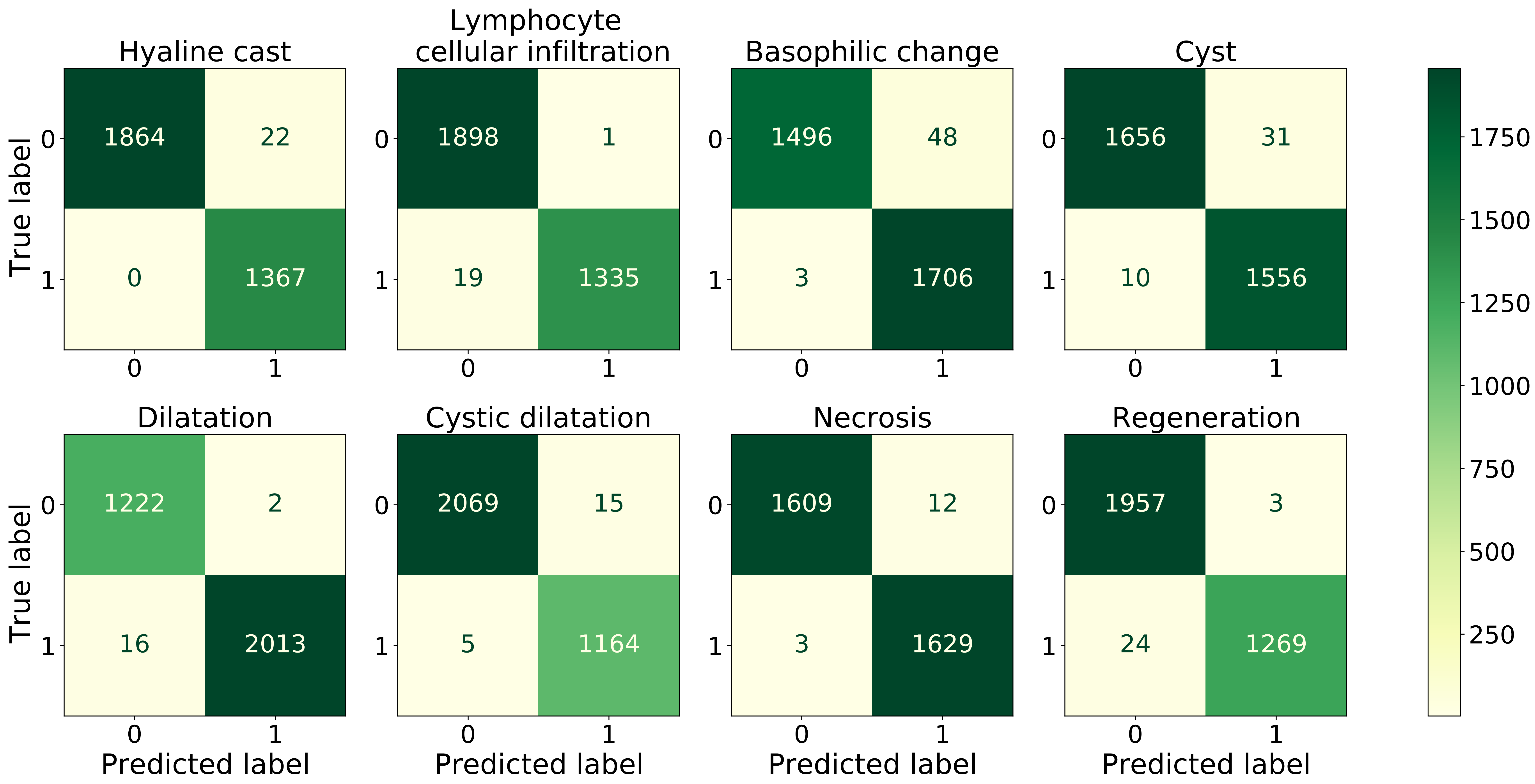

S3 Fig (j): The confusion matrix of pathology classification in the proposed Att-RethinkNet model. The top-left represents the TN, the top-right represents the FP, the bottom-left is FN and the bottom-right is TP. This figure shows the confusion matrix on kidney data of fold 5.
