# Supplementary material for: A multi-label learning model for predicting drug-induced pathology in multi-organ based on toxicogenomics data
Source: PLoS Comput Biol. 2022 Sep 7;18(9):e1010402. doi: 10.1371/journal.pcbi.1010402 (PMC9451100; doi:10.1371/journal.pcbi.1010402)
Supplement: S4 Fig — (PDF) [file pcbi.1010402.s008.pdf]

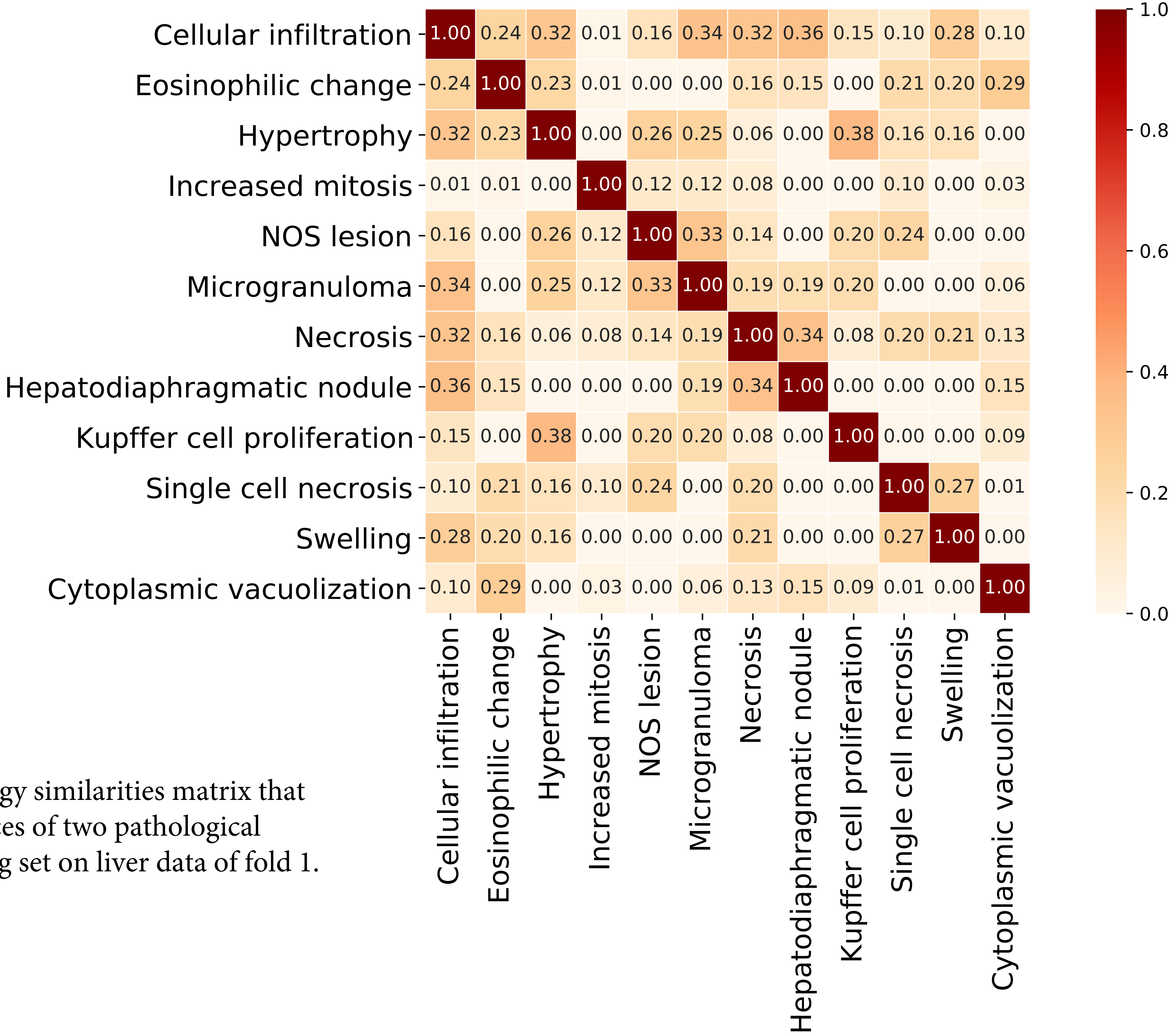

S4 Fig (a): The pathology similarities matrix that describes co-occurrences of two pathological findings within training set on liver data of fold 1.

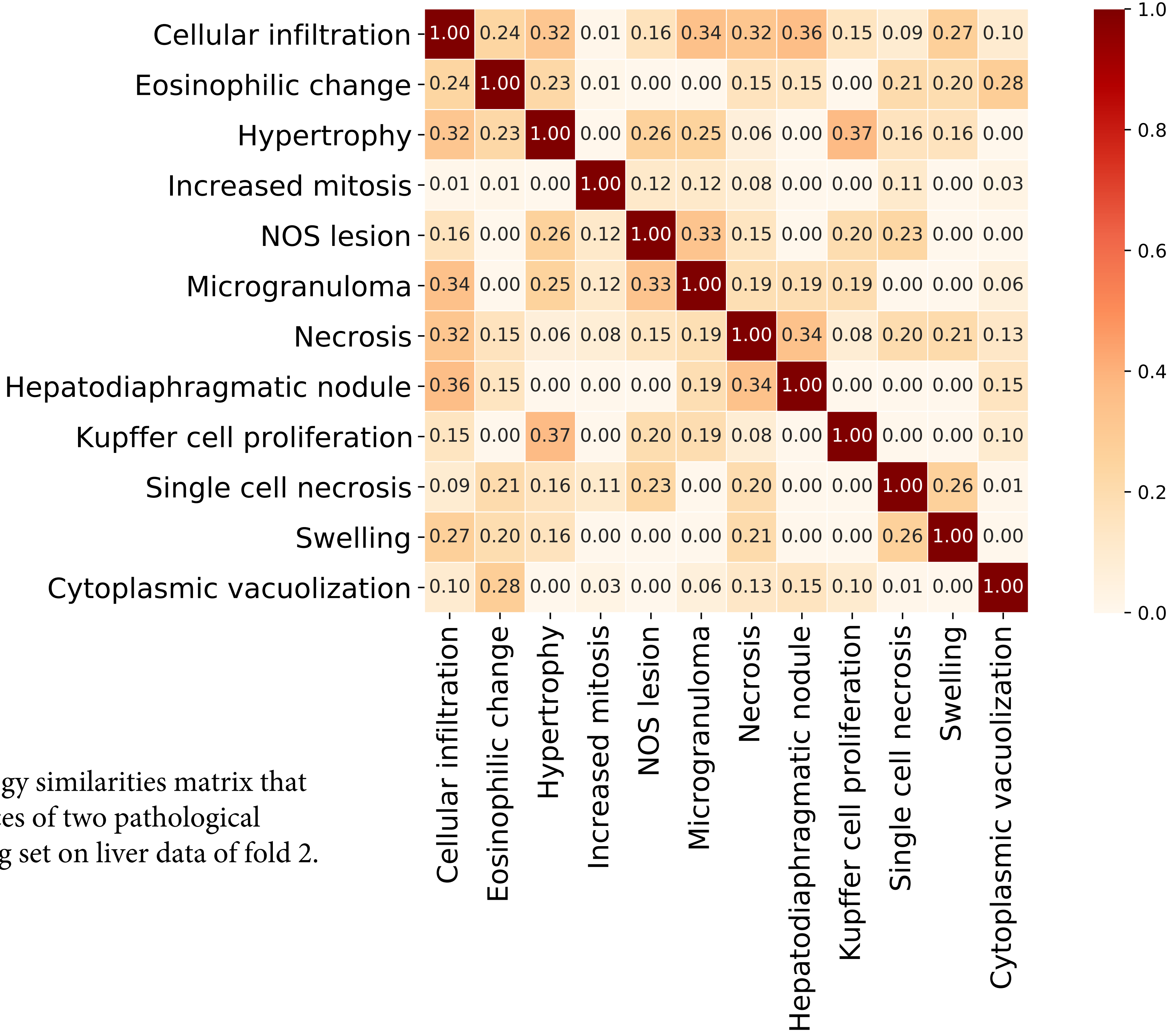

S4 Fig (b): The pathology similarities matrix that describes co-occurrences of two pathological findings within training set on liver data of fold 2.

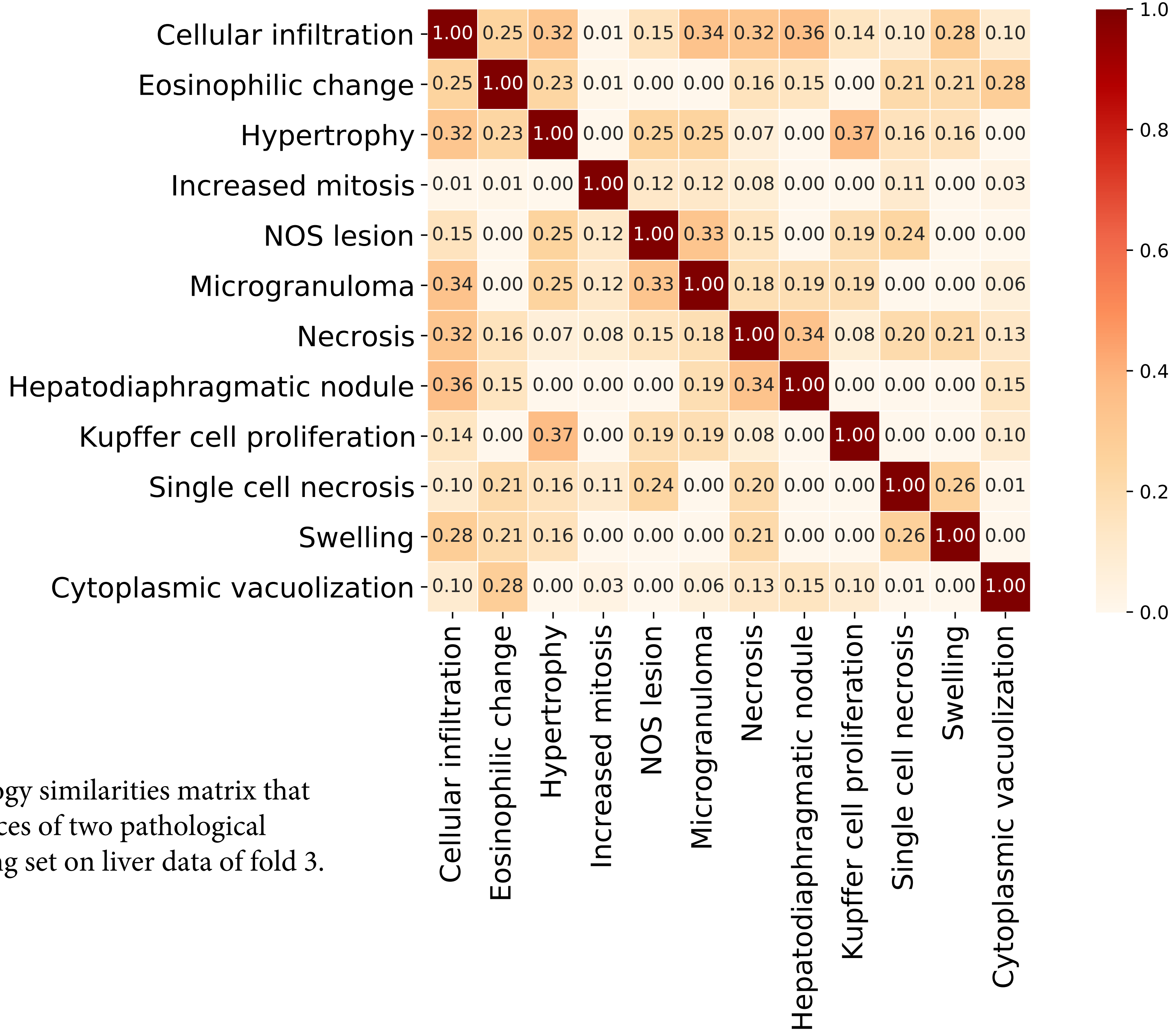

S4 Fig (c): The pathology similarities matrix that describes co-occurrences of two pathological findings within training set on liver data of fold 3.

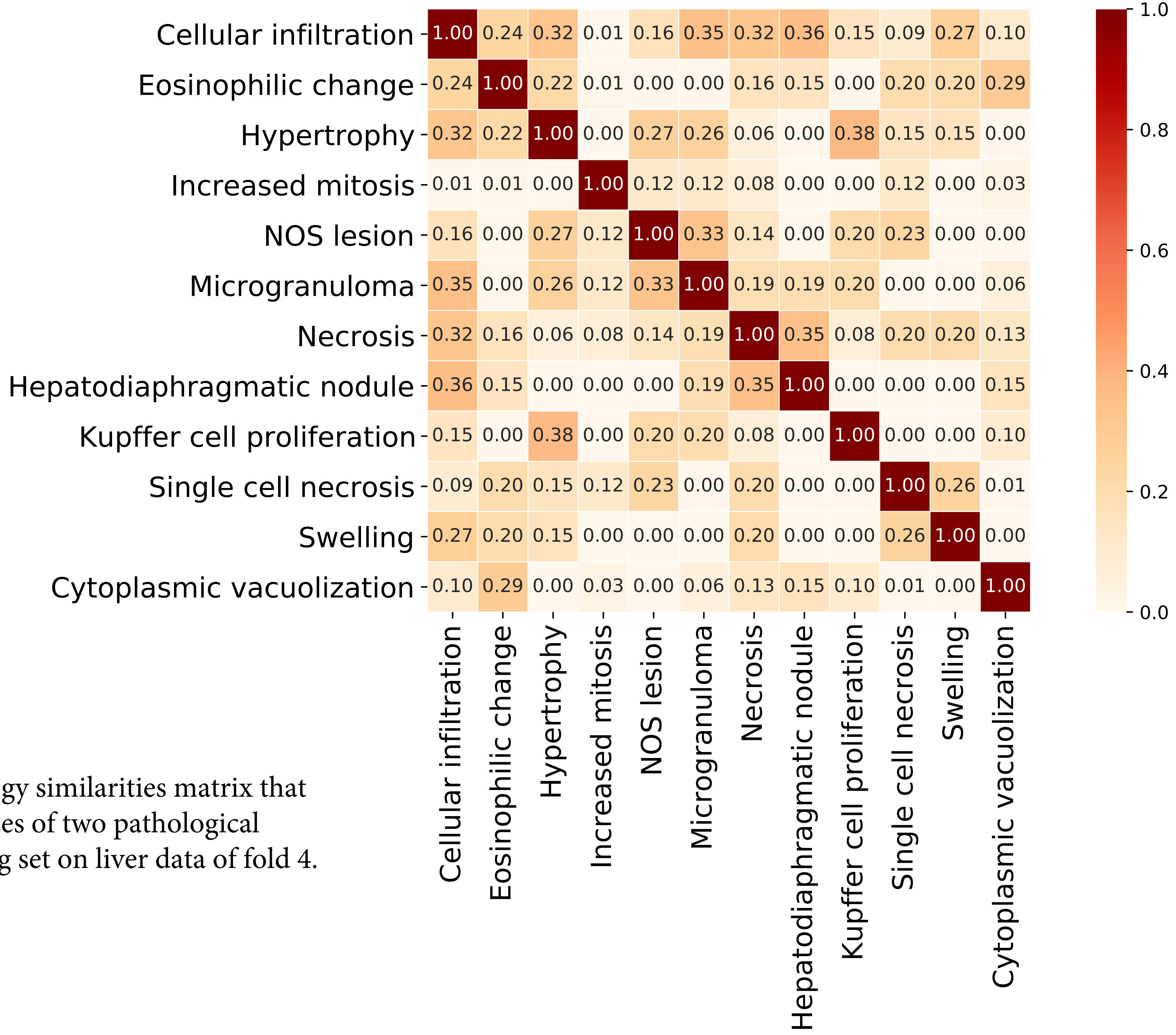

S4 Fig (d): The pathology similarities matrix that describes co-occurrences of two pathological findings within training set on liver data of fold 4.

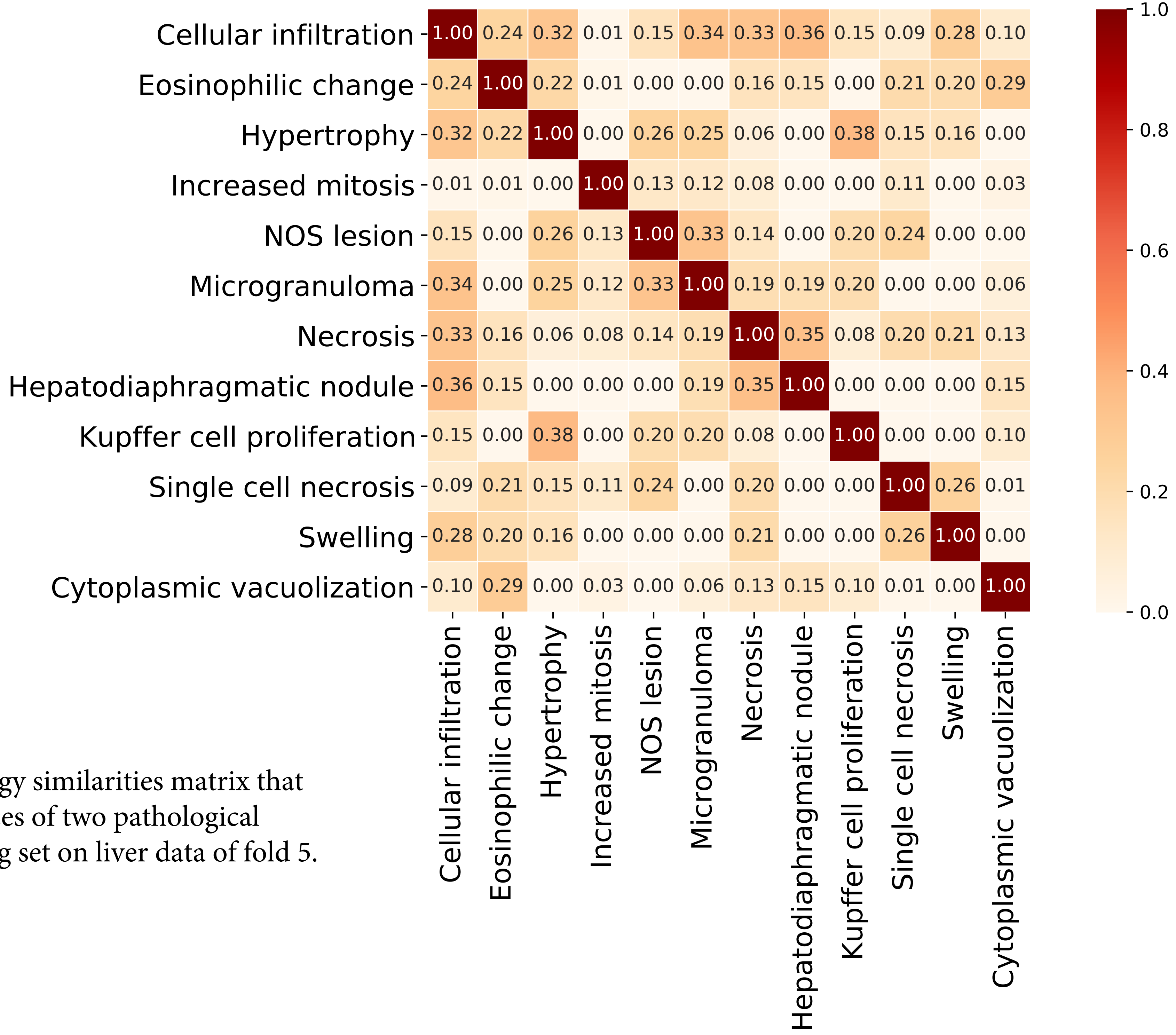

S4 Fig (e): The pathology similarities matrix that describes co-occurrences of two pathological findings within training set on liver data of fold 5.

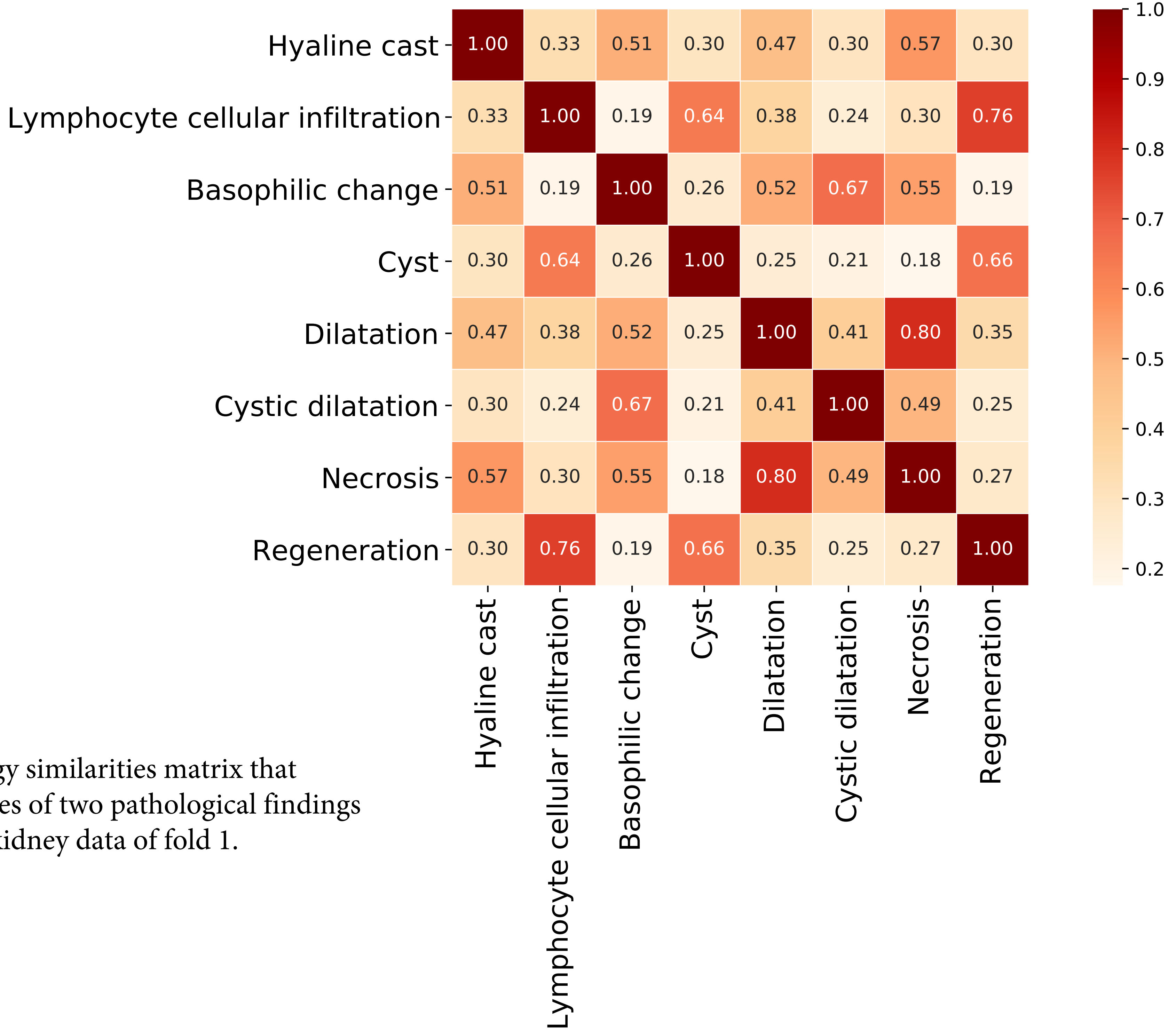

S4 Fig (f): The pathology similarities matrix that describes co-occurrences of two pathological findings within training set on kidney data of fold 1.

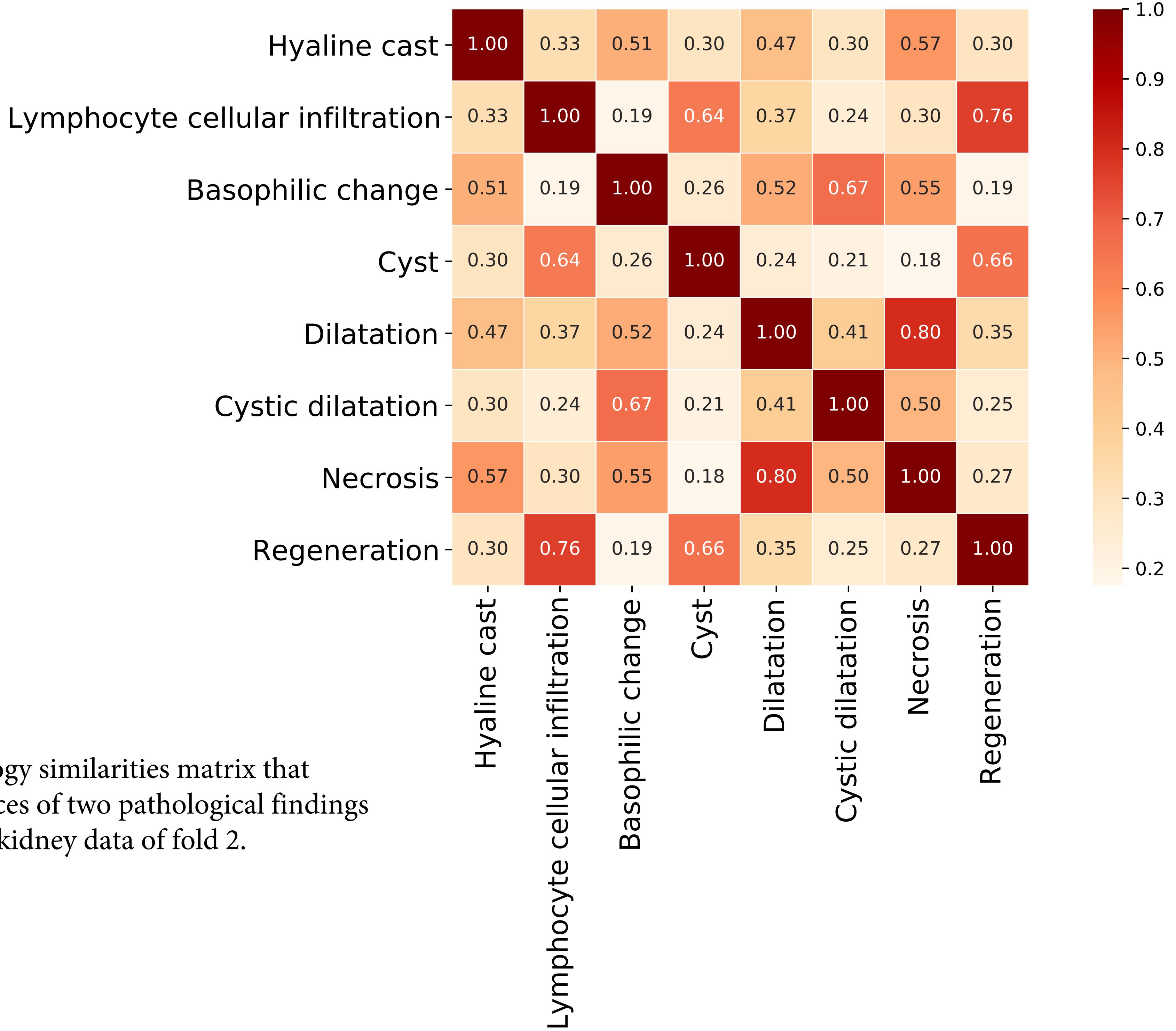

S4 Fig (g): The pathology similarities matrix that describes co-occurrences of two pathological findings within training set on kidney data of fold 2.

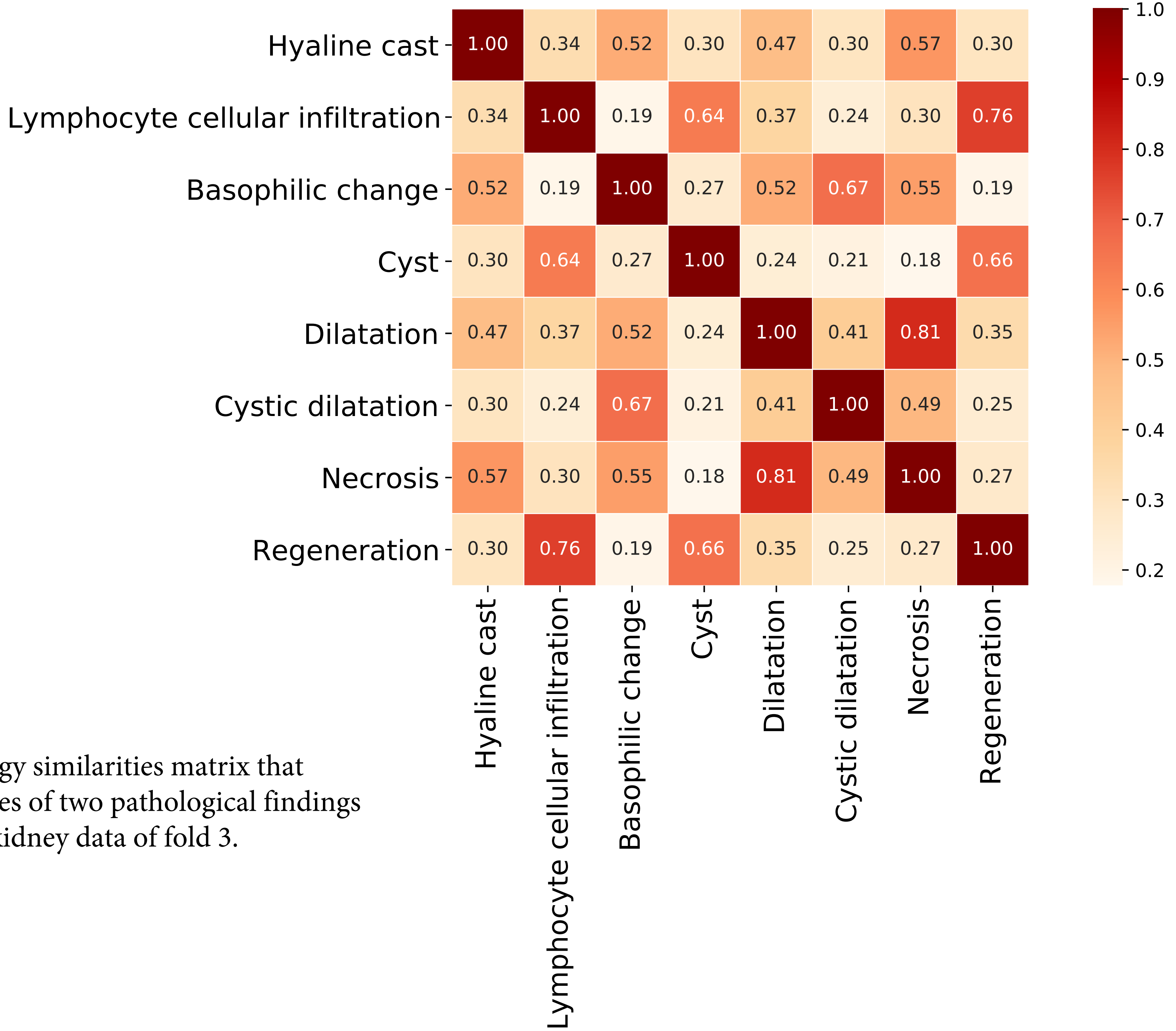

S4 Fig (h): The pathology similarities matrix that describes co-occurrences of two pathological findings within training set on kidney data of fold 3.

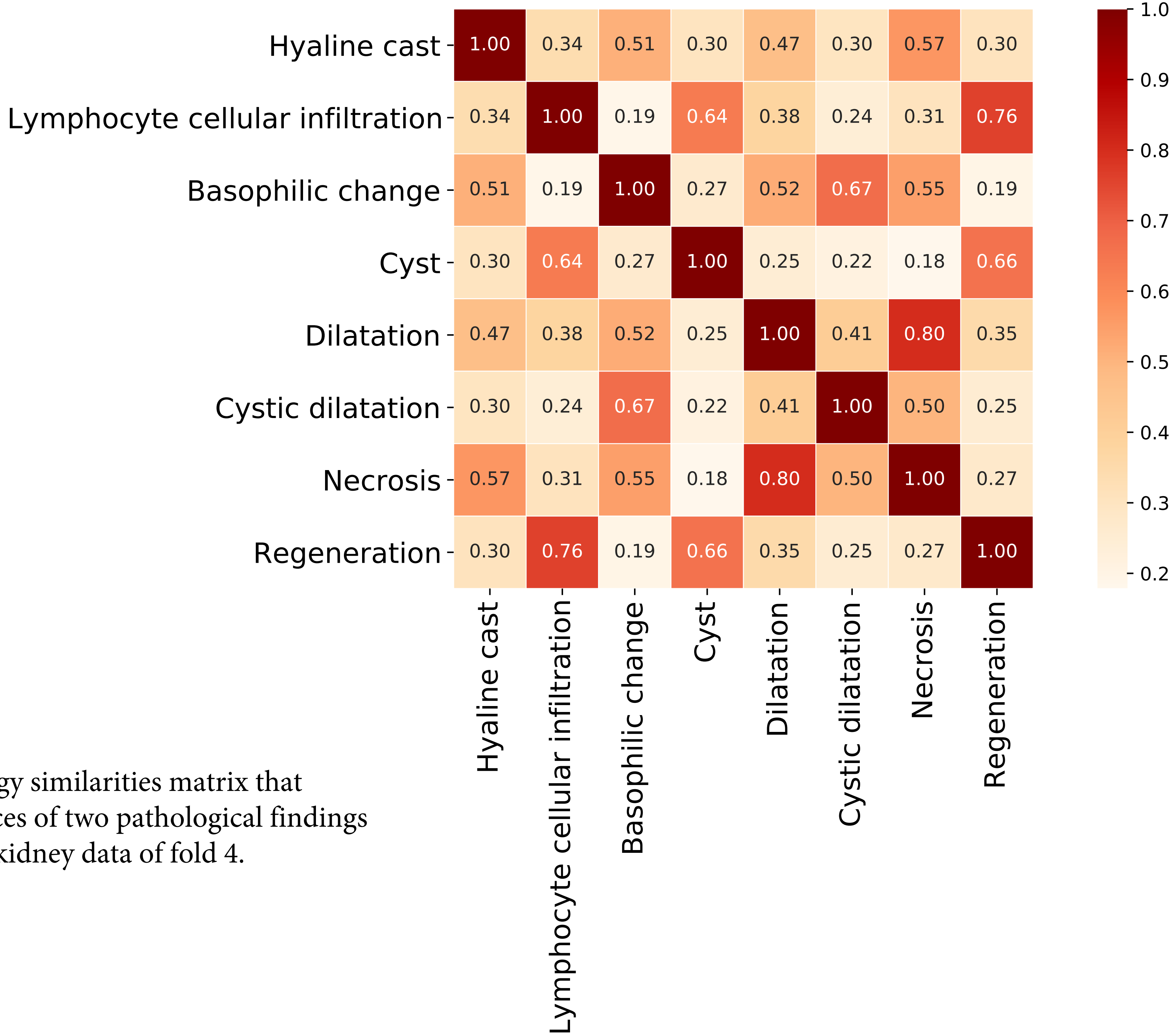

S4 Fig (i): The pathology similarities matrix that describes co-occurrences of two pathological findings within training set on kidney data of fold 4.

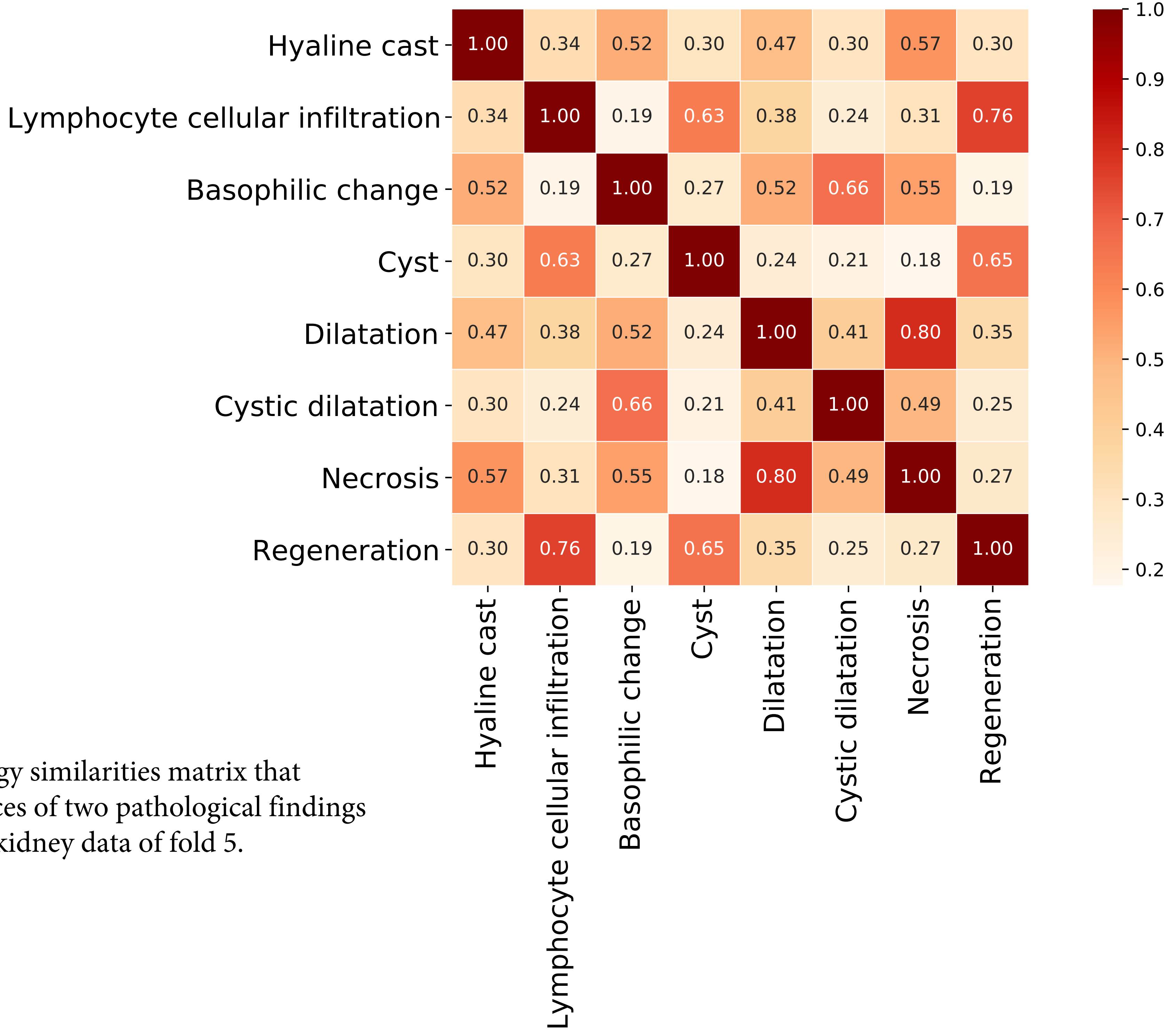

S4 Fig (j): The pathology similarities matrix that describes co-occurrences of two pathological findings within training set on kidney data of fold 5.
